# Elucidating the mechanism and origins of selectivity on catalyst-dependent cyclization reactions to form polycyclic indolines from a theoretical study†

**DOI:** 10.1039/d1ra01632f

**Published:** 2021-06-09

**Authors:** Yan Zhang, Yongsheng Yang, Ying Xue

**Affiliations:** College of Chemistry, Key Lab of Green Chemistry and Technology in Ministry of Education, Sichuan University Chengdu 610064 People's Republic of China yxue@scu.edu.cn +86 28 85418330

## Abstract

There is a significant role for bioactive polycyclic indolines in the pharmaceutical science field. In this paper, a systematic DFT study at the M06-D3/SMD/BS2//B3LYP-D3/BS1 level is adopted to investigate the cyclization reaction catalyzed by Rh_2_(esp)_2_ and InCl_3_ to generate polycyclic indolines. Luckily, the simplification of the Rh_2_(esp)_2_ computational model is feasible, and successfully used in this study. The computational results detailed indicate the reaction mechanisms catalyzed by different catalysts, and the regio- and diastereo-selectivity. The regio-selectivity is controlled by the weak interaction (reflected in repulsive interaction) of the key transition state in the InCl_3_-catalyzed pathway, and the larger distortion energy makes the regio-selectivity more obvious in the pathway catalyzed by Rh_2_(esp)_2_. It is important that this theoretical study suggests the significance of the catalyst in the reaction system in detail by NBO and FMO analysis. This paper is a good explanation of the experimental phenomenon caused by the catalyst where InCl_3_ is more significant than Rh_2_(esp)_2_. The reaction mechanism and the importance of the catalysts are revealed in detail by this particular theoretical study.

## Abstract 2

The mechanism and origins of selectivities of multiply catalyzed cyclization reactions catalyzed by Rh_2_(esp)_2_ and InCl_3_ are investigated by DFT calculations as well as distortion/interaction and noncovalent interaction (NCI) analyses.

## Graphical abstract

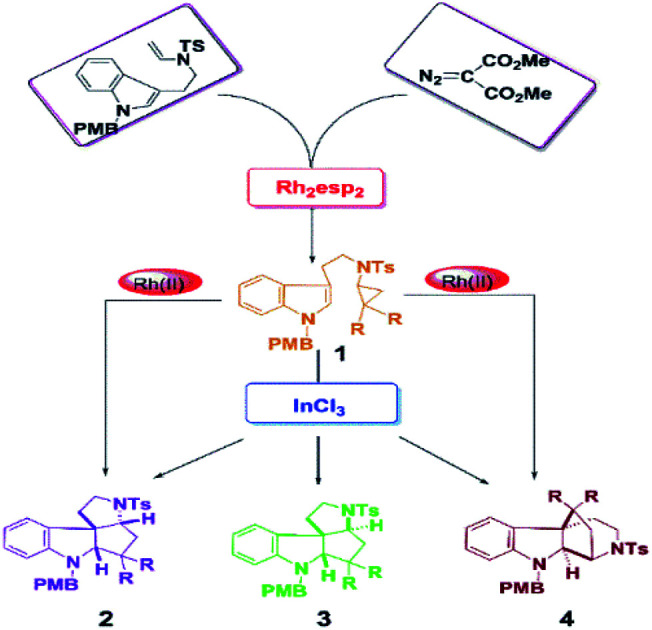

## Introduction

Polycyclic compounds, ubiquitous products in nature, are widely used in the scientific field. Among them, the polycyclic aromatic hydrocarbons (PAHs) are the important basic materials of industry, and are obtained from burning coal and some organic substances.^1^ And biologically active matter draws more attention of scientists in the medicine and chemistry fields such as heterocyclic spiroindolines and indoline alkaloids, which often exist in natural products and pharmaceutical molecules.^2^ Therefore, it is a challenge to chemists to synthesis polycyclic compounds, especially bioactive molecules, economically and effectively.^2*b*^ Until now, a classical way to acquire bioactive polycyclic compounds is the cascade reaction by forming them step by step.^3^ For instance, the Yue group reported the enantioselective synthesis of polycyclic spirooxindoles by the cascade reaction between 3-isothiocyanato oxindoles and 3-nitroindoles.^4^ Meanwhile, there are some original ways emerging into our sight, for example, the aryne-based strategy reported by Takikawa and co-workers, with more efforts devoted to synthesis methods.^5^ Based on the method reported by Takikawa,^5^ Ikawa *et al.* obtained polycyclic compounds by regioselective synthesis with 1,3- and 1,4-benzdiyne equivalents through starting with benzannulations and then ring-enlargement.^6^

**Scheme 1:**
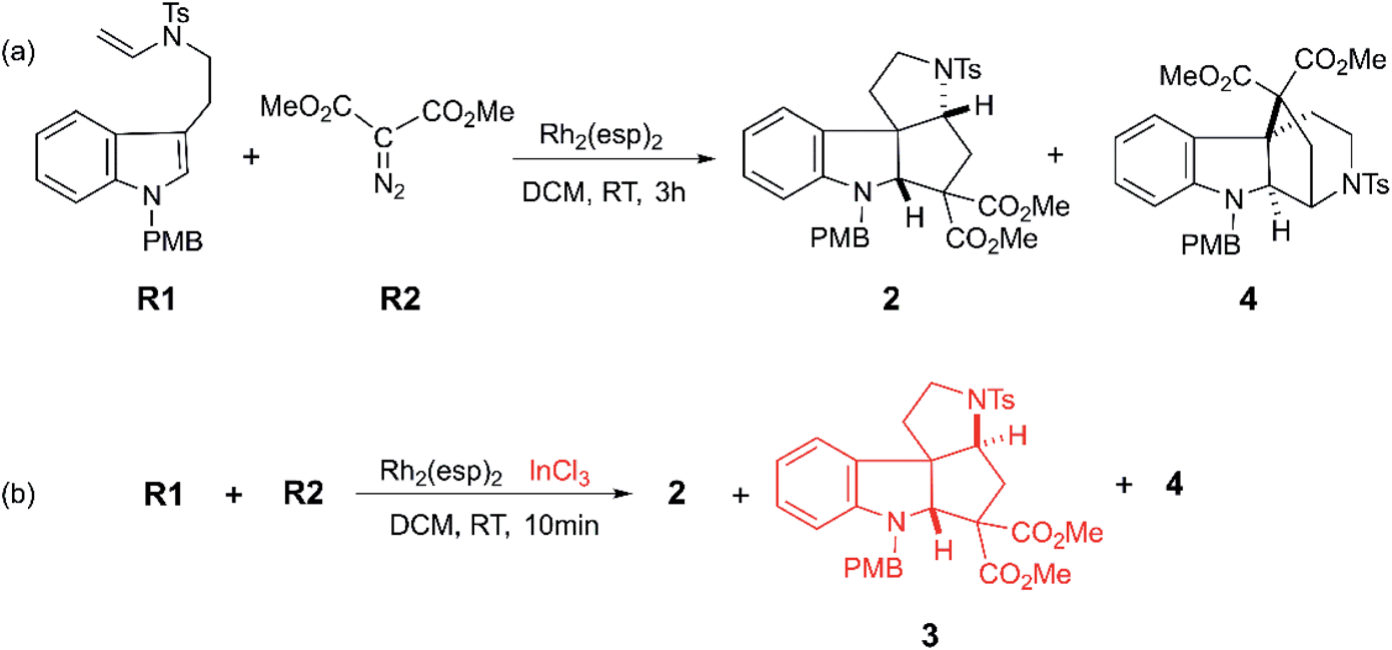
The cyclization reactions catalyzed by the Rh_2_(esp)_2_ and InCl_3_ to obtain polycyclic compounds reported by Tang group.^16^

Moreover, with the rapid development of organic catalysts and transition metal catalysts, it is a good way to adopt catalysts to prepare polycyclic compound quickly and efficiently.^7^ The organic phosphine^8^ with high catalytic activity has displayed the good performance in annulation reaction. Specially, some transition metal catalysts such as platinum,^9^ rhodium,^10^ palladium,^11^ ruthenium,^12^ gold^7*b*,13^ and copper^14^ have received wide interest and been found to possess great catalytic performance for the synthesis of functionalized polycyclic compounds. However, it is limited for homogenous catalysts to catalyze reactions with multiple processes, because the catalytic characteristics of a variety of catalysts are much different. Owing to these huge distinction in activity and selectivity, the reactions can obtain various products with the same substrates catalyzed by diverse catalysts.^15^ So as to say, in a degree, there would have much better catalytic performance with multiple catalysts, as Tang group reported.^16^ They explored the Rh(ii)-carbene triggered cyclopropanation between tryptamine-derived enamides R1 and dimethyl diazomalonate R2 to get polycyclic indolines (shown as Scheme 1). In their experiment, two polycyclic products (2 and 4) were obtained in 3 hours when there was only Rh_2_(esp)_2_ catalyst. While, if the InCl_3_ coexisted, the reaction time shortened to 10 min, and the polycyclic products are richer, where three kinds of polycyclic indolines (2, 3, and 4) were isolated by adjusting the solvent and experiment temperature. It is worthy to note that they detected one key intermediate, a cyclopropanation compound generated by Rh(ii)-carbene complex. Meanwhile, the important cyclopropanation compound intermediate is always observed between the interaction of Rh(ii)-carbene and terminal alkenyl. Moreover, it is a classical cyclopropanation reaction formed by Rh(ii)-carbenoid interacted with terminal alkenyl group,^17^ and the details were reported repeatedly by theoretical study.^18^ Also, the mechanism of this cyclization reaction which involves two key parts was assumed by the experiment authors.^16^ One is the formation of cyclopropanation compound catalyzed by Rh_2_(esp)_2_, and the other is the way from the key intermediate to diverse polycyclic indolines catalyzed by InCl_3_. However, there is something we cannot understand clearly: (1) the mechanistic details for this catalytic reaction; (2) the role and effect of the catalysts (Rh_2_(esp)_2_ and InCl_3_) in this system; (3) the origins of the diastereo-selectivity and regio-selectivity of the cyclization reaction. Finally, the kinetical and thermodynamic properties of the reaction are still unclear.

Based on above questions, the density functional theory (DFT)^19^ calculation was performed to investigate this catalytic reaction in a deeper sight. Before this paper, a series of theoretical studies involved Rh(ii)-carbene were reported by our group. For example, the [3 + 3]-cycloaddition reaction catalyzed by Rh_2_(OAc)_4_,^18*a*^ and Rh(ii)-carbenoid selectively triggering [3 + 2]-, [5 + 1]-cycloaddition reaction.^18*b*^ These theoretical studies made the reaction phenomenon and the origin of selectivity observed in experiments clear. Likewise, more and more theoretical studies spring up and demonstrate their reliability. Therefore, the cyclic reaction reported by Tang group^16^ draws our attention. In this paper, the details of reaction mechanism are firstly reported, and the questions that we put forward are also solved by our calculations. This paper will give more details of this catalytic reaction in molecule level and hope to provide some suggestion for future studies.

## Computational details

The Gaussian 09 package^20^ was utilized for all of the DFT calculations here. All of the geometry optimizations were carried out in the gas phase using the B3LYP hybrid functional^21^ accompanied with D3 Grimme dispersion correction^22^ as well as a mixed basis set of Lanl2dz for Rh and In atoms and 6-31G(d,p) for the other atoms (C, H, O, N, S, and Cl), which is abbreviated as B3LYP-D3/BS1. In order to confirm whether the stationary point structures are transition states with only one imaginary and minima without imaginary, the harmonic vibrational frequency analysis was conducted. Meanwhile, the intrinsic reaction coordinate (IRC)^23^ calculations were performed to verify the key transition state structures connecting with correct reactants and products. Solvent effect in dichloromethane (*ε* = 8.93, the solvent used experimentally) was taken into consideration with SMD model^24^ in single-point energy calculations. To acquire better accuracy in energy, the precise functional method and larger basis set were adopted, the M06 functional^25^ with basis set of SDD for Rh and In atoms and 6-311+G(d,p) for all nonmetallic atoms, denoted as M06-D3/SMD/BS2, in single-point calculation for all the B3LYP-D3/BS1-optimized structures. By the way, the D3 empirical dispersion was used in the whole research. Natural bond orbital (NBO) analysis and the other auxiliary analysis were carried out at the level of B3LYP-D3/BS1. The Gibbs free energies reported in this paper are evaluated by the thermal corrections from the unscaled vibrational frequencies at the B3LYP-D3/BS1 level on the optimized geometries in the gas phase being added to the M06-D3/SMD/BS2 electronic energies in solvent phase.

## Results and discussion

### Simplified catalyst model

**Fig. 1:**
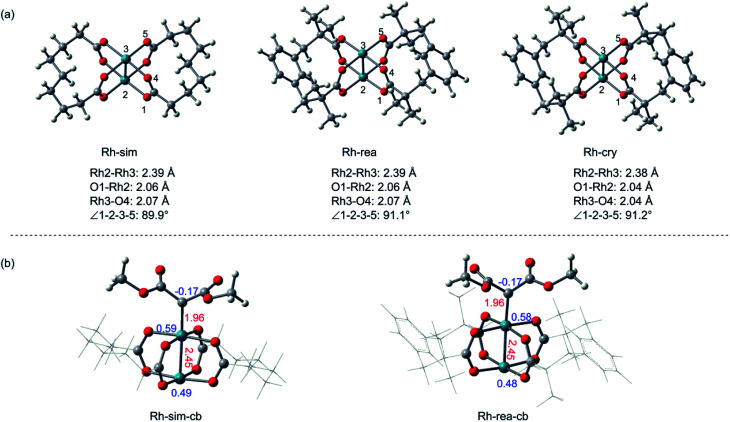
The comparison among the crystal, real and simplified catalyst models (a). The carbenoids formed by simplified and real Rh_2_(esp)_2_ (the red numbers are bond length in Å and the blue values represent atom charge) (b).

At first, the simplification of Rh_2_(esp)_2_ is necessary to be computationally tractable, due to its large ligand, and some simplification done for the structure of Rh_2_(esp)_2_ complex are displayed in Fig. 1. To verify the feasibility of the simplified model (Rh-sim), some comparisons with real catalyst model (Rh-rea) and catalyst crystal (Rh-cry) are taken into consideration, and the results are shown in Fig. 1. For Fig. 1(a), geometry structures are compared in here and there are some differences among three models. Seeing from the bond length, Rh–Rh bond in three structures is almost the same, and the little difference about 0.03 Å in Rh–O bond appears between the crystal and calculation models. Meanwhile, the difference of dihedral angle ∠1-2-3-5 is 1.3° from Rh-sim to Rh-cry. And these slight differences indicate the computational models are reasonable. Then, the electron effect of the simplified model has been taken into consideration, Fig. 1(b) shows the metal carbenoids generated by Rh-sim and Rh-rea models, and the values in red are bond lengths and blue numbers mean the atomic charges. As we can see, the corresponding bond lengths and charges for key atoms are almost the same between Rh-sim-cb and Rh-rea-cb. That's to say, the simplification of rhodium catalyst also makes no difference in carbenoid which is the actual beginning of the reaction mechanism.

Besides the geometry structure and electron effect, the physical chemistry properties of the real and simplified models calculated by conceptual density functional theory (CDFT)^26^ have been taken into consideration, and the results are listed in Table 1. Firstly, shown as the table, energies of HOMO and LUMO orbitals between Rh-sim and Rh-rea are almost the same, and the energy gap is in the difference of 0.02 eV. Also, there are almost no differences in characters of *μ*, *χ*, *η* and *ω* from Rh-rea to Rh-sim. These values suggest the physical chemical properties keep identical after simplification. In summary, with the slight difference in structures, electron effect of carbenoid and the physical chemistry properties, this computationally simplified catalyst model (Rh-sim) is rational and applied successfully in this system.

**Table 1:** The orbital energies of HOMO and LUMO, electronic chemical potential (*μ*), electronegativity (*χ*), chemical hardness (*η*), electrophilicity index (*ω*), and the energies are in eV

	Rh-sim	Rh-rea
HOMO	−6.12	−6.11
LUMO	−3.13	−3.14
Energy gap	2.99	2.97
*μ*	−4.63	−4.63
*χ*	4.63	4.63
*η*	2.99	2.97
*ω*	3.58	3.61

### Reaction mechanism

**Scheme 2:**
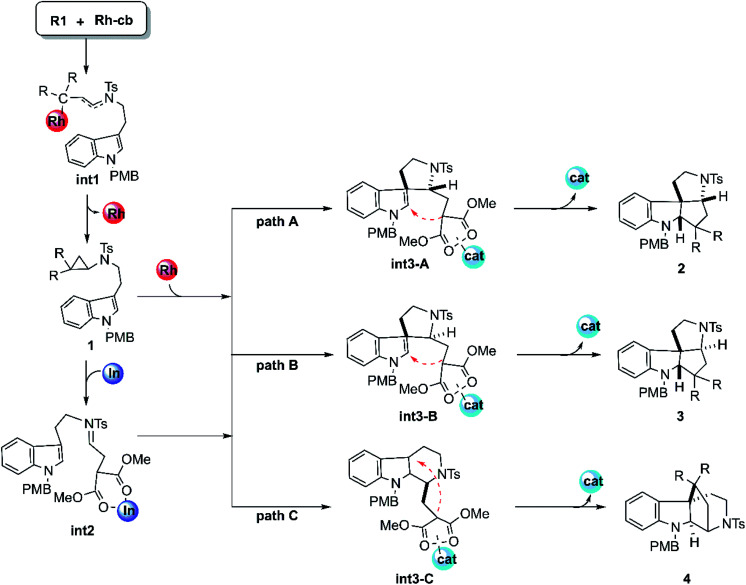
The supposed reaction mechanisms of the key steps for the cyclization reactions catalyzed by Rh_2_(esp)_2_ and InCl_3_.

The mechanism of this cyclization reaction catalyzed by Rh_2_(esp)_2_ and InCl_3_ is hypothesized and displayed in Scheme 2, according to the conjecture provided by the experimental authors^16^ and our previous study, after the calculational model confirmed. At first, the theoretical studies on the carbenoid conformation by the diazo compound reacting with dirhodium catalysts have been investigated repeatedly in literature. So, the study here starts with the carbenoid (Rh-cb) formed by the reaction between R2 and Rh_2_(esp)_2_. First step of the reaction is that the reactant R1 interacts with Rh-cb carbenoid to form intermediate int1 by addition step of carbene center and terminal vinyl group. Then int1 turns into the key cyclopropane intermediate 1 that was detected in experiment by a classical cyclopropanation step with the Rh_2_(esp)_2_ catalyst away. Noticeably, 1 can take directly the subsequent cyclization steps (the individual paths A, B, and C) when the catalyst is only Rh_2_(esp)_2_. As shown in Scheme 2, the three pathways aim at different products. It is worthy noting here that the way of Rh_2_(esp)_2_ interacted with 1 is through the Rh–O bond formed by the catalyst connected with one C=O group of ester group. While if the other catalyst InCl_3_ is added, the three-membered ring of compound 1 is broken to generate intermediate int2 with C=N bond. As shown, the int2 is formed by the InCl_3_ interacted with two C=O groups in O–In bonds. Then, int2 goes through three similar reaction pathways as above under the action of catalyst InCl_3_, except that the interaction sites of catalysts are different. For path A, five-membered ring intermediate int3-A can be obtained with the C–C bond forming. However, the distinct chirality C atom neighbor NTs group is different in path B. As polycyclic product formation, path A and path B are exactly alike through the bonding interaction between the C atom where two ester group connected and the C atom close to N-PMB group. By the way, products 2 and 3 are a pair of diastereoisomers. While, path C is more special, in which a six-membered ring intermediate int3-C is formed. Then the C atom connected to ester group interacts with tertiary C atom, producing endocyclic compound 4.

**Fig. 2:**
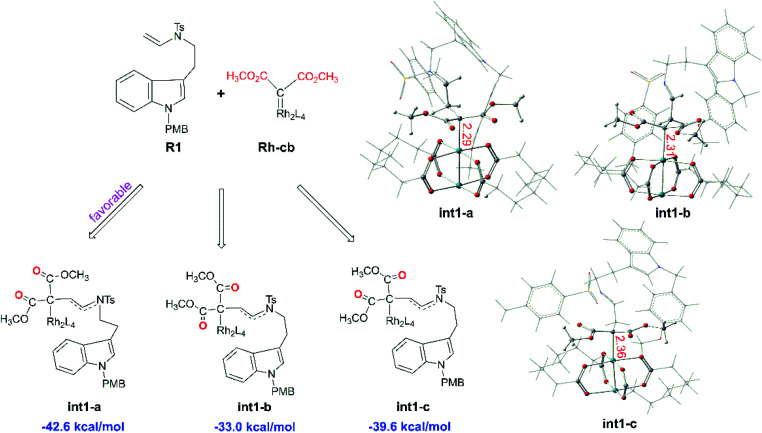
The structures of three intermediates int1-a, int1-b, and int1-c with C=O groups in different orientations as well as their free energies relative to the reactants R1 and Rh-cb (the red number describes bond lengths in Å).

**Fig. 3:**
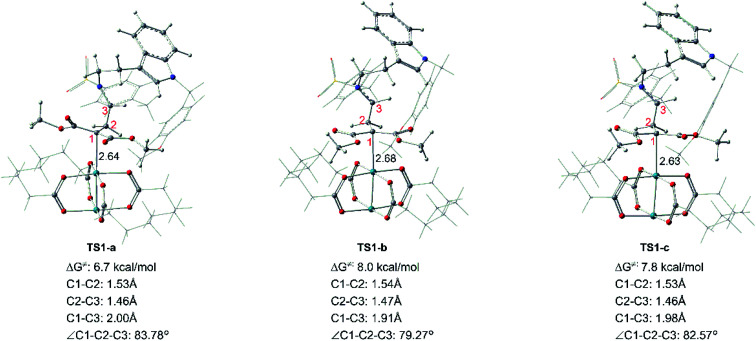
Transition states of int1-a, int1-b and int1-c to form the key cyclopropanation compound 1, the black number in structures means bond length with unit in Å.

Starting with R1 and Rh-cb, it is interesting that there is no free energy barrier for int1 formation. This step reacts quickly and generates the carbene addition intermediate int1 directly. Owing to the different orientation of the two ester groups in R1, there are three conformation isomers of intermediate: int1-a, int1-b and int1-c, respectively, as displayed in Fig. 2. All the intermediates are much stable compared with the initial reactants by releasing huge energy, among which int1-a is the most stable one with the free energy of −42.6 kcal mol^−1^ relative to the separated reactants R1 and Rh-cb. While, int1-b and int1-c have higher free energy values compared with int1-a by 9.6 and 3.0 kcal mol^−1^, respectively. The carbonyl O atoms of the two ester groups face outward for int1-a. By the way, we guess it is the stability of the int1s and huge difference in energy that make no energy barrier in this carbene addition step. Meanwhile, we find that the length of Rh–C bond is in accord with the energy of intermediates, and the most stable intermediate int1-a has the shortest bond length of 2.29 Å by comparing the structures of the three intermediates. In the other words, the shortest bond makes the interaction strong and keep more stable. What's more, the cyclopropanation step from the three intermediates to the key compound 1 observed in experiment has been compared, and the computational results are shown in Fig. 3. The C1 atom bonds with C3 to obtain 1, and it needs to overcome free energy barriers of 6.7 kcal mol^−1^, 8.0 kcal mol^−1^, and 7.8 kcal mol^−1^ for int1-a, int1-b and int1-c, respectively. Meanwhile, the bond lengths and bond angles of three-membered reaction centers are little different among three transition states. The results in Fig. 3 indicate that the lowest free energy barrier is 6.7 kcal mol^−1^ from int1-a to TS1-a. These computational results are corresponding to the intermediate int1-a which is the most stable intermediate aforementioned. Therefore, we adopt int1-a for the subsequent calculations. In fact, it is convenient for InCl_3_ catalyst to interact with the compound 1 with the outward C=O groups.

#### Reaction catalyzed by InCl_3_

**Fig. 4:**
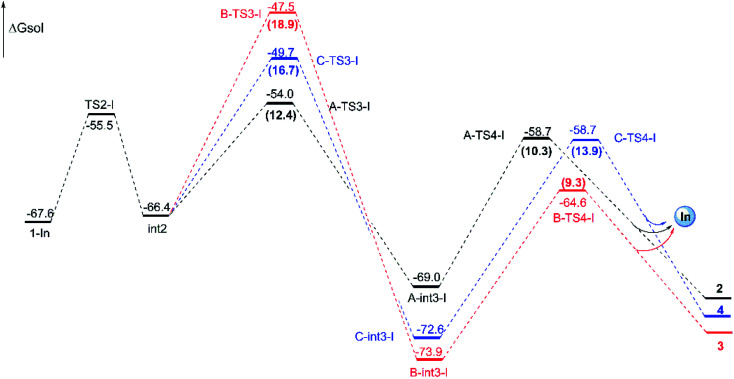
Free energy profiles for the three pathways catalyzed by InCl_3_ from 1-In to final products and energies are given in kcal mol^−1^. The numbers in brackets are the free energy barriers.

**Fig. 5:**
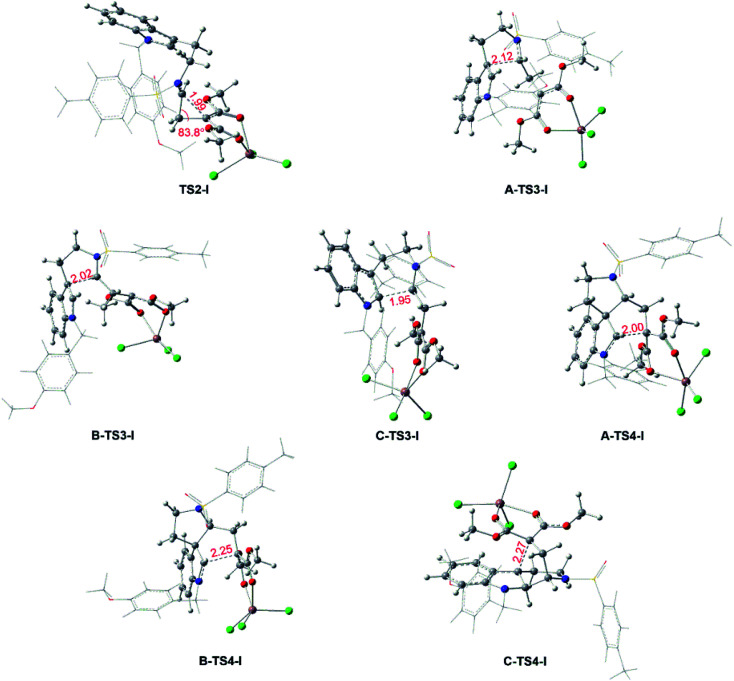
Key transition states for the InCl_3_-catalyzed pathways and bond lengths in red are in Å.

The experiment suggests that the product ratios are different with diverse catalysts.^16^ Therefore, three kinds of catalytic ways in next steps are explored (Scheme 2) after the formation of the key cyclopropanation intermediate 1 catalyzed by Rh_2_(esp)_2_. By the way, for the nomenclature of compounds shown in the free energy barrier profiles in this paper, the ‘I’ represents the way in InCl_3_ catalyst, the ‘R’ means the Rh-catalyst and the ‘n’ denotes the way without metal catalyst. The free energy profiles of InCl_3_-catalyzed way are displayed in Fig. 4 and the key transition states are shown in Fig. 5. Here, the way catalyzed by InCl_3_ starts with the complex intermediate 1-In formed by the interaction between 1 and InCl_3_*via* coordinate bonds. Firstly, the free energy barrier of 12.1 kcal mol^−1^ is expected to form the int2 through the transition state TS2-I with three-membered ring opening. In TS2-I, the length of broken bond is 1.99 Å and the corresponding angle is 83.8°. Then, the cycloaddition step needs the free energy barriers of 12.4, 18.9 and 16.7 kcal mol^−1^ for paths A, B, and C from int2 to int3-I, respectively. For the similar paths A and B, there is the difference of 0.10 Å in forming C–C bond between A-TS3-I and B-TS3-I. Next, the activation free energy of the TS4-I is calculated to be 10.3 kcal mol^−1^ for path A, 9.3 kcal mol^−1^ for path B and 13.9 kcal mol^−1^ for path C. There is 0.25 Å difference of the reaction center between similar transition states A-TS4-I and B-TS4-I. Reviewing the free energy barriers, all the steps can take place with the low free energy of activation. Comparing the three pathways, the cycloaddition steps *via*TS3-Is all are the rate-determining step with the highest free energy barrier in their respective pathways, which also confirms regio-selectivity to control whether the five-membered or six-membered ring intermediate formation and diastereoselectivity to decide the attack direction in this reaction catalyzed by InCl_3_. In the other words, the product selectivity is controlled by the cycloaddition step with TS3-I formation. Meanwhile, the calculated results in energies suggest path A is the most favorable way, the path C takes second place and path B is the last in dynamics among the three pathways. And these results are in good agreement with the experiment where product ratio is 2 > 4 > 3.

#### Reaction catalyzed by Rh_2_(esp)_2_

**Fig. 6:**
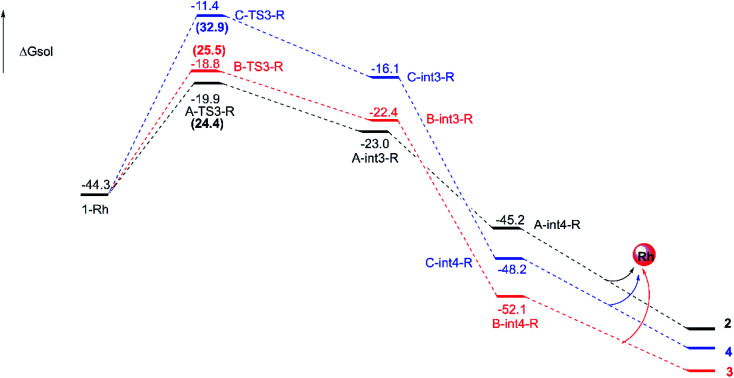
Free energy barrier profiles for the pathways catalyzed by Rh_2_(esp)_2_ from 1-Rh to final outcomes and the energy unit is kcal mol^−1^. The numbers in brackets are the free energy barriers.

**Fig. 7:**
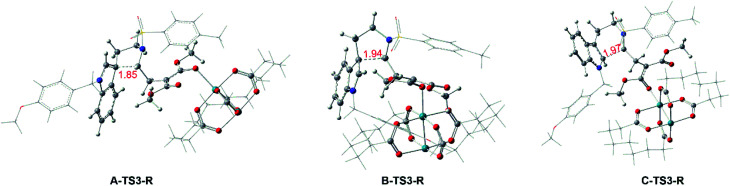
Geometry structures of transition states of Rh_2_(esp)_2_-catalyzed ways, and the numbers in red are bond length of the key atoms with unit in Å.

Interestingly, the similar steps catalyzed by Rh-catalyst from compound 1 are little different from the In-catalyzed way. And the free energy profiles are described in Fig. 6 and the geometrical structures of transition states are shown in Fig. 7. In this subsection, there is no int2 with C=N bond generation through the complex 1-Rh with three-membered ring opening. Actually, the 1-Rh transforms directly to int3-Rs *via* transition state TS3-Rs with the new C–C bond forming and the old C–C bond breaking in the three-membered ring simultaneously. For path A and path B, the free energy barriers of 24.4 and 25.5 kcal mol^−1^, respectively, for becoming five-membered ring intermediates have to be overcome. And 0.09 Å difference has been found in formed C–C bond of two transition states A-TS3-R and B-TS3-R. While, the six-membered ring intermediate C-int3-R is hard to obtain with the high free energy barrier of 32.9 kcal mol^−1^. Obviously, the cycloaddition steps from 1-Rh to int3-Rs in three pathways are all endothermal. Then the int3-Rs turn to be int4-Rs with exothermal in at least 20.0 kcal mol^−1^ where without the existence of transition state TS4-Rs, which is different from the way catalyzed by InCl_3_. We guess this is caused by the huge energy difference from int3-Rs to int4-Rs where the latter are more stable. Here, the ratio from path B and C is different with experimental ratio by the calculated results of free energy barriers. But we have tried our best to locate the transition states. Fortunately, the favored path A agrees with the experiment.

About the Rh-catalyzed way with full catalyst, we have also studied the reaction step from compound 1-Rh to int3 with the real Rh_2_(esp)_2_ catalyst. The corresponding geometric structures of key transition states as well as the free energy barriers ΔΔ*G*^≠^ and energy barriers ΔΔ*E*^≠^ relative to the most favored path A are displayed in Fig. S1 of ESI.† As shown, A-TS3-R-r is the most stable transition state in three pathways. Then, the secondary is B-TS3-R-r and the C-TS3-R-r is the last one. This order is the same with the pathways catalyzed by simplification model.

#### Reaction without catalyst

**Fig. 8:**
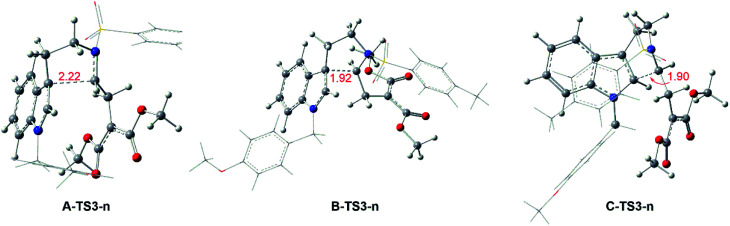
Geometrical structures of transition states for no-catalyzed way and the red values are the bond length of the key atoms in Å.

Keeping the role of catalysts in mind, the system from 1 to products with no catalyst is taken into consideration. In this case, the pathways are the same with Rh-catalyzed mechanism. From 1 to transition states TS3-ns, there need the free energy barriers of 33.8, 35.6 and 40.6 kcal mol^−1^ for path A, path B and path C in three pathways, and the geometry structures of key transition states TS3-ns are displayed in Fig. 8. And then it is no necessary to calculate the following step because of the high free energy barrier in cycloaddition step with TS3-ns formation.

### Explanations for the three-membered ring open step in 1 upon catalyst-dependency

As suggested by the calculated results of the free energy barriers, we find that the reaction system is relatively sensitive to the catalyst by comparing the three systems. Therefore, it is necessary to make comparison among the ways in different systems and gain a detailed insight into the importance of the metal catalysts. Firstly, the reaction catalyzed by InCl_3_ is the best way to get final outcomes with the lower free energy barrier under 20.0 kcal mol^−1^ seeing from the activation free energy. This result indicates that the InCl_3_ catalyst is more suitable for this reaction system, which agrees well with the experiment where the InCl_3_ catalyst makes the reaction time shorter.^16^ However, the reaction system without metal catalyst is hard to take place due to the such high free energy barrier. Therefore, it is sufficient to see the importance of the catalyst to the reaction. Moreover, seeing from the reaction step, compared with Rh-catalyzed and no-catalyzed systems, the mechanistic way in InCl_3_ has the step of three-membered ring open (from 1-In to int2), and we guess the int2 with C=N bond makes the C atom more active. So, the int2 occupies a prominent place in this reaction mechanism and more details are stated later.

**Fig. 9:**
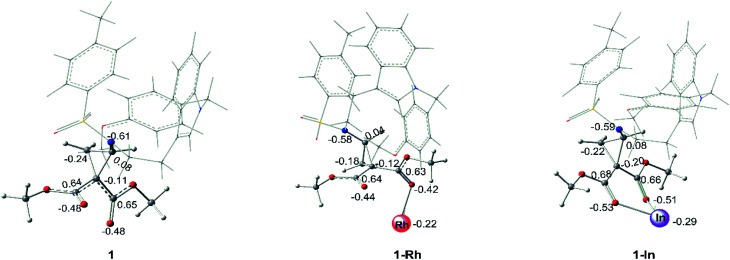
NBO analysis for 1, 1-Rh and 1-In (the values labeled in geometrical structures are charges).

As for the interaction between catalyst and the key compound 1, it is found that the C–C bond breakings in three-membered ring of no-catalyzed (1), Rh-catalyzed (1-Rh) and In-catalyzed (1-In) systems are different from each other. For In-catalyzed system, the three-membered ring opening step appears by the transition state TS2-I and it turns to intermediate int2 with the stable C=N bond, which participates in the following addition step. However, the alone opening step of the three-membered ring in systems 1 and 1-Rh does not exist and there are no transition states and intermediates like int2 to generate. While, 1 and 1-Rh go through directly the subsequent addition reaction. So, the initial interaction of the three systems has been compared, and the results of NBO analysis are displayed in Fig. 9. The optimized geometrical structures of 1-Rh and 1-In from Fig. 9, one can see that the interaction ways between the catalyst and 1 in two complexes are distinct. One of two ester groups of compound 1 interacts with Rh-catalyst with single coordination, while, there is a pair coordination in In-catalyzed system. Meanwhile, the length of Rh–O bond in 1-Rh is 2.29 Å, and the two In–O bonds of 1-In are 2.23 Å and 2.28 Å, seeing from the bond length. Due to the electronic effect of metal catalyst, the atomic charge of three-membered ring key atoms of 1 has differently changed in two systems. From Fig. 9, it is obvious that the catalysts act as an electron-withdrawing group when interacting with 1, the electrons transfer from the ring to catalyst and make catalyst become negative. The Rh_2_(esp)_2_ accepts 0.22e which is 0.07e lower than InCl_3_ compared the electron transfer capacity. That's to say, the catalyst Rh_2_(esp)_2_ has a weaker electron trapping capacity than InCl_3_ in this reaction system. Aiming at the three-membered ring broken, the charge difference of two C atoms(the C atom connected with two ester groups and the C atom neighbor to NTs group) is 0.19e, 0.16e and 0.28e in 1, 1-Rh and 1-In, respectively. It can be concluded that the larger charge difference in 1-In makes the transition state TS2 appears. In other words, the charge difference plays an important role in the C–C breaking step and the stable intermediate int2 formation.

As mentioned before, the frontier molecular orbital (FMO) analysis was carried out to understand the role of int2. And the results are depicted in Table 2. Because the reaction from 1 to final products is intramolecular, the energy gap between HOMO and LUMO orbitals in here was compared. As listed in Table 2, the energy gap of 1 with no catalyst is 4.58 eV which is the highest among all conditions. And this result also responds to its higher energy barrier. Meanwhile, there is 3.29 eV energy gap for 1-Rh, which is 0.24 eV higher than int2. Due to this 0.24 eV difference, the cycloaddition step in 1-Rh is harder than in int2. Also, there is 0.98 eV difference compared the energy gap between 1-In and int2. That's to say, the int2 formed by three-membered ring open step decreases the energy gap and makes the cycloaddition step easy to take place. Moreover, the most difference in int2 is the C=N bond, which is thought to be a very significant existence to make LUMO orbital lower.

**Table 2:** The FMO analysis of the four key intermediates, and the orbital energies are given in eV

Structures	HOMO	LUMO	Gap
1	−5.47	−0.89	4.58
1-Rh	−5.44	−2.15	3.29
1-In	−5.70	−1.67	4.03
int2	−6.11	−3.06	3.05

**Fig. 10:**
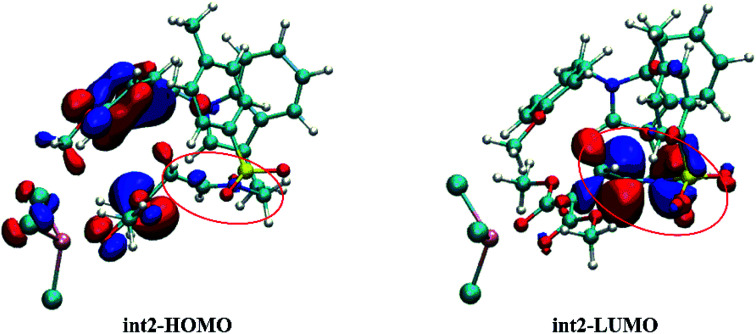
The HOMO and LUMO orbitals of intermediate int2 (the labeled part suggests the C=N bond site).

The FMO orbitals are depicted in Fig. 10 to draw a detailed picture of the int2. Results from Table 2 suggest that the energy gap reduced by int2 is mainly caused by the lower LUMO orbital. And combining with the orbital contribution in Fig. 10, it can be found that it is the C=N bond that plays an important role and contributes more to LUMO orbital. In addition, the more contribution of C=N bond to LUMO orbital makes its energy down and then shrinks the energy gap. And this calculated result is in accord with our assumption mentioned before.

### Origin of the selectivity

**Fig. 11:**
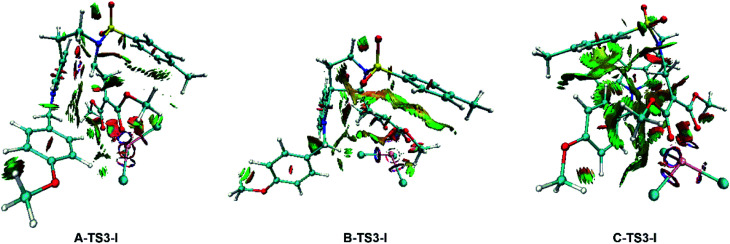
NCI plots for the transition state TS3-Is to analysis the selectivities. The red color in NCI plot means steric hindrance effect, the green represents van der Waals interaction and the blue one describes strong attraction interaction.

To gain deeper insight into the diastereo- and regio-selectivities of the title reaction, the non-covalent interaction (NCI) analysis was carried out by VMD^27^ and Multiwfn^28^ program packages, and the NCI results for TS3-Is are shown in Fig. 11. At first glance, the weak interaction of C-TS3-I is much larger than that in A-TS3-I and B-TS3-I. Obviously, the weak interaction in this part is repulsive interaction and makes the energy higher. The reaction to form six-membered ring transition state is harder than the five-membered owing to the difference in NCI plots. In agreement to this, the five-membered ring product 2 is principal, and this is consistent with that experiment observed. However, the result disagrees with the highest free energy barrier of B-TS3-I with the little NCI difference between A-TS3-I and B-TS3-I. In summary, it is the weak interaction that only control the regio-selectivity of the reaction catalyzed by InCl_3_.

Although the weak interaction in B-TS3-I is smaller than that in C-TS3-I (see Fig. 11), the activation free energy barrier of B-TS3-I is the highest among three pathways within 18.9 kcal mol^−1^, and the productivity of 3 is also the last. Besides, the NCI analysis only indicates the origin of regio-selectivity, and the diastereo-selectivity is unclear. So, in this part, NBO second-order perturbation analysis was calculated to compare A-TS3-I and B-TS3-I. The *E*(2) value includes two parts as such electron donor and acceptor. And the *E*(2) value shows the electron delocalization degree, and it is easy to get the each bond contributing to *E*(2) value, where larger *E*(2) value is, bond interaction is more strong. Also the stability of the structure can be described by total *E*(2) value in a degree. As well, the *E*(2) value contributed by the bonding atoms (C23–C27) are displayed in Table 3. It is noticeable that the main values (>10) were adopted. Seeing from the *E*(2) value in Table 3, the total value of A-TS3-I is 28.4 kcal mol^−1^ higher than the one of B-TS3-I, which indicates the electron delocalization degree of C23–C27 bond in A-TS3-I is larger than that in B-TS3-I. In other words, the structure of transition state A-TS3-I is more stable. Here, the computational results by NBO analysis are according with the free energy barrier in Fig. 4. Taken together, it can be concluded that the electronic effect makes the A-TS3-I in lower energy barrier and also decides the diastero-selectivity.

**Table 3:** NBO second-order perturbation analysis for transition states A-TS3-I and B-TS3-I, and the energies are given in kcal mol^−1^

	*E*(2)	Donor	Acceptor
A-TS3-I	12.2	BD(2) C16–C17	BD*(1) C23–C47
	13.7	BD(1) C23–C47	BD*(2) C16–C17
	39.6	BD(1) C23–C47	BD*(2) C22–N29
	26.4	LP(1) N46	BD*(1) C23–C47
	20.5	BD*(2) C22–N29	BD*(1) C23–C47
	56.1	BD*(1) C23–C47	BD*(2) C16–C17
**Total**	**168.5**		
B-TS3-I	12.9	BD(1) C23–C47	BD*(2) C16–C17
	31.8	BD(1) C23–C47	BD*(2) C22–N29
	14.0	BD*(2) C22–N29	BD*(1) C23–C47
	61.7	BD*(2) C16–C17	BD*(1) C23–C47
	19.7	LP(1) N46	BD*(1) C23–C47
**Total**	**140.1**		

**Fig. 12:**
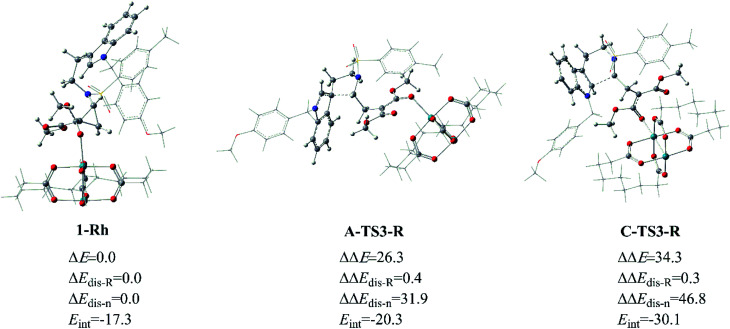
The distortion/interaction energy analysis for transition states A-TS3-R and C-TS3-R, and energies are given in kcal mol^−1^.

As for the way catalyzed by Rh_2_(esp)_2_, there is only regio-selectivity observed in experiment. Firstly, the free energy of A-TS3-R is 8.5 kcal mol^−1^ lower than C-TS3-R, which suggests the five-membered ring transition state is more stable than that six-membered as mentioned in Fig. 6. So, we thought it is the better stability of A-TS3-R that decides the regio-selectivity to form major product 2. In this respect, the transition states A-TS3-R and C-TS3-R were compared carefully. Of the same reason in InCl_3_ catalyst way, the NCI analysis for the two transition states were considered and the results are given in Fig. S2.† Seeing from the NCI plots, it can be found the weak interaction and steric hindrance effect are little difference. Therefore, the distortion/interaction energy analysis was adopted to make the energy contribution clear and the computational results are displayed in Fig. 12. In this part, the distortion energy was relative to the complex 1-Rh and the transition state was divided into two fragments, one only including Rh_2_(esp)_2_ abbreviated to ‘R’ and the one without catalyst named as ‘n’. As suggested in Fig. 6, the free energy difference between two transition states is 8.5 kcal mol^−1^ where the A-TS3-R is lower.

Well, the distortion energies of the Rh-cata part in two transition states are almost the same and small, and this result indicates that the distortion of Rh-cata is very slight during the formation of transition state from the results shown in Fig. 12. Attentively, the distortion difference in the part without Rh-cata is so distinct. By contrast, there are nearly 15.0 kcal mol^−1^ difference in distortion energy of ‘n’ part. And then the absolute value of interaction energy of C-TS3-R is 9.8 kcal mol^−1^ higher than that of A-TS3-R, which suggests that the transition state C-TS3-R could release more energy when the distorted Rh-cata interacts with distorted ‘n’ part. Taken together, the largest difference between the two transition states is mainly contributed by the distortion of ‘n’ part. So, it can be surmised that distortion energy of ‘n’ part to form transition state C-TS3-R part plays the important role in the regio-selectivity catalyzed by Rh_2_(esp)_2_.

## Conclusion

This work adopts the DFT study to explore the cyclization reaction catalyzed by Rh_2_(esp)_2_ and InCl_3_ to form polycyclic indolines. At first, the simplification of the Rh_2_(esp)_2_ model is successful by the little difference among real computational model and crystal both in structure and character. The reaction mechanistic details of the three pathways to get products 2, 3 and 4 were detailed reported in this paper. Comparing the ways catalyzed by Rh_2_(esp)_2_ and InCl_3_, the latter is more favorable with the key intermediate int2 formed. NBO analysis for 1, 1-Rh and 1-In shows the larger charge difference in 1-In which can help three-membered ring open as well. The generation of int2 with C=N bond formed by ring open of three-membered cyclopropane decreases the HOMO–LUMO energy gap and makes the cyclization reaction easy to take place. With the higher energy barrier in TS3-Is, the rate-determining step, regio- and diastero-selectivity step are controlled by the intramolecular cycloaddition step. Also, the rate-determining step and regio-selectivity of the pathways catalyzed by Rh_2_(esp)_2_ (TS3-Rs formation) is the same with the InCl_3_-catalyzed way. The regio-selectivity in In-catalyzed way is decided by the weak interaction (repulsive interaction). However, the larger distortion energy to form transition state C-TS3-R affects the regio-selectivity in Rh-catalyzed way. As for the diasteroselectivity observed in experiment, it is the larger electron delocalization degree that makes transition state A-TS3-I more stable and decides the diasteroselectivity consequently, suggested by the results of NBO second-order perturbation analysis. This theoretical study may reveal the importance for the catalyst to chemical reaction and understand their roles.

## Conflicts of interest

There are no conflicts to declare.

## Supplementary Material

RA-011-D1RA01632F-s001
