# Supplementary material for: Elucidating the mechanism and origins of selectivity on catalyst-dependent cyclization reactions to form polycyclic indolines from a theoretical study
Source: RSC Adv. 2021 Jun 9;11(34):20622–34. doi: 10.1039/d1ra01632f (PMC9033988; doi:10.1039/d1ra01632f)
Supplement: RA-011-D1RA01632F-s001 [file RA-011-D1RA01632F-s001.pdf]

Electronic Supplementary Material (ESI) for RSC Advances.
This journal is © The Royal Society of Chemistry 2021

Supporting Information

Elucidating the mechanism and origins of selectivity on catalyst-dependent cyclization reactions to form polycyclic indulines from a theoretical study

Yan Zhang, Yongsheng Yang, Ying Xue*

College of Chemistry,
Key Lab of Green Chemistry and Technology in Ministry of Education,
Sichuan University, Chengdu 610064,
People's Republic of China

Constants

Figure S1. Geometric structures of transition states as well as the free energy barriers $\Delta\Delta G^{\neq}$ and energy barriers $\Delta\Delta E^{\neq}$ relative to the most favored path A from **1-Rh** to **int3** with real $Rh_2(esp)_2$ catalyst (energies are given in kcal/mol) s2

Figure S2. NCI plots for the transition states **A-TS3-R** and **C-TS3-R**……………..……… s3

Cartesian coordinates (Å) for all ground state and transition state geometries......................s4

Corresponding author. Ying Xue, e-mail: yxue@scu.edu.cn

Tel: +86 28 85418330.

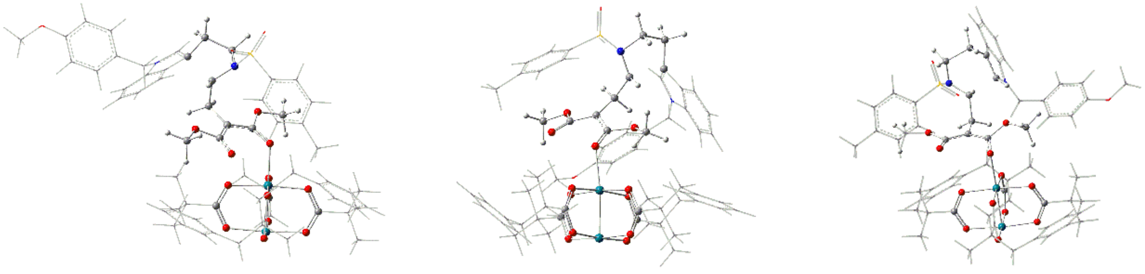

A-TS3-R-r $\Delta\Delta G^{\neq}$=0.0 $\Delta\Delta E^{\neq}$=0.0

B-TS3-R-r $\Delta\Delta G^{\neq}$=8.5 $\Delta\Delta E^{\neq}$=4.7

C-TS3-R-r $\Delta\Delta G^{\neq}$=11.0 $\Delta\Delta E^{\neq}$=10.6

Figure S1. Geometric structures of transition states as well as the free energy barriers $\Delta\Delta G^{\neq}$ and energy barriers $\Delta\Delta E^{\neq}$ relative to the most favored path A from **1-Rh** to **int3** with real $Rh_2(esp)_2$ catalyst (energies are given in kcal/mol).

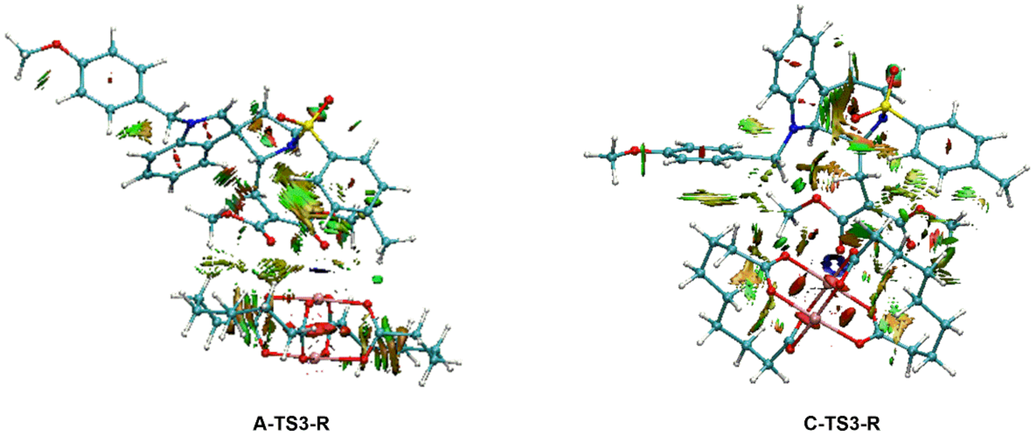

Figure S2. NCI plots for the transition states **A-TS3-R** and **C-TS3-R**.

Cartesian coordinates (Å) for all ground state and transition state geometries

1

0 1

C	2.03078800	-2.17501700	-0.17795500
C	1.21593400	-3.03157400	-1.09666900
O	1.65331800	-3.94021800	-1.76643100
C	3.51081800	-2.23513000	-0.46039400
O	3.99196900	-1.95468900	-1.53766300
O	-0.09571100	-2.69129200	-1.05875600
O	4.22689600	-2.60053400	0.61360300
C	-0.97074300	-3.51337300	-1.85211900
H	-0.66127900	-3.49740000	-2.90014600
H	-0.94733900	-4.54650600	-1.49556600
H	-1.96238200	-3.08264800	-1.72787800
C	5.65509800	-2.58296200	0.43106400
H	6.07558400	-2.89871900	1.38492500
H	5.94509200	-3.26734700	-0.36977500
H	5.99188600	-1.57462200	0.17737200
C	-1.10664600	2.70850300	-1.86931800
C	0.31782700	2.67114300	-1.89519200
C	1.03581900	3.86399500	-1.70302200
C	0.33889500	5.04354400	-1.47050500
C	-1.07102500	5.05897600	-1.44423600
C	-1.81037700	3.89898400	-1.65296400
C	-0.46293500	0.58687000	-2.22199700
C	0.70352200	1.29945900	-2.10866200
H	2.12088200	3.86295200	-1.72516300
H	0.88424200	5.96860500	-1.31152900
H	-1.59082400	5.99593500	-1.26796800
H	-2.89554200	3.92599800	-1.64458900
H	-0.59930100	-0.47699600	-2.34971900
N	-1.56252900	1.41746700	-2.07311900
C	-4.10123800	-1.21720700	-1.51817800
C	-2.49231500	-0.37179500	0.05298000
C	-4.36061900	-2.31183900	-0.68522100
H	-4.63782300	-1.13777000	-2.46066000
C	-2.72701000	-1.45664800	0.88410300
H	-1.76252300	0.37361500	0.34703800
C	-3.66412100	-2.43668100	0.52294600
H	-5.08675800	-3.05410000	-0.99327200
H	-2.19280800	-1.56403700	1.82110800
O	-3.81290500	-3.46353900	1.40752000

C	2.09062300	0.73273200	-2.15105000
H	2.06132700	-0.33354500	-2.39236100
H	2.66966500	1.20204100	-2.95810000
C	2.92563500	0.94068500	-0.87594300
H	3.92400500	0.53243200	-1.05514500
H	3.03762800	2.00273200	-0.66209400
N	2.37831300	0.30145200	0.34972300
C	1.47294300	-0.80990800	0.25580400
C	1.53794400	-1.93766100	1.23560300
H	0.49249900	-0.58371900	-0.14712900
H	0.59290700	-2.39449500	1.50744400
H	2.29939300	-1.89860800	2.00299500
S	2.32501100	1.18521900	1.79621800
O	2.98747800	2.46525100	1.52544300
O	2.79583500	0.30971500	2.87553100
C	0.58494500	1.49831100	2.10989500
C	-0.03802800	2.58587100	1.49863300
C	-0.12120800	0.64684400	2.96309700
C	-1.39557700	2.80326500	1.73035200
H	0.52520700	3.25183000	0.85678000
C	-1.47443700	0.88381400	3.18703100
H	0.39634200	-0.17239700	3.44872800
C	-2.13500000	1.95267600	2.56245300
H	-1.88175500	3.63975800	1.23886900
H	-2.03060000	0.22588200	3.84934800
C	-3.62113000	2.13647800	2.73667000
H	-3.93404600	3.15554300	2.49374600
H	-4.16189100	1.45154000	2.07143800
H	-3.93813400	1.91412900	3.76014700
C	-4.72983500	-4.50022300	1.08808700
H	-4.45236400	-5.01332000	0.15775700
H	-4.68359000	-5.20910800	1.91576300
H	-5.75579800	-4.12051300	0.99509200
C	-3.17497100	-0.23607200	-1.16592700
C	-2.94593200	0.96746100	-2.06239700
H	-3.27879300	0.73978700	-3.08349600
H	-3.55467700	1.80845900	-1.71545300

R1

0 1

C	-1.49944600	2.52402600	-0.19771700
C	-2.60629000	1.62778500	-0.18358900
C	-3.25875900	1.33121300	-1.39273200
C	-2.78558600	1.89801600	-2.57014000

C	-1.67180500	2.76279200	-2.56527100
C	-1.01528900	3.08851500	-1.38253300
C	-1.80567800	1.82639000	1.90777500
C	-2.76770100	1.17995500	1.17646400
H	-4.10201000	0.64877100	-1.41335100
H	-3.27077900	1.66171300	-3.51183700
H	-1.31671000	3.17899700	-3.50315800
H	-0.14968700	3.74298200	-1.38358000
H	-1.59087900	1.75802100	2.96577800
N	-1.04386400	2.65771000	1.10177700
C	2.57795000	2.71742500	0.59854500
C	1.35533400	0.93684100	1.64566000
C	3.65996400	1.86466000	0.40295300
H	2.64530900	3.74720000	0.25519700
C	2.42673000	0.06842000	1.45735200
H	0.45417100	0.54051500	2.09957400
C	3.59088000	0.53518900	0.83776000
H	4.56585800	2.20464000	-0.08743100
H	2.32135600	-0.96335400	1.76756900
O	4.69654900	-0.23068800	0.59018100
C	-3.68627100	0.11528900	1.68737700
H	-3.58657000	0.04322200	2.77635800
H	-4.73931600	0.36387300	1.50012600
C	-3.45186000	-1.26688700	1.03104800
H	-4.01657200	-2.03137600	1.57984000
H	-3.82967100	-1.24899500	0.01099100
N	-2.02748300	-1.65583000	0.94065300
C	-1.26120500	-1.69623900	2.11641800
C	-0.16616600	-2.41293100	2.40134300
H	-1.63333500	-0.98946300	2.85134200
H	0.32143900	-2.25172900	3.35557200
H	0.24863800	-3.16020700	1.74001800
S	-1.55188800	-2.56672900	-0.43488200
O	-2.58187300	-2.30326800	-1.44521600
O	-1.24100700	-3.94601400	-0.03993100
C	-0.02528400	-1.79205400	-0.95911200
C	-0.04681900	-0.47180400	-1.40480700
C	1.13487600	-2.56467100	-1.04071800
C	1.12077600	0.08722900	-1.91687400
H	-0.95823700	0.10731000	-1.36153200
C	2.28692300	-1.99209700	-1.57338400
H	1.11763900	-3.59667300	-0.70895400
C	2.30007500	-0.66039300	-2.01660700
H	1.10443300	1.12190300	-2.24318000

H	3.19076800	−2.58987600	−1.65708500
C	3.56177700	−0.06389000	−2.58827300
H	3.86202200	−0.58793600	−3.50298900
H	3.42846800	0.99297900	−2.83114700
H	4.38382600	−0.14665600	−1.87075800
C	4.71980300	−1.55516200	1.10944900
H	4.62119600	−1.55789000	2.20255900
H	3.92542400	−2.17256200	0.67487700
H	5.69108600	−1.96836100	0.83351300
C	1.41209400	2.27041000	1.23147100
C	0.24709400	3.21267000	1.48548800
H	0.18089700	3.46790300	2.54944600
H	0.39481600	4.15514100	0.94898600

1-Rh

0 1

C	−0.61309200	1.08612600	−0.67213700
C	−0.11683700	0.14322100	−1.72427300
O	0.86834600	−0.57831200	−1.65774200
C	−0.15313400	2.50043100	−0.86329700
O	−0.38022200	3.41927300	−0.10757500
O	−0.87390000	0.17696800	−2.82828100
O	0.57762500	2.61106000	−1.99135300
C	−0.36259900	−0.54142700	−3.97115300
H	−0.27237300	−1.60647900	−3.74862500
H	−1.08790000	−0.37081900	−4.76581900
H	0.62009100	−0.15013200	−4.24345300
C	1.20833200	3.88582300	−2.19823700
H	1.83017200	3.76369900	−3.08416400
H	0.45663500	4.66431200	−2.35317100
H	1.82172400	4.14572600	−1.33405300
C	−6.05942700	−1.30108800	−2.14112200
C	−6.29986700	0.06155100	−1.80572200
C	−7.59076400	0.45700900	−1.42541600
C	−8.59891100	−0.49858200	−1.36505700
C	−8.33651500	−1.84989400	−1.66925000
C	−7.06708900	−2.26927400	−2.05870600
C	−4.13936000	−0.16038600	−2.38537000
C	−5.04855700	0.76601500	−1.94514400
H	−7.79430000	1.49187500	−1.16809100
H	−9.60311500	−0.20413600	−1.07480800
H	−9.14066000	−2.57747500	−1.60747600
H	−6.87749000	−3.31047400	−2.30145600
H	−3.08960300	−0.04279000	−2.60529900

N	-4.73053800	-1.41231000	-2.52651900
C	-3.86284600	-2.79072000	0.01232200
C	-1.77404200	-2.76550500	-1.17210200
C	-3.18406900	-2.69814800	1.22757300
H	-4.94915700	-2.80809900	0.01068900
C	-1.07570500	-2.67715900	0.02964500
H	-1.21262200	-2.77655200	-2.10191300
C	-1.78623200	-2.59665600	1.23626200
H	-3.74676500	-2.66559400	2.15301500
H	0.00506600	-2.58595800	0.05269000
O	-1.04456300	-2.38098900	2.35996300
C	-4.81929900	2.21963300	-1.63790400
H	-5.03240500	2.84703600	-2.51587100
H	-5.53609200	2.53384600	-0.87179500
C	-3.40076000	2.57743000	-1.16072600
H	-2.67275800	2.33296500	-1.93906900
H	-3.33541400	3.65285400	-0.98007600
N	-2.93555500	1.88083200	0.05518100
C	-2.00235300	0.80071500	-0.06118100
C	-0.76933900	0.67271700	0.76904800
H	-2.42797300	-0.13843300	-0.40645600
H	-0.43417300	-0.31555000	1.05529500
H	-0.55813100	1.46332200	1.47351100
S	-3.56165800	2.34950700	1.53946900
O	-3.82765300	3.78883300	1.46608200
O	-2.70270400	1.77545300	2.58182300
C	-5.15137200	1.53009600	1.67316500
C	-6.29297500	2.27220600	1.96571700
C	-5.20953100	0.14226700	1.53718400
C	-7.50861800	1.60403300	2.13070000
H	-6.22227300	3.35029100	2.05958500
C	-6.42646300	-0.50535700	1.70178500
H	-4.31815700	-0.41910500	1.28964200
C	-7.59395800	0.21255400	2.00700200
H	-8.40407500	2.17631800	2.35787000
H	-6.48204700	-1.58318200	1.57442700
C	-8.90230600	-0.51565400	2.18572800
H	-9.74063100	0.17872100	2.28875900
H	-9.09895800	-1.16412900	1.32623700
H	-8.88033600	-1.14940900	3.08014600
C	-1.70619700	-1.84228000	3.50513500
H	-2.38691700	-2.57113200	3.96244200
H	-0.91558100	-1.60334300	4.21792700
H	-2.25368200	-0.92600700	3.25171700

Rh	4.77605300	-0.12845800	0.89278300
O	5.45590700	1.17823500	-0.57593300
O	3.91810600	1.45179700	1.93507000
O	3.51702900	1.00482600	-1.74269600
O	2.03558300	1.31330500	0.68603100
C	4.67912600	1.48685900	-1.53268100
C	5.16945800	2.52900100	-2.52593400
C	5.86032200	3.74851600	-1.88780400
H	6.66267900	3.38630400	-1.23548500
H	6.33778800	4.32127700	-2.69189500
C	3.17061200	4.24706200	2.42085000
H	2.71182100	4.98668000	3.08719100
H	4.07551400	3.88354000	2.92122000
C	2.18395700	3.07402500	2.27346300
C	2.76528600	1.85187300	1.58489800
Rh	2.73066300	-0.32005400	-0.36416200
O	2.02469200	-1.60200100	1.10776100
O	3.58640600	-1.91378200	-1.38209400
O	3.97407400	-1.45906100	2.25663700
O	5.50611900	-1.72537800	-0.18766600
C	2.80226300	-1.91839900	2.06848900
C	2.28725800	-2.93037300	3.07701800
C	1.69104000	-4.21530200	2.46979700
H	0.86418800	-3.93290500	1.81396000
H	1.24842500	-4.78780100	3.29399700
C	4.34490500	-4.69432800	-1.85505700
H	4.78991600	-5.43915300	-2.52587200
H	3.42492900	-4.34205800	-2.33590500
C	5.32418000	-3.51006100	-1.75329400
C	4.75925900	-2.28461700	-1.05387000
H	3.10589200	-3.15889000	3.76504000
H	1.49927700	-2.42179800	3.64553900
H	1.86437300	2.74504300	3.27052700
H	1.28766900	3.37573900	1.72454700
H	5.88175600	2.01733000	-3.18561600
H	4.31748200	2.83102900	-3.14243400
H	6.24145500	-3.79728400	-1.23151900
H	5.60798300	-3.18999700	-2.76395000
C	2.67349400	-5.11589700	1.70043600
H	3.48910700	-5.43796500	2.36332100
H	2.13686100	-6.02913200	1.40814700
C	3.25065400	-4.44198900	0.45064100
H	2.44303700	-3.93746900	-0.09118100
H	3.93638100	-3.65354700	0.77157000

C	3.99294900	-5.37007100	-0.51675500
H	3.36813800	-6.24832800	-0.73022800
H	4.90807900	-5.75232000	-0.04257400
C	3.55982400	4.93647200	1.09981600
H	2.65794800	5.34100900	0.61802600
H	4.19522400	5.80104300	1.33527400
C	4.30474700	4.00684200	0.13517300
H	5.08294800	3.46934800	0.68646300
H	3.60871800	3.23922900	-0.21552200
C	4.93586900	4.68379800	-1.08572300
H	5.52521300	5.55047100	-0.75761200
H	4.15038700	5.08252200	-1.74480000
C	-3.17494100	-2.78696900	-1.20493900
C	-3.94246200	-2.64741800	-2.50820300
H	-3.24906500	-2.63480700	-3.35617400
H	-4.62952400	-3.48212600	-2.67146300

2

0 1

C	0.39591600	1.58053000	-0.28671400
C	0.61851100	2.77595500	-1.22474200
O	1.68374100	3.32694400	-1.39449300
C	-0.13870200	2.17487800	1.02134000
O	-0.78128400	3.20180300	1.09739800
O	-0.52263000	3.16426500	-1.82504400
O	0.14579100	1.39836200	2.07828500
C	-0.42174500	4.36147300	-2.61797000
H	0.31952400	4.23552700	-3.41076000
H	-1.41487900	4.51995400	-3.03695400
H	-0.13011900	5.20479300	-1.98743500
C	-0.43793500	1.80051700	3.32912100
H	-0.05216900	1.09825700	4.06666800
H	-0.15527400	2.82680600	3.57379400
H	-1.52719600	1.72920100	3.27516700
C	-1.47150900	-0.79141600	0.79767700
C	-0.26765200	-1.38826600	0.37217700
C	0.30846700	-2.40024100	1.12519500
C	-0.32370300	-2.82016600	2.30662300
C	-1.52837800	-2.23764800	2.70338400
C	-2.12356600	-1.21360500	1.95601100
C	-0.69769900	0.53737200	-0.91814200
C	0.10708100	-0.77423500	-0.97756400
H	1.24897900	-2.84158000	0.81446800
H	0.12416300	-3.60510500	2.90794100

H	-2.01681100	-2.57931000	3.61184300
H	-3.06162100	-0.76438600	2.26240900
H	-1.02479500	0.91157000	-1.88709000
N	-1.83635100	0.22917700	-0.07660200
C	-5.38342900	0.85135700	0.78502500
C	-4.50761400	-0.58458300	-0.92682800
C	-6.64612300	0.26598900	0.65160600
H	-5.24530300	1.64145000	1.51980600
C	-5.75198700	-1.18146900	-1.07094400
H	-3.66985500	-0.92720000	-1.52663800
C	-6.83326300	-0.75765000	-0.28351100
H	-7.45982500	0.60672200	1.28026300
H	-5.91640900	-1.98328800	-1.78316100
O	-8.01381700	-1.40955300	-0.50100600
C	0.01144800	-1.81345000	-2.10969700
H	-0.80569400	-2.52608400	-1.98189900
H	-0.11188800	-1.31012600	-3.07443800
C	1.40342600	-2.50828900	-2.03339100
H	1.36270000	-3.46797400	-1.51554800
H	1.82191600	-2.67956200	-3.02904500
N	2.26431400	-1.57097100	-1.21685300
C	1.53973000	-0.29588400	-1.17229300
C	1.72766100	0.77554500	-0.11203500
H	1.60799400	0.19663900	-2.15852900
H	2.58955700	1.42139000	-0.27336600
H	1.78517400	0.33113800	0.87991100
S	3.88762200	-1.51093000	-1.69690000
O	4.07703200	-0.56672900	-2.81039500
O	4.30635100	-2.90970700	-1.83822500
C	4.62074700	-0.79168500	-0.23275100
C	5.26488700	0.43959900	-0.32423900
C	4.52330700	-1.47255000	0.98289000
C	5.81597300	0.99931600	0.82847500
H	5.31272500	0.94876800	-1.28032700
C	5.07455700	-0.89709000	2.12195000
H	4.01368100	-2.42842900	1.02991900
C	5.72869600	0.34449900	2.06301300
H	6.31377400	1.96286500	0.76709000
H	4.99573200	-1.41449900	3.07425800
C	6.34242800	0.94195100	3.30537200
H	5.69868400	0.79435500	4.17816200
H	7.30544300	0.46725900	3.52924400
H	6.52189100	2.01421700	3.19038000
C	-9.14177900	-1.02689900	0.27011300

H	-9.40904600	0.02549900	0.10515300
H	-9.96466100	-1.66142500	-0.06254600
H	-8.97401200	-1.18812900	1.34344900
C	-4.30333200	0.44587400	0.00173400
C	-2.95396700	1.12923000	0.13903300
H	-2.85233200	1.93730200	-0.59313100
H	-2.89192500	1.61400600	1.12387400

R2

0 1

C	0.00000400	0.19346700	0.00004700
C	-1.30320500	-0.49713300	-0.00003000
O	-1.45696200	-1.69539600	-0.00026600
C	1.30322000	-0.49711200	0.00011500
O	1.45697700	-1.69537600	0.00044600
N	-0.00002000	1.51286700	0.00007200
N	-0.00004200	2.64473700	0.00008600
O	-2.31588300	0.40886700	0.00015400
O	2.31589600	0.40888300	-0.00022700
C	-3.63607400	-0.16322200	-0.00010100
H	-3.78691400	-0.78161800	0.88834400
H	-4.32090300	0.68431000	0.00039500
H	-3.78700500	-0.78062100	-0.88922700
C	3.63608600	-0.16320700	-0.00018700
H	4.32091500	0.68432600	0.00008200
H	3.78685600	-0.78131400	0.88847200
H	3.78708700	-0.78089300	-0.88910000

3

0 1

C	1.42175100	-1.91990400	-0.54368500
C	0.80490100	-2.16856100	-1.92850400
O	0.84665100	-1.37461600	-2.84297000
C	2.89114100	-2.35329500	-0.66223400
O	3.48801200	-2.50382300	-1.70471900
O	0.24532900	-3.38318700	-2.01272900
O	3.46048200	-2.49964100	0.55121600
C	-0.44353200	-3.65382900	-3.24567800
H	0.25302200	-3.62226900	-4.08719100
H	-0.87107400	-4.64902300	-3.12968900
H	-1.23006200	-2.91119700	-3.39795200
C	4.88661300	-2.66817400	0.53984700
H	5.18285400	-2.72659900	1.58665100
H	5.16434200	-3.57834600	0.00265700

H	5.35833800	-1.80902900	0.05642600
C	3.11360400	0.51114200	1.03636300
C	2.07841100	0.19890800	1.93642100
C	2.26981800	0.32688700	3.30405300
C	3.50459000	0.78377700	3.78623000
C	4.52021600	1.11841400	2.88677800
C	4.34119700	0.98637700	1.50540100
C	1.40615300	-0.37056800	-0.26941500
C	0.83025200	-0.14777000	1.15198400
H	1.46564600	0.08706000	3.99587600
H	3.66859900	0.88429800	4.85449700
H	5.47291100	1.48018600	3.26356700
H	5.14390500	1.23320900	0.81758200
H	0.77952700	0.08463100	-1.02905400
N	2.74669300	0.22908500	-0.28211000
C	2.19891500	3.36518900	-0.56608300
C	0.99003900	2.26569300	-2.33071600
C	1.23448400	4.37401200	-0.51538200
H	3.03769400	3.40198400	0.12363600
C	0.01953900	3.26033900	-2.29178900
H	0.87810400	1.43132600	-3.01839100
C	0.13474000	4.31986000	-1.38159200
H	1.34898300	5.18469000	0.19400600
H	-0.84019000	3.23610700	-2.95309000
O	-0.87395100	5.24298500	-1.41703300
C	-0.25986600	0.96349800	1.24075900
H	0.08129200	1.82670500	1.81586200
H	-0.53027200	1.30976000	0.24036900
C	-1.44841900	0.25297300	1.89961700
H	-1.33927700	0.23611300	2.99063000
H	-2.42034000	0.69330900	1.67707100
N	-1.37971600	-1.16693500	1.46327200
C	0.09574200	-1.46716300	1.53448500
C	0.68338200	-2.58752200	0.65689200
H	0.25397200	-1.69829700	2.59279400
H	-0.06066900	-3.30963200	0.33133400
H	1.41237500	-3.12187200	1.26437600
S	-2.18091800	-1.49931800	-0.01221000
O	-2.27433900	-2.95905500	-0.13357700
O	-1.61378900	-0.72133000	-1.13263700
C	-3.82098300	-0.86308000	0.32186300
C	-4.26630600	0.26949100	-0.35682000
C	-4.64531800	-1.54674900	1.21837100
C	-5.56146300	0.72997200	-0.12038700

H	-3.60595900	0.76829600	-1.05791300
C	-5.93205400	-1.06971100	1.44323200
H	-4.28046400	-2.43772500	1.71727400
C	-6.40872200	0.07381800	0.78126900
H	-5.91885200	1.60976600	-0.64797700
H	-6.58340900	-1.59450800	2.13691000
C	-7.80254900	0.58415900	1.05360800
H	-8.11654900	1.31807500	0.30695100
H	-8.53070400	-0.23321500	1.05532600
H	-7.85580900	1.06611100	2.03718000
C	-0.80833500	6.34068400	-0.52121700
H	0.08731000	6.95283800	-0.69191100
H	-1.69620300	6.94430600	-0.71662600
H	-0.81962400	6.01287000	0.52722900
C	2.09529800	2.30020500	-1.46550100
C	3.06014500	1.13367500	-1.39846600
H	3.01778400	0.54103700	-2.31616200
H	4.09118400	1.47592700	-1.27362900

4
0 1

C	0.15010400	1.77968900	-0.48160900
C	-0.16574400	1.11408100	0.95663100
C	-0.54091400	3.13380700	-0.69366900
O	-0.97538700	3.87111800	0.16361600
C	1.65463600	2.11335600	-0.64943500
O	2.44467600	1.50645600	-1.33700900
O	-0.55073400	3.44385900	-2.00700100
O	1.98181900	3.20330600	0.06217900
C	-1.11553100	4.72690100	-2.33240300
H	-0.55794000	5.52501600	-1.83572600
H	-1.03746700	4.81444500	-3.41538500
H	-2.16052000	4.77496000	-2.01623400
C	3.38940600	3.49519500	0.13690700
H	3.46738700	4.39279600	0.74869800
H	3.91052200	2.66103600	0.61294300
H	3.80155700	3.66311800	-0.86084700
C	1.85637800	0.20659700	1.73725000
C	0.95492200	1.27209900	1.95102800
C	1.22811000	2.26294000	2.87829800
C	2.41232000	2.18526900	3.62410700
C	3.30230900	1.12887500	3.41240200
C	3.04330000	0.12630800	2.46812400
C	-0.05986100	-0.39582900	0.65022500

H	0.53937800	3.09289400	3.00305500
H	2.64038800	2.94734600	4.36280800
H	4.21793100	1.07513800	3.99485400
H	3.74255500	-0.68602400	2.31088800
H	-0.59723200	-0.98920600	1.40675100
N	1.37630700	-0.66345800	0.73439600
C	3.77756700	-1.44748200	-0.73485800
C	3.99104200	-3.31693300	0.76270100
C	5.07763800	-1.66257700	-1.19816600
H	3.20224500	-0.61550000	-1.12524800
C	5.28325200	-3.54752600	0.30686000
H	3.57823500	-3.96039000	1.53638100
C	5.83574600	-2.71912000	-0.67985900
H	5.48255900	-0.99996600	-1.95351800
H	5.88871300	-4.35566200	0.70359300
O	7.11495300	-3.02144800	-1.05620500
C	-1.61722100	1.34986700	1.40646200
H	-1.78702000	0.73901800	2.29820500
H	-1.80247100	2.39088500	1.66643700
C	-2.59934700	0.93946700	0.29449300
H	-3.60581500	0.83688700	0.70935900
H	-2.65175700	1.71907900	-0.47506200
N	-2.22427600	-0.36509900	-0.31350200
C	-0.78339900	-0.49927500	-0.68998600
C	-0.26105500	0.68665100	-1.51584800
H	-0.66736200	-1.45622900	-1.19865300
H	-1.00879700	1.04296100	-2.22528200
H	0.62354800	0.38607800	-2.07789900
S	-3.31037500	-0.86442000	-1.52414400
O	-2.73117600	-2.07130500	-2.12621600
O	-3.71030900	0.26959800	-2.37489100
C	-4.72449800	-1.32504900	-0.52638000
C	-4.62086300	-2.42510100	0.32891400
C	-5.90953800	-0.60212600	-0.63497100
C	-5.72490500	-2.79257200	1.08876300
H	-3.68972800	-2.97782400	0.38851400
C	-7.00815300	-0.98690000	0.13494900
H	-5.96175100	0.24015200	-1.31592500
C	-6.93310100	-2.07976300	1.00611300
H	-5.65490300	-3.64828400	1.75497500
H	-7.93709600	-0.42943500	0.05387800
C	-8.11284500	-2.48201400	1.85680900
H	-8.26100900	-3.56653800	1.84244100
H	-7.95643800	-2.19112900	2.90251000

H	-9.03659800	-2.00765000	1.51543800
C	7.72615600	-2.21677000	-2.05215300
H	7.81164500	-1.16927800	-1.73329400
H	8.72582500	-2.62807900	-2.20150600
H	7.17535800	-2.25514200	-3.00140700
C	3.21751700	-2.26851400	0.24564500
C	1.79396700	-2.06089000	0.72462900
H	1.10544500	-2.58896800	0.05355300
H	1.66728400	-2.51452100	1.72311100

A-int3-I

0 1

C	-0.33209000	0.44422600	-0.25208900
C	0.42601700	0.77539200	0.91768500
O	1.61840200	1.14831500	0.96696400
C	0.33440300	0.56911700	-1.49831700
O	1.58524800	0.73021000	-1.65181900
O	-0.25930000	0.64220600	2.07650300
O	-0.42754600	0.47366000	-2.59541800
C	0.43851500	1.07926800	3.26052200
H	0.73305800	2.12625200	3.16296400
H	-0.27335600	0.94929900	4.07600000
H	1.33366400	0.47383400	3.42380700
C	0.23825000	0.69866100	-3.85697600
H	-0.56526400	0.76137400	-4.59003900
H	0.83029200	1.61540400	-3.82776600
H	0.89654000	-0.14247100	-4.08854700
C	-0.10659400	-2.64510400	-2.17060500
C	-1.41904700	-2.41739600	-1.73093700
C	-2.44674600	-2.36630400	-2.66289000
C	-2.12629600	-2.55925800	-4.01454900
C	-0.81359600	-2.80948400	-4.42632200
C	0.23412500	-2.85245600	-3.49938800
C	0.08418300	-2.25881800	0.03038000
C	-1.38567500	-2.28975400	-0.21912500
H	-3.46123900	-2.12449400	-2.37149300
H	-2.91615400	-2.50225600	-4.75666500
H	-0.59708000	-2.95470400	-5.47974500
H	1.25885400	-3.01878800	-3.81206700
H	0.57355500	-2.15332100	0.98854800
N	0.76508400	-2.56889700	-1.04624900
C	2.99450300	-3.82181700	0.96269400
C	3.38813200	-1.45386200	0.72814300
C	3.53703000	-3.79307300	2.24806000

H	2.63511000	-4.76700600	0.56109600
C	3.92956900	-1.40644800	2.00349000
H	3.33531600	-0.54014400	0.15316300
C	4.00455000	-2.57721200	2.77474600
H	3.59801800	-4.71064300	2.82035100
H	4.30458400	-0.46977300	2.40080600
O	4.54336000	-2.43374500	4.01360600
C	-2.09523100	-3.48725300	0.49357200
H	-1.91966000	-4.44004400	-0.00978900
H	-1.72715100	-3.56292800	1.52171300
C	-3.57578900	-3.07492100	0.47646900
H	-4.06041000	-3.40754800	-0.44765100
H	-4.13511100	-3.49788600	1.31391900
N	-3.53011500	-1.59536400	0.56268200
C	-2.13116200	-1.11074900	0.46201500
C	-1.82945600	0.24832400	-0.17828500
H	-1.74684400	-1.06483100	1.48716500
H	-2.27701200	1.03232400	0.43674400
H	-2.26844600	0.30730900	-1.17318900
S	-4.87058900	-0.77831100	-0.04754900
O	-6.00002200	-1.65075000	0.29357900
O	-4.68261600	-0.36826900	-1.45106700
C	-4.87226600	0.70008700	0.95080800
C	-4.83474300	0.59085500	2.34220800
C	-4.91711500	1.94483000	0.32140100
C	-4.82081800	1.75300800	3.10806400
H	-4.80231100	-0.38706900	2.81036100
C	-4.90540100	3.09584200	1.10537800
H	-4.93748800	1.99917200	-0.76112400
C	-4.84851000	3.01999200	2.50453200
H	-4.78430600	1.67811600	4.19138300
H	-4.92888300	4.06878600	0.62277400
C	-4.78283800	4.27624000	3.33707200
H	-5.39937000	5.07168300	2.90807200
H	-5.11682300	4.09841800	4.36302300
H	-3.75377000	4.65268900	3.38328400
C	4.68554800	-3.58551400	4.83334300
H	5.33545800	-4.33675800	4.36615100
H	5.14570700	-3.23938600	5.75968000
H	3.71364500	-4.04283100	5.06239000
In	2.63392800	2.33350900	-0.67620700
Cl	4.60292500	1.93498600	0.57559200
Cl	1.04550600	4.02113500	-0.16229600
Cl	3.46384800	2.90805400	-2.84175600

C	2.91081900	-2.65798600	0.19125000
C	2.24799500	-2.66644800	-1.15507100
H	2.56230400	-1.81612900	-1.76706200
H	2.44619300	-3.59065700	-1.70696100

A-int3-R

0 1

C	0.62004800	0.13365200	-0.06939900
C	-0.39194500	1.11210500	0.06191400
O	-1.48320400	1.17372400	-0.55026800
C	0.55566000	-0.83049300	-1.13750600
O	-0.28479900	-0.97791400	-2.01438600
O	-0.09508900	2.10627300	0.96180600
O	1.69479700	-1.64620900	-1.13479000
C	-1.11934200	3.09673800	1.13955800
H	-2.04373100	2.63681500	1.49236600
H	-0.72167300	3.78854800	1.88597200
H	-1.30974300	3.62394300	0.20102500
C	1.76499600	-2.55932700	-2.22903700
H	2.68961000	-3.12395900	-2.08824800
H	0.90875700	-3.23739000	-2.23928200
H	1.78864300	-2.02910600	-3.18721600
C	5.84148100	-0.86791800	0.98409400
C	4.74949300	-0.30128800	1.65717400
C	4.22695600	-0.95504400	2.76983500
C	4.79995100	-2.16751700	3.16363400
C	5.88064800	-2.72165000	2.46158700
C	6.42812100	-2.07483000	1.35257600
C	5.41551200	1.07276100	-0.07029500
C	4.35968000	0.96562100	0.95160000
H	3.38278800	-0.54057300	3.31029300
H	4.39995200	-2.69130100	4.02587600
H	6.30599200	-3.66457700	2.78983500
H	7.28139200	-2.47983900	0.82223000
H	5.49412000	1.82624100	-0.84289900
N	6.19388100	0.00654300	-0.07834300
C	9.50399500	-1.33220500	-0.59715800
C	9.05448200	0.83056000	0.35824500
C	10.77789700	-1.33585100	-0.02232600
H	9.19510100	-2.18533300	-1.19675400
C	10.31148200	0.84075000	0.94138000
H	8.38806100	1.67498700	0.51519000
C	11.18562500	-0.24486200	0.75545200
H	11.43061200	-2.18506400	-0.18238900

H	10.64824300	1.67508200	1.54712800
O	12.39065900	-0.13693500	1.37159500
C	4.07172400	2.22988100	1.80464100
H	3.69651800	1.91264100	2.78061200
H	4.98643600	2.80197000	1.97935800
C	2.99857700	3.05303300	1.03376700
H	2.07113900	3.12042500	1.60814700
H	3.33570800	4.05670600	0.77780300
N	2.74214800	2.28653600	-0.18744900
C	2.98387700	0.88668000	0.04476000
C	1.83063500	0.17105600	0.80355800
H	3.15926200	0.34773900	-0.88438200
H	1.62417200	0.69170500	1.74106700
H	2.17623300	-0.83782000	1.03329700
S	2.96287400	2.97600700	-1.72520800
O	3.80959100	2.03410800	-2.48574800
O	3.41780000	4.35471100	-1.50029500
C	1.33228600	2.98817700	-2.41199700
C	0.82800700	1.81717900	-2.98255000
C	0.52047200	4.10578000	-2.20169000
C	-0.52071200	1.75507300	-3.31080000
H	1.46959500	0.95772600	-3.13214900
C	-0.82303100	4.02837100	-2.55925200
H	0.93735100	5.00621200	-1.76400800
C	-1.37058900	2.84525500	-3.07914200
H	-0.92965400	0.81799900	-3.66843200
H	-1.46728700	4.88866500	-2.39747500
C	-2.85292900	2.70776100	-3.29538600
H	-3.30487400	2.27259500	-2.39592900
H	-3.32837700	3.67539700	-3.48559600
H	-3.07663300	2.03705000	-4.13006800
C	13.32364200	-1.20149200	1.22987600
H	13.60609100	-1.35255800	0.18010300
H	14.20469600	-0.90568000	1.80042200
H	12.92748600	-2.14084800	1.63673100
Rh	-5.06378000	-1.50020700	0.69009500
O	-4.84112900	-0.64828800	2.58186000
O	-6.26557800	0.08907400	0.08009100
O	-3.06143200	0.58691800	1.90988200
O	-4.46916400	1.34097200	-0.51743500
C	-3.93394700	0.21980100	2.76337900
C	-3.87467900	0.90035500	4.12373200
C	-5.24389400	1.33105700	4.68120600
H	-5.90504400	0.45711000	4.68064600

H	-5.10408200	1.62430800	5.72910100
C	-7.70877700	2.60231500	0.40002900
H	-8.41718400	3.32872400	-0.01701000
H	-8.26588300	1.67370200	0.56763500
C	-6.62851700	2.33029900	-0.66283900
C	-5.71774400	1.15388500	-0.34219800
Rh	-3.15433100	-0.17885300	-0.01532800
O	-3.41262100	-0.99381000	-1.90262300
O	-1.95713200	-1.76618200	0.59416100
O	-5.14146300	-2.29418500	-1.20978800
O	-3.75439400	-3.00260300	1.22357700
C	-4.29148600	-1.89447000	-2.07229200
C	-4.32547700	-2.56551500	-3.43698700
C	-2.93928700	-2.98949700	-3.96155000
H	-2.26745200	-2.12659900	-3.89682600
H	-3.04433200	-3.24472900	-5.02360700
C	-0.53592500	-4.30786400	0.30382100
H	0.16401900	-5.03838700	0.72999000
H	0.03941800	-3.39815400	0.10054600
C	-1.59184000	-3.98893100	1.37826200
C	-2.50785500	-2.82260300	1.03696300
H	-5.00993500	-3.41692000	-3.37902700
H	-4.76370300	-1.83726200	-4.13139300
H	-7.11563000	2.09713700	-1.61822700
H	-5.99865400	3.21020400	-0.82356600
H	-3.41696900	0.17975200	4.81361900
H	-3.19259300	1.75219700	4.04390800
H	-2.22231900	-4.85886300	1.58399400
H	-1.08276400	-3.72219800	2.31342800
C	-2.29607500	-4.17636400	-3.21917200
H	-2.93546100	-5.06684500	-3.30928100
H	-1.35311000	-4.42202300	-3.72862900
C	-2.00818200	-3.86328700	-1.74639000
H	-1.55558100	-2.87055300	-1.69248300
H	-2.95869600	-3.80373000	-1.20798700
C	-1.10054200	-4.86349000	-1.01767700
H	-0.25210700	-5.13065900	-1.66434100
H	-1.63931400	-5.80283900	-0.82623500
C	-7.18491800	3.13097100	1.74881600
H	-6.65839700	4.08412800	1.59422200
H	-8.05064800	3.35896900	2.38616800
C	-6.26859900	2.13650500	2.47128300
H	-6.72896600	1.14388300	2.44341500
H	-5.33622200	2.04959800	1.90618900

C	-5.92785900	2.48560300	3.92459300
H	-6.84955800	2.74737000	4.46264700
H	-5.28902400	3.38032000	3.95785000
C	8.63115200	-0.25921000	-0.41885500
C	7.26024600	-0.26189700	-1.05721400
H	7.16484000	0.50309500	-1.83334200
H	7.04646500	-1.23006100	-1.51988500

A-int4-R

0 1

C	1.78248400	-0.10120700	-0.58482600
C	0.79608800	-0.45076000	-1.68830100
O	-0.12963100	-1.24030700	-1.57317300
C	1.98089500	-1.34079500	0.28486100
O	2.10814900	-2.45754700	-0.16962800
O	0.97183900	0.26821000	-2.79864200
O	2.12710000	-1.04736400	1.58578800
C	-0.09254100	0.17112200	-3.78097800
H	-1.04021500	0.45012400	-3.31605000
H	0.18697900	0.86835600	-4.56932000
H	-0.15690000	-0.85034500	-4.16115300
C	2.57484000	-2.13154900	2.41865200
H	2.62295100	-1.71953700	3.42493800
H	1.87633200	-2.96877200	2.36988800
H	3.56589700	-2.45979300	2.09718100
C	4.72371400	0.24858000	0.61048800
C	4.08338500	1.50033200	0.71225600
C	4.20871600	2.25371600	1.87008700
C	5.00062100	1.76471400	2.92226900
C	5.66615700	0.54470800	2.79063300
C	5.53917100	-0.23534800	1.63381400
C	3.25492500	0.34236400	-1.16459700
C	3.38928100	1.77903700	-0.62252000
H	3.67537700	3.19318300	1.97032100
H	5.09830400	2.34125200	3.83684200
H	6.29024100	0.18314200	3.60344800
H	6.05631400	-1.18323500	1.53481700
H	3.25237700	0.26587600	-2.25160900
N	4.37905200	-0.36622500	-0.58983000
C	7.15049500	-2.76602600	-0.90850600
C	7.03967500	-0.55012000	-1.82473200
C	8.53218700	-2.80248500	-1.12390100
H	6.66563600	-3.62842000	-0.45619500
C	8.40985900	-0.56496900	-2.04258400

H	6.46215100	0.33382400	-2.07997800
C	9.16783700	-1.69444200	-1.69452700
H	9.09212100	-3.68435100	-0.83676400
H	8.92421800	0.28632700	-2.47646700
O	10.50687000	-1.60624300	-1.94544600
C	4.06696300	2.93633400	-1.37279600
H	5.15456300	2.95180900	-1.27278600
H	3.81028400	2.89790900	-2.43698900
C	3.40981500	4.16721900	-0.69565900
H	4.01569300	4.54419300	0.13045700
H	3.25211000	4.99102000	-1.39409800
N	2.09680100	3.66465900	-0.11246000
C	1.95406200	2.27643700	-0.59226100
C	1.20062600	1.17945300	0.14122000
H	1.59028800	2.29783500	-1.62859400
H	0.11653200	1.22632600	0.06107500
H	1.46240600	1.15378700	1.19766300
S	0.78523900	4.68856800	-0.49352600
O	0.22128100	4.32397300	-1.80560000
O	1.26292600	6.05376300	-0.24595300
C	-0.43487000	4.27753400	0.74560800
C	-1.47749000	3.41236700	0.41306200
C	-0.37602200	4.89447000	1.99588200
C	-2.48000300	3.16980600	1.34838500
H	-1.49515000	2.93938800	-0.56087100
C	-1.38423900	4.63502700	2.92303500
H	0.43455300	5.57839300	2.22275700
C	-2.45165000	3.77910200	2.61148600
H	-3.28951900	2.48749100	1.11176800
H	-1.34671000	5.10983600	3.89977900
C	-3.55738300	3.50335100	3.60042500
H	-3.23291900	3.68297400	4.62938800
H	-4.41985800	4.15286300	3.40754900
H	-3.90134600	2.46794400	3.51738600
C	11.32726600	-2.71171500	-1.60245800
H	11.03904400	-3.61831600	-2.15158100
H	12.34430600	-2.43217800	-1.88233600
H	11.29540300	-2.92249700	-0.52490800
Rh	-4.26541400	-0.99118400	0.66064300
O	-4.51245400	0.83018800	-0.33259700
O	-5.05714900	-1.99935300	-0.96743300
O	-2.48430200	0.66155600	-1.32877000
O	-3.03966900	-2.07837900	-1.99995800
C	-3.62230800	1.21710200	-1.15385200

C	-3.90717600	2.46507800	-1.97396200
C	-5.33774800	2.56980100	-2.53236400
H	-6.04201100	2.42898500	-1.70469900
H	-5.47637900	3.59378700	-2.89820600
C	-6.25123800	-2.32517800	-3.61288000
H	-6.70148100	-2.99323500	-4.35663100
H	-6.93450300	-2.29524700	-2.75671800
C	-4.92491300	-2.95873800	-3.15343700
C	-4.29430600	-2.29335100	-1.94245600
Rh	-2.11502400	-1.16595900	-0.40212000
O	-1.90467900	-3.00642100	0.50825200
O	-1.30651900	-0.24491200	1.28537100
O	-3.89937600	-2.79895900	1.56983400
O	-3.34089600	-0.05030400	2.26028100
C	-2.80850600	-3.40562800	1.31016200
C	-2.56582400	-4.72318700	2.02552700
C	-1.13443300	-4.89441100	2.56801800
H	-0.43097200	-4.70751300	1.74862400
H	-1.00895500	-5.94226100	2.86520300
C	-0.13133700	-0.10738900	3.96598900
H	0.32525600	0.50684700	4.75146000
H	0.56354100	-0.11298400	3.12192100
C	-1.42540900	0.59607100	3.52395400
C	-2.07004700	0.05317000	2.25971600
H	-3.31010600	-4.81370900	2.82203700
H	-2.77192200	-5.51427600	1.29346300
H	-5.10264500	-4.00467000	-2.87277500
H	-4.18398800	-2.95914600	-3.95804100
H	-3.72427300	3.31657200	-1.30754300
H	-3.16010500	2.52189900	-2.77123200
H	-2.18143700	0.57730900	4.31419700
H	-1.19742400	1.64657100	3.31583600
C	-0.78412700	-3.98656200	3.76132100
H	-1.44143400	-4.21788100	4.61138800
H	0.23648500	-4.22906900	4.08957700
C	-0.86869600	-2.49440900	3.42490800
H	-0.35568100	-2.31255100	2.47588700
H	-1.91724100	-2.23434000	3.24943600
C	-0.30992500	-1.54352100	4.48810000
H	0.66672100	-1.90976700	4.83279300
H	-0.96238500	-1.54332400	5.37268400
C	-6.12426800	-0.91346500	-4.21454800
H	-5.48024000	-0.94580800	-5.10507500
H	-7.11663500	-0.59883500	-4.56485400

C	-5.58695700	0.11775700	-3.21628500
H	-6.11966600	0.00444300	-2.26648400
H	-4.53954500	-0.11805000	-3.00252900
C	-5.67182400	1.58013600	-3.66279200
H	-6.68767300	1.79589600	-4.02034300
H	-5.00166500	1.75340900	-4.51701800
C	6.38550500	-1.65326400	-1.25634000
C	4.88274800	-1.65099500	-1.03749500
H	4.35749500	-1.88731000	-1.96940400
H	4.60991500	-2.45167600	-0.33703400

A-TS3-I
0 1

C	-0.11398500	0.29453400	-0.32221700
C	0.63245700	0.64481000	0.83558100
O	1.87737400	0.75114300	0.92965500
C	0.57741800	0.20485000	-1.54310900
O	1.82744600	0.34692900	-1.71952200
O	-0.12250500	0.87320500	1.93968000
O	-0.17334100	-0.06840300	-2.63174900
C	0.59163400	1.40700200	3.07222000
H	1.10683400	2.32787100	2.79003300
H	-0.17360500	1.60176500	3.82456200
H	1.32442200	0.68629500	3.44247700
C	0.50425500	0.00688500	-3.90165100
H	-0.28165300	-0.11606300	-4.64651600
H	1.01525800	0.96529800	-4.01401400
H	1.24677600	-0.79098700	-3.99288800
C	-1.67183200	-2.77692900	-2.13285900
C	-2.87499700	-2.40777900	-1.49861500
C	-3.95634500	-1.98749300	-2.27874000
C	-3.80153200	-1.96146500	-3.66438900
C	-2.59471200	-2.34592800	-4.27461400
C	-1.50265100	-2.75922600	-3.51420800
C	-1.33616600	-3.04755400	0.06148100
C	-2.66417600	-2.53106500	-0.05233600
H	-4.87964000	-1.65827600	-1.81713300
H	-4.62917400	-1.63430500	-4.28573200
H	-2.50927800	-2.31612400	-5.35630500
H	-0.56389900	-3.03959600	-3.97841900
H	-0.78057600	-3.29056700	0.95651300
N	-0.74935500	-3.13650800	-1.13129500
C	1.55203300	-4.66299400	0.62935000
C	2.24600700	-2.38143800	0.26796100

C	2.28076100	-4.70375600	1.82021800
H	1.00647100	-5.54963600	0.31260300
C	2.97869300	-2.40516000	1.44484000
H	2.25796700	-1.47981800	-0.33087100
C	2.99576800	-3.56774500	2.23260500
H	2.29506000	-5.61626400	2.40372000
H	3.55195900	-1.53536600	1.74870000
O	3.73303100	-3.49648500	3.37202400
C	-3.78948400	-2.77937200	0.93442300
H	-4.73825800	-2.70687500	0.39559500
H	-3.73895600	-3.78524300	1.36214000
C	-3.77587500	-1.71682100	2.06830300
H	-4.74277200	-1.59520900	2.55433700
H	-3.03154100	-1.96936600	2.83184500
N	-3.35540900	-0.45992100	1.44287800
C	-2.16608800	-0.64253800	0.76450800
C	-1.61808300	0.30930400	-0.26316100
H	-1.42038800	-1.04443300	1.44026100
H	-1.94491000	1.31251400	0.01994700
H	-2.06380400	0.11035100	-1.23934600
S	-4.61979300	0.63187100	0.89773000
O	-5.69564600	0.40885800	1.86405300
O	-4.85156700	0.44621500	-0.53859700
C	-3.79830900	2.18897500	1.15443900
C	-3.15816600	2.43566400	2.37168200
C	-3.68730100	3.07388200	0.07836000
C	-2.35112100	3.56206700	2.48514100
H	-3.25105400	1.73514100	3.19407600
C	-2.87895700	4.19831600	0.21838300
H	-4.18042500	2.84928800	-0.86071000
C	-2.16964500	4.44154000	1.40510700
H	-1.82301300	3.74724800	3.41575400
H	-2.76195900	4.87707300	-0.62103800
C	-1.18331700	5.57502500	1.49215600
H	-1.48500300	6.42094100	0.86831100
H	-1.06021500	5.92617100	2.52049000
H	-0.20396500	5.23162000	1.13466500
C	3.84489600	-4.65541500	4.18481500
H	4.30015400	-5.49119800	3.63752900
H	4.49214500	-4.37688100	5.01744700
H	2.86913800	-4.97256700	4.57740000
In	3.10810800	1.69488200	-0.65931700
Cl	4.95314300	0.74616400	0.47591900
Cl	1.94508300	3.69314600	0.04638800

Cl	3.97207900	2.36775900	-2.77830100
C	1.51689000	-3.50546400	-0.15439400
C	0.68144400	-3.43778100	-1.40299400
H	1.03702100	-2.64143500	-2.06279800
H	0.70299200	-4.37895800	-1.96305800

A-TS3-n

0 1

C	0.05403200	-0.31606500	1.57606400
C	0.46518400	-1.68346700	1.62114300
O	1.32714800	-2.24209100	2.29278900
C	0.61989600	0.63519700	2.47722400
O	1.65535800	0.55255800	3.14322800
O	-0.30296800	-2.45919000	0.71901300
O	-0.14778600	1.79914800	2.55902900
C	0.03890600	-3.84046800	0.69839300
H	-0.08222500	-4.29860100	1.68504200
H	-0.64117500	-4.30821200	-0.01863300
H	1.07770100	-3.99312800	0.38528100
C	0.26644300	2.71658000	3.56376700
H	-0.38683000	3.58551700	3.46317600
H	0.16684800	2.27948300	4.56346100
H	1.30948700	3.02040800	3.43462000
C	1.31601800	2.94808300	-0.05571600
C	0.19772500	2.77784500	-0.89986800
C	-0.81042800	3.74649300	-0.89343000
C	-0.67010500	4.84648600	-0.04910900
C	0.45779100	4.99781600	0.77550300
C	1.47456400	4.04608700	0.78460300
C	1.66671600	1.06244800	-1.19844100
C	0.39861100	1.53238100	-1.64258000
H	-1.69368800	3.63164300	-1.50977600
H	-1.44807300	5.60332100	-0.02816600
H	0.53736000	5.86802300	1.41949000
H	2.34288400	4.15007100	1.42491200
H	2.20157000	0.17407300	-1.50166600
N	2.18283200	1.86613500	-0.26237100
C	4.94619100	0.17946100	-0.84628200
C	3.67575800	-0.83063100	0.94207500
C	5.46272100	-1.06931600	-1.20231900
H	5.24764900	1.05756600	-1.41369600
C	4.16912600	-2.07821200	0.58832100
H	2.99074500	-0.74849700	1.78095300
C	5.06518200	-2.20616700	-0.48348500

H	6.16274200	-1.14151200	-2.02583600
H	3.85776800	-2.96052700	1.13603200
O	5.49244100	-3.47537500	-0.75019100
C	-0.18971100	1.28392600	-3.01745600
H	-0.89938100	2.08746900	-3.23546100
H	0.57835300	1.31902700	-3.79686300
C	-0.94308800	-0.06864600	-3.09274900
H	-1.65047900	-0.11488400	-3.91901300
H	-0.24472400	-0.90729900	-3.18034000
N	-1.64080000	-0.22608500	-1.80900900
C	-0.74714700	-0.14843900	-0.76559000
C	-1.09145600	0.06880000	0.67683000
H	0.08121400	-0.82626300	-0.92874900
H	-1.97119000	-0.56585500	0.86166200
H	-1.41204500	1.09954200	0.83636100
S	-3.24496000	0.47128700	-1.70763800
O	-3.75650500	0.36739200	-3.07613800
O	-3.19585700	1.76657900	-1.02452500
C	-4.05357400	-0.71171600	-0.65046100
C	-4.06169800	-2.06058200	-1.01092000
C	-4.68173400	-0.25892500	0.50923600
C	-4.70035600	-2.97004700	-0.17601600
H	-3.56536900	-2.39005400	-1.91683300
C	-5.32299300	-1.18676600	1.32620300
H	-4.64675500	0.79310400	0.76800600
C	-5.33503800	-2.55058200	1.00396500
H	-4.70408000	-4.02377200	-0.43928400
H	-5.81109300	-0.84665000	2.23473800
C	-5.98275000	-3.55494400	1.92352800
H	-6.40093600	-4.39773200	1.36571200
H	-5.24498800	-3.96073400	2.62617600
H	-6.78347900	-3.10127100	2.51374600
C	6.42142900	-3.66674600	-1.80450000
H	7.35737800	-3.12045200	-1.62677400
H	6.63188400	-4.73705400	-1.83136600
H	6.00801500	-3.35764700	-2.77435500
C	4.05126300	0.31206600	0.21796300
C	3.40987000	1.63209600	0.54279100
H	3.10002300	1.65956200	1.59108000
H	4.08130700	2.47397000	0.34046000

A-TS3-R

0 1

C	0.73133000	0.50415200	0.18053500

C	-0.41820500	1.31995900	0.32154000
O	-1.48546200	1.26523600	-0.32905400
C	0.85466900	-0.39204700	-0.93901400
O	0.08510300	-0.60179200	-1.86636800
O	-0.29067700	2.28844900	1.28394700
O	2.10110800	-1.03635300	-0.92313600
C	-1.44529800	3.12337200	1.46407000
H	-2.31593300	2.52478800	1.73928300
H	-1.17764200	3.81258300	2.26809700
H	-1.66201500	3.67866600	0.54699700
C	2.34543900	-1.86625900	-2.05718200
H	3.33808200	-2.29787400	-1.90344000
H	1.60194800	-2.66202800	-2.13892400
H	2.32558100	-1.28762800	-2.98706400
C	5.51029700	-0.17604500	1.41217100
C	4.68931500	0.71686200	2.12371500
C	4.23795000	0.36675700	3.39548900
C	4.60489900	-0.87588900	3.91365300
C	5.40410000	-1.76272200	3.17367400
C	5.87571400	-1.42654800	1.90447900
C	5.30947800	1.65819300	0.13055100
C	4.43062200	1.88685300	1.25232800
H	3.60179000	1.03937500	3.96218400
H	4.26145500	-1.16655900	4.90135900
H	5.66815800	-2.72567600	3.59897900
H	6.51275400	-2.09696000	1.34054400
H	5.41215600	2.28606400	-0.74575200
N	5.85140200	0.44169400	0.18583500
C	8.45276500	-1.90422700	-0.77330000
C	8.85689500	0.30935600	0.07928600
C	9.75744900	-2.28346300	-0.44499000
H	7.79322400	-2.63477200	-1.23577700
C	10.15178000	-0.05157100	0.41763600
H	8.51059500	1.31734700	0.29228100
C	10.61416300	-1.35316600	0.15599900
H	10.08584300	-3.29373800	-0.65546800
H	10.83177800	0.65194200	0.88586900
O	11.89738600	-1.60389400	0.52596700
C	4.19054200	3.29770400	1.80598600
H	4.37074700	3.32110900	2.88424700
H	4.88562700	4.00147300	1.33996000
C	2.73858600	3.70662100	1.44439300
H	2.01763300	3.36604600	2.19254200
H	2.61946300	4.77981300	1.30589700

N	2.45426900	2.99394200	0.20310700
C	2.85399300	1.64704700	0.31121500
C	1.90879600	0.70350400	1.08040500
H	3.09667400	1.19834500	-0.64756500
H	1.62283400	1.13341900	2.04047000
H	2.43886100	-0.23030900	1.26145000
S	2.65443300	3.80551900	-1.30770800
O	3.65052800	3.03735000	-2.07977900
O	2.89357200	5.21543500	-0.97932800
C	1.05722100	3.60319300	-2.03898200
C	0.74145000	2.38564100	-2.64818400
C	0.09173800	4.59197000	-1.83559400
C	-0.57159200	2.14819400	-3.03396300
H	1.49915100	1.62533400	-2.79007300
C	-1.21218900	4.34234000	-2.25608600
H	0.36377300	5.52735700	-1.35911600
C	-1.56918300	3.11213500	-2.82996000
H	-0.83401700	1.17231900	-3.42458500
H	-1.97351100	5.10376700	-2.10889900
C	-3.00367100	2.79119600	-3.14842600
H	-3.43827800	2.22384900	-2.31712400
H	-3.59987200	3.69713000	-3.29669500
H	-3.08476500	2.16956800	-4.04523700
C	12.43160200	-2.89975500	0.28502200
H	12.43396400	-3.14242000	-0.78546000
H	13.45907100	-2.87319300	0.64983300
H	11.87470600	-3.67346700	0.82933200
Rh	-4.72680800	-1.97936300	0.35454300
O	-4.91452700	-1.17764500	2.27039200
O	-6.08953600	-0.58404400	-0.37930800
O	-3.27932200	0.34673600	1.88645100
O	-4.46205300	0.96721700	-0.68731700
C	-4.19468000	-0.18449200	2.59622600
C	-4.43850700	0.44264900	3.96189300
C	-5.92286100	0.61189300	4.33427400
H	-6.41754500	-0.35975100	4.22452000
H	-5.97511700	0.87809100	5.39725400
C	-7.96459000	1.64670100	-0.22379300
H	-8.71703300	2.26704500	-0.72605000
H	-8.37768700	0.63376700	-0.15894000
C	-6.71370900	1.60050500	-1.12036200
C	-5.67435600	0.57320400	-0.69603500
Rh	-2.98613200	-0.34027000	-0.05233800
O	-2.86014100	-1.09764200	-1.97737300

O	-1.62811300	-1.73652300	0.66776300
O	-4.41396500	-2.69784000	-1.55317100
O	-3.26330100	-3.26816300	1.03570400
C	-3.54145600	-2.12401100	-2.28413000
C	-3.28280600	-2.73038700	-3.65482500
C	-1.78847100	-2.91922800	-3.98413600
H	-1.27008500	-1.97306500	-3.79446300
H	-1.70406100	-3.13354500	-5.05701900
C	0.21193300	-4.01039100	0.52719100
H	0.96528100	-4.62835900	1.03272000
H	0.65109500	-3.01447300	0.39928200
C	-1.00541300	-3.89572400	1.46361600
C	-2.05023200	-2.88311800	1.01936500
H	-3.82939400	-3.67599500	-3.71574000
H	-3.72657900	-2.04446000	-4.38760500
H	-7.01382400	1.33622300	-2.14243600
H	-6.22241600	2.57690700	-1.16695400
H	-3.95689800	-0.21548800	4.69653700
H	-3.91037400	1.40053900	3.99000400
H	-1.50526800	-4.86154300	1.58158100
H	-0.66498100	-3.57651900	2.45700900
C	-1.08299500	-4.03516600	-3.19012900
H	-1.55560300	-5.00591200	-3.39936600
H	-0.05060300	-4.11112300	-3.56169200
C	-1.05978300	-3.75635800	-1.68238800
H	-0.80826600	-2.70322700	-1.54061000
H	-2.06988500	-3.89054700	-1.28391500
C	-0.08942500	-4.61402600	-0.85780300
H	0.86337600	-4.71513600	-1.39735100
H	-0.47967700	-5.63549100	-0.74338800
C	-7.73143400	2.19254100	1.19780700
H	-7.35815600	3.22559400	1.14163700
H	-8.70301200	2.24489500	1.70846200
C	-6.76830800	1.33061100	2.02208900
H	-7.04744800	0.27831300	1.90934300
H	-5.76654400	1.42149800	1.59263700
C	-6.68990600	1.66730900	3.51520600
H	-7.70623500	1.74606600	3.92559100
H	-6.22570700	2.65459900	3.65505000
C	7.98717500	-0.61399200	-0.52159400
C	6.58130300	-0.21395200	-0.90804000
H	6.57734300	0.49065800	-1.74552100
H	5.99950400	-1.08928600	-1.21570600

A-TS4-I

0 1

C	0.21727800	-0.14914300	-0.14377000
C	-0.57655400	-0.39959700	1.08107100
O	-1.70497900	-0.89503400	1.15159800
C	-0.42137000	-0.69614500	-1.35282000
O	-1.66479000	-0.73566400	-1.50316200
O	0.03034300	0.04457200	2.18492000
O	0.38393800	-1.08388600	-2.31489100
C	-0.71659400	-0.10748900	3.41798000
H	-0.94433100	-1.16054600	3.59188400
H	-0.06228000	0.28613600	4.19456800
H	-1.64830300	0.45981600	3.35943400
C	-0.23804400	-1.56216700	-3.53806700
H	0.58591800	-1.95401500	-4.13071700
H	-0.97917400	-2.33005800	-3.31214100
H	-0.71473600	-0.71934100	-4.04254700
C	0.12028500	2.11350100	-2.53676000
C	1.45348100	1.91459700	-2.12017500
C	2.44242600	1.70317100	-3.06932500
C	2.08576600	1.69736500	-4.42783600
C	0.76579100	1.91744800	-4.82403700
C	-0.24757500	2.12515700	-3.87784800
C	0.00801400	1.83954700	-0.30362700
C	1.47738400	2.06673400	-0.59866800
H	3.46255800	1.48835700	-2.77753500
H	2.84945200	1.51174000	-5.17628000
H	0.51111300	1.91147600	-5.87964500
H	-1.27894600	2.26970500	-4.18116700
H	-0.40338900	2.10319600	0.66342900
N	-0.71096500	2.23416900	-1.40746100
C	-2.53890600	3.82965200	0.58709000
C	-3.47277000	1.61501200	0.52626000
C	-2.98842900	3.99457500	1.89838100
H	-1.99161300	4.63897200	0.10913400
C	-3.92741200	1.75718700	1.83037900
H	-3.67226400	0.68669900	0.00700600
C	-3.67970900	2.94774800	2.52937000
H	-2.79912700	4.92919200	2.41230600
H	-4.46737800	0.95074400	2.31428800
O	-4.14321100	2.98964700	3.80946500
C	2.17572700	3.38675000	-0.18503200
H	1.98414600	4.21486500	-0.86992800
H	1.85370100	3.68123500	0.81962300

C	3.66955000	2.97345300	-0.18133300
H	4.10871900	3.10334100	-1.17541700
H	4.26724200	3.55241700	0.52587800
N	3.64735000	1.53384100	0.21246100
C	2.24473100	1.09043000	0.29011300
C	1.74741800	-0.31265900	-0.03025300
H	1.92323700	1.30150700	1.31850700
H	2.01050300	-1.02909200	0.74976700
H	2.15556500	-0.66525600	-0.97575400
S	4.89936400	0.54502900	-0.29463900
O	6.09699500	1.38697300	-0.20257000
O	4.59827000	-0.14194700	-1.56428200
C	4.87239200	-0.69764300	0.99041900
C	4.98208800	-0.30700900	2.32724100
C	4.74226800	-2.04061200	0.63764300
C	4.94524600	-1.28176800	3.31926900
H	5.07800900	0.74379000	2.57905000
C	4.70757900	-3.00317500	1.64563000
H	4.64945600	-2.31391800	-0.40732700
C	4.80715600	-2.64168300	2.99591400
H	5.02064400	-0.98617500	4.36232100
H	4.59221600	-4.04978000	1.37842200
C	4.79138900	-3.69162600	4.07945200
H	5.81337500	-3.99558900	4.33712700
H	4.32286700	-3.31693500	4.99435100
H	4.25269800	-4.58838700	3.76116100
C	-3.96127500	4.18271700	4.55785800
H	-4.45655300	5.03928600	4.08211000
H	-4.41825400	3.99981100	5.53141400
H	-2.89714500	4.41687200	4.69771100
In	-2.81039800	-2.23008400	-0.37393700
Cl	-4.65194000	-1.81952200	1.02598000
Cl	-1.05382400	-3.76036300	0.04795800
Cl	-3.76880900	-2.85075600	-2.45252800
C	-2.76400000	2.63958300	-0.11290400
C	-2.17099500	2.42204000	-1.48148600
H	-2.62381200	1.55188700	-1.96643100
H	-2.33790800	3.29350700	-2.12580800

B-int3-I

0 1

C	-0.11177100	-1.12166600	0.44322800
C	0.15255300	-1.85084000	-0.85557000
O	1.25157300	-1.84834400	-1.40962300

C	0.97342600	-1.51843900	1.43940200
O	2.14559000	-1.74294800	1.14601800
O	-0.89185400	-2.44171600	-1.39946600
O	0.55772400	-1.56654600	2.69129500
C	-0.66609900	-3.07061400	-2.69017000
H	0.11585400	-3.82682300	-2.60226900
H	-1.62355700	-3.51516000	-2.95548600
H	-0.36404600	-2.31557300	-3.41787700
C	1.59756800	-1.70927300	3.69799500
H	1.07091800	-1.68291000	4.64970700
H	2.12473000	-2.65399100	3.55737200
H	2.29506800	-0.87458800	3.60722000
C	1.52159600	1.38136400	1.59212800
C	0.27000200	1.43372900	2.22601000
C	0.17080700	1.79907700	3.56382800
C	1.33701400	2.12588400	4.26544500
C	2.57969300	2.08770800	3.62184600
C	2.69077900	1.70920500	2.27951300
C	0.04265600	0.46297800	0.10742000
C	-0.81510900	1.13466800	1.21152800
H	-0.79784200	1.83945600	4.05573700
H	1.27775200	2.41167000	5.31126200
H	3.47956100	2.34019500	4.17478900
H	3.65595100	1.63707700	1.79311300
H	-0.34899800	0.62808900	-0.89453600
N	1.41544800	0.92678700	0.24473100
C	0.22966500	3.16936000	-2.07489800
C	1.28256400	4.16399600	-0.15659500
C	-0.58780700	4.29312000	-2.22942800
H	0.12918500	2.34702000	-2.77908700
C	0.48062900	5.28865200	-0.29403500
H	2.00714000	4.11916600	0.65117000
C	-0.46913700	5.35690100	-1.32711200
H	-1.30193800	4.32388100	-3.04313800
H	0.56744700	6.13296800	0.38200400
O	-1.21565600	6.49665600	-1.36586100
C	-1.60450400	2.42024500	0.82506400
H	-1.13293700	3.32213000	1.21542300
H	-1.66679700	2.52516600	-0.25979500
C	-3.00089100	2.16017500	1.40437300
H	-3.02319100	2.31873300	2.48876800
H	-3.79988800	2.74846300	0.95560200
N	-3.22664400	0.70498200	1.18487900
C	-1.91923300	0.10397800	1.60536500

C	-1.53330400	-1.27127700	1.01970000
H	-1.98946000	0.03632100	2.69481100
H	-2.19590900	-1.58005900	0.22265900
H	-1.57190000	-2.04882000	1.78154800
S	-3.79932700	0.40235500	-0.40736300
O	-2.69580100	0.21402800	-1.37218400
O	-4.80816100	1.43358700	-0.67250700
C	-4.60864400	-1.17939100	-0.20094900
C	-4.41445300	-2.15792100	-1.17640600
C	-5.47554700	-1.38916200	0.87355500
C	-5.09285300	-3.36996600	-1.06160300
H	-3.73099800	-1.96757900	-1.99627600
C	-6.14040300	-2.60774000	0.97252300
H	-5.60820700	-0.61476600	1.62059400
C	-5.96079400	-3.61531300	0.01102600
H	-4.94630700	-4.13707100	-1.81714000
H	-6.81228600	-2.78169100	1.80866200
C	-6.66511900	-4.94249000	0.15042500
H	-6.76390500	-5.44848100	-0.81381300
H	-6.10554600	-5.61017200	0.81700100
H	-7.66518200	-4.82104300	0.57710200
C	-2.20505700	6.61663100	-2.37824700
H	-2.95937300	5.82222100	-2.30260900
H	-2.68398600	7.58387200	-2.21863400
H	-1.76192500	6.59527800	-3.38257700
In	3.38716100	-1.60810000	-0.72237700
Cl	3.66655300	-0.59105200	-2.84613700
Cl	3.67926900	-3.93752900	-0.74805500
Cl	4.94034900	-0.42573600	0.61931200
C	1.16133800	3.07582100	-1.03825200
C	1.95355500	1.80736700	-0.83157800
H	1.98254700	1.21705000	-1.74883600
H	2.99381000	2.01189200	-0.56705700

B-int3-R

0 1

C	-1.84270600	0.47280500	-0.74508000
C	-0.71359700	-0.07458300	-1.38266900
O	0.31095100	0.52091300	-1.79760900
C	-1.78961100	1.84823000	-0.28695000
O	-1.06316500	2.75973800	-0.65793100
O	-0.80743500	-1.45279600	-1.54174400
O	-2.72254500	2.06821900	0.72366900
C	0.22950200	-2.10959800	-2.27513300

H	0.97568300	-2.50839200	-1.58549900
H	-0.24564600	-2.92587500	-2.82552500
H	0.72660900	-1.41924300	-2.95593200
C	-2.66890900	3.35155400	1.33551900
H	-3.58257800	3.43948300	1.92901100
H	-1.79116800	3.43053000	1.97985100
H	-2.62783500	4.15175300	0.59497000
C	-2.46429800	-4.01376900	0.71628100
C	-2.92203500	-3.80500900	-0.58941400
C	-2.28679800	-4.44563600	-1.64512800
C	-1.19255400	-5.27069400	-1.36530700
C	-0.73516800	-5.44559900	-0.05296000
C	-1.36982100	-4.81548300	1.02068300
C	-4.22795600	-2.63216900	0.90673600
C	-4.06511900	-2.82831500	-0.54938500
H	-2.61853000	-4.29483800	-2.66760100
H	-0.67842400	-5.77188300	-2.17902200
H	0.12658100	-6.07647100	0.13705000
H	-1.00905000	-4.94852300	2.03406900
H	-4.94180900	-1.96355700	1.37260400
N	-3.31235100	-3.27641100	1.59430500
C	-3.09073200	-0.79778600	4.12783800
C	-1.26730400	-1.26959500	2.61960100
C	-2.58696300	0.48648100	4.34711900
H	-4.00639700	-1.09758000	4.63225300
C	-0.77396900	0.01297200	2.79661200
H	-0.75378600	-1.91988100	1.91844300
C	-1.42628100	0.89095800	3.68059400
H	-3.11144700	1.15542400	5.01774500
H	0.08771600	0.36119000	2.23640200
O	-0.85185700	2.11471700	3.82726500
C	-5.34454100	-3.18136400	-1.35620000
H	-5.33838700	-4.22523500	-1.67876500
H	-6.23440500	-3.01919000	-0.74235900
C	-5.35244000	-2.17697100	-2.53206000
H	-4.68009500	-2.50398300	-3.33291100
H	-6.34099000	-2.00255000	-2.95358500
N	-4.79025600	-0.94175300	-1.96477400
C	-3.61059500	-1.37708800	-1.21839200
C	-2.90408000	-0.45497000	-0.20867100
H	-2.88681000	-1.68107600	-1.97379200
H	-2.42412000	-1.13776900	0.49835100
H	-3.63084200	0.10598800	0.37334600
S	-5.96800500	0.04483400	-1.19243800

O	-6.05495300	-0.27388600	0.25013400
O	-7.18931900	-0.09038000	-1.99711800
C	-5.25706800	1.66190300	-1.39098900
C	-4.33724700	1.92457700	-2.40913900
C	-5.68749800	2.66447800	-0.52117100
C	-3.81499800	3.20767600	-2.52319900
H	-4.00419700	1.13041800	-3.06373500
C	-5.17651800	3.94992700	-0.67691100
H	-6.38848600	2.43604400	0.27441200
C	-4.21646500	4.23349400	-1.65814000
H	-3.05144900	3.40465100	-3.26902200
H	-5.49532800	4.73568900	0.00199700
C	-3.55591100	5.58516300	-1.74683100
H	-3.57894300	5.97583100	-2.76962400
H	-4.03165300	6.31757800	-1.08892200
H	-2.50216500	5.49282600	-1.45757500
C	-1.43719300	3.04674600	4.72575400
H	-1.45670400	2.65767100	5.75177300
H	-0.80386500	3.93362600	4.68482800
H	-2.45493500	3.31569300	4.41712600
Rh	4.33170800	0.24941200	0.53681200
O	3.52495900	-1.33619500	1.65020500
O	4.99702100	-1.09225100	-0.90940900
O	1.54471900	-1.25346100	0.54201500
O	3.01186400	-1.01209400	-2.00536900
C	2.36315700	-1.76085600	1.37889900
C	1.87780600	-3.00626900	2.11291000
C	2.93312800	-4.11427600	2.26702700
H	3.83496200	-3.67384400	2.70589800
H	2.55383700	-4.84968100	2.98819100
C	5.42458200	-3.68155500	-2.16482800
H	5.88882100	-4.26994200	-2.96569900
H	6.23866500	-3.28017200	-1.55105300
C	4.68147600	-2.49976000	-2.81361900
C	4.19543500	-1.44526200	-1.82862100
Rh	2.20545500	0.33543100	-0.64080800
O	2.99623300	1.87008700	-1.76243300
O	1.56128500	1.63833700	0.84325000
O	4.96059000	1.83302900	-0.62638800
O	3.58706400	1.64020600	1.86589600
C	4.15288700	2.31642700	-1.48525000
C	4.61649500	3.54469200	-2.25429500
C	3.51016100	4.58143000	-2.52535700
H	2.65997300	4.06441200	-2.98310500

H	3.89132800	5.29523500	-3.26630800
C	1.03497500	4.23271800	1.92742700
H	0.53684800	4.81981500	2.71099900
H	0.25914200	3.70127300	1.36929800
C	1.94200000	3.19387400	2.61196900
C	2.40067300	2.07021700	1.69850400
H	5.45562400	3.98374100	-1.70589900
H	5.01344900	3.17895000	-3.21032200
H	5.35794700	-1.98937400	-3.51066400
H	3.81784400	-2.84196300	-3.39169200
H	1.54059800	-2.67552700	3.10355900
H	1.00013500	-3.38064100	1.57726800
H	2.83512200	3.65441600	3.04551500
H	1.38659900	2.71483400	3.42547600
C	3.02537300	5.35616500	-1.28538200
H	3.86276000	5.92663000	-0.85649100
H	2.28387500	6.09824200	-1.61278300
C	2.40528300	4.45585400	-0.21270800
H	1.66166900	3.79578100	-0.66999500
H	3.19309200	3.80617800	0.18205900
C	1.76069400	5.19038400	0.96591100
H	1.02996100	5.91826200	0.58630600
H	2.52054500	5.76917300	1.51254000
C	4.55261300	-4.61357700	-1.30184900
H	3.75499600	-5.05238500	-1.91968800
H	5.17742700	-5.45314400	-0.96685700
C	3.94494800	-3.91349200	-0.08119000
H	4.71659500	-3.31169400	0.40811200
H	3.19038500	-3.20146200	-0.42804700
C	3.30375200	-4.83822400	0.95867500
H	3.99800500	-5.65470700	1.20013400
H	2.40721000	-5.31404800	0.53260000
C	-2.43701300	-1.69083900	3.28120400
C	-3.02968500	-3.05897200	3.03675900
H	-2.36946700	-3.87159900	3.34688400
H	-3.97298700	-3.17881300	3.57486900

B-int4-R
0 1

C	-1.54964100	-0.11606800	-2.28400100
C	-0.49290200	0.73633300	-1.57494500
O	0.06993000	0.32943700	-0.56409400
C	-0.77310900	-1.11493200	-3.15794900
O	0.40944100	-1.35090600	-3.06560100

O	-0.28753500	1.90028600	-2.15669400
O	-1.59646700	-1.73587200	-4.02708400
C	0.50306000	2.89042800	-1.45532300
H	1.55735500	2.71974500	-1.66566200
H	0.15596600	3.84777300	-1.84203700
H	0.32776500	2.81007500	-0.38284900
C	-1.02323000	-2.86027300	-4.71178900
H	-1.82058300	-3.25956600	-5.33797100
H	-0.16700000	-2.55053800	-5.31621200
H	-0.69766800	-3.60932600	-3.98520700
C	-3.11021600	-3.04192200	-1.75875800
C	-4.16446900	-2.15358800	-2.04473100
C	-5.31471300	-2.60315700	-2.67569000
C	-5.42656200	-3.95912300	-3.01736200
C	-4.38897200	-4.84203400	-2.70534800
C	-3.22009300	-4.39841100	-2.07589100
C	-2.31789400	-0.94068800	-1.18012500
C	-3.83463600	-0.79653200	-1.45819400
H	-6.12767800	-1.91392700	-2.89304800
H	-6.31971900	-4.32324500	-3.51555000
H	-4.48331800	-5.89332000	-2.96365400
H	-2.41226500	-5.08978700	-1.85606300
H	-2.05240600	-0.52102800	-0.21660200
N	-2.01278200	-2.37275000	-1.21233100
C	-3.02183500	-3.36571500	1.64125200
C	-1.52354300	-1.51930800	2.01458400
C	-3.75781300	-3.01717500	2.77628800
H	-3.34177600	-4.21699100	1.04598800
C	-2.24904000	-1.15699300	3.14532400
H	-0.65216600	-0.93721400	1.72996200
C	-3.37361500	-1.89993900	3.53038200
H	-4.61747600	-3.61404400	3.05665800
H	-1.95951900	-0.30158900	3.74710300
O	-4.02336900	-1.45731600	4.64949200
C	-4.68744800	-0.46987500	-0.19366800
H	-5.35728900	-1.29030500	0.07118300
H	-4.03629400	-0.27666500	0.66205500
C	-5.44553600	0.80429800	-0.58534800
H	-6.34936200	0.56330200	-1.15789700
H	-5.75094000	1.42662400	0.25572400
N	-4.55923900	1.54530500	-1.52106200
C	-3.98895000	0.44752000	-2.38180700
C	-2.63458000	0.66923300	-3.08136000
H	-4.77227500	0.27657300	-3.12715200

H	-2.37889900	1.71984200	-3.19398700
H	-2.71351700	0.24349200	-4.08055800
S	-3.44672800	2.60962700	-0.77433600
O	-2.92529800	3.49046600	-1.82672300
O	-2.49051600	1.88564900	0.09014500
C	-4.51524700	3.57702700	0.28403600
C	-4.43439600	3.42047500	1.66695600
C	-5.37288300	4.51469700	-0.29618000
C	-5.24117800	4.21585000	2.48083000
H	-3.74594300	2.69466200	2.08660900
C	-6.17151500	5.29559300	0.53282600
H	-5.39833500	4.63066100	-1.37423500
C	-6.12007800	5.15785100	1.93001500
H	-5.18234800	4.10522300	3.56004500
H	-6.84021700	6.03087400	0.09312400
C	-7.00796200	6.00295100	2.81053600
H	-6.67971800	5.98196100	3.85306600
H	-7.02011100	7.04566900	2.47739900
H	-8.04314000	5.64188500	2.78027400
C	-5.17280100	-2.16707000	5.07988300
H	-4.93691300	-3.20823100	5.33848000
H	-5.53191500	-1.65063400	5.97192700
H	-5.96413500	-2.16164000	4.31759800
Rh	4.57120600	-0.16916600	0.75759100
O	5.06418700	0.54558100	-1.13130200
O	4.52333800	1.76720900	1.52474400
O	2.88098300	0.82427700	-1.67019900
O	2.36045600	2.03877000	0.90068500
C	4.13040300	0.91304300	-1.90888700
C	4.52002000	1.52541700	-3.24366000
C	5.64945900	2.56919400	-3.15846000
H	6.50773900	2.10800700	-2.65672800
H	5.96642500	2.80301900	-4.18190300
C	4.74171200	4.68260300	1.39466200
H	4.75159100	5.63928800	1.93064400
H	5.63230100	4.12943600	1.71350000
C	3.49643800	3.89997800	1.85309200
C	3.46166700	2.45134100	1.39353400
Rh	2.25022600	0.11148000	0.15854500
O	1.76888700	-0.59731200	2.06719700
O	2.28783700	-1.84198200	-0.54551500
O	3.95813100	-0.90902000	2.57317900
O	4.47773600	-2.07662000	-0.00406400
C	2.71283000	-0.99027300	2.82724300

C	2.34328100	-1.60936200	4.16614100
C	1.22405900	-2.66624300	4.11896200
H	0.33719800	-2.21688300	3.66546000
H	0.95596800	-2.90830400	5.15474200
C	2.07576300	-4.73636600	-0.47659200
H	2.07352000	-5.69254800	-1.01423200
H	1.20183800	-4.17691300	-0.82293200
C	3.33449000	-3.95996000	-0.90180500
C	3.36487200	-2.50959300	-0.44874200
H	3.25978800	-2.02004200	4.59918800
H	2.02008000	-0.78104300	4.80987300
H	3.46893100	3.88009100	2.94987500
H	2.57607300	4.38316500	1.51222900
H	4.84363300	0.69520300	-3.88388400
H	3.61793100	1.94952400	-3.69419900
H	4.24579900	-4.44168300	-0.53580200
H	3.39065500	-3.94084900	-1.99754500
C	1.56368500	-3.97004800	3.37379800
H	2.39452700	-4.48563100	3.87630200
H	0.69337400	-4.63615300	3.45180900
C	1.90220000	-3.74864300	1.89507500
H	1.17460000	-3.05405700	1.46926700
H	2.87198100	-3.24627700	1.83207500
C	1.95055400	-5.01601400	1.03414700
H	1.03399900	-5.59986600	1.19899700
H	2.78320900	-5.65614600	1.35938400
C	4.82642800	4.95429800	-0.11920500
H	3.95332200	5.54076900	-0.44042100
H	5.70520300	5.58688100	-0.30461600
C	4.93691000	3.67444700	-0.95550200
H	5.68666100	3.01747900	-0.50279100
H	3.98830900	3.13439100	-0.88905100
C	5.28118500	3.87822000	-2.43546700
H	6.13390300	4.56588200	-2.51887200
H	4.44257700	4.36571000	-2.95354300
C	-1.90386100	-2.62798900	1.24044000
C	-1.22916800	-2.90747500	-0.08697500
H	-0.24029700	-2.44516000	-0.13040300
H	-1.10039700	-3.98100400	-0.24874600

B-TS3-I

0 1

C	1.61630000	0.14648200	0.56799200
C	1.24190300	0.85917800	1.73998500

O	1.43804700	2.07775500	1.98184400
C	2.26085900	0.89044300	-0.44588300
O	2.59979600	2.11376000	-0.37560300
O	0.56251500	0.12965500	2.65915500
O	2.48294200	0.24885300	-1.61011000
C	-0.03970800	0.88809800	3.72697000
H	-0.73814200	1.62047000	3.31609500
H	-0.56870100	0.15303700	4.33215400
H	0.72313300	1.40540100	4.31186400
C	2.92840600	1.07368100	-2.70659400
H	2.90015500	0.42099200	-3.57928400
H	2.27438600	1.93938700	-2.83219800
H	3.94825600	1.42438300	-2.53121700
C	-2.55022000	-1.42072300	1.06000000
C	-1.88987900	-2.37780900	1.85428600
C	-2.03522700	-2.34805400	3.24138800
C	-2.83569400	-1.35135300	3.80003700
C	-3.45043900	-0.37942100	2.99362800
C	-3.31628500	-0.39232400	1.60605800
C	-1.45197300	-2.77691200	-0.35383400
C	-1.05863800	-3.19626800	0.95727900
H	-1.54076300	-3.08314400	3.86971800
H	-2.97571400	-1.31641600	4.87614500
H	-4.04286400	0.40267200	3.45690300
H	-3.78253000	0.36298300	0.98915900
H	-1.06432000	-3.14789500	-1.29277500
N	-2.25486900	-1.71714600	-0.29715300
C	-5.01333400	-1.41613200	-1.64520600
C	-4.37304900	0.90742300	-1.55139700
C	-6.35869500	-1.05603400	-1.73290600
H	-4.74796700	-2.47088300	-1.64101100
C	-5.70738400	1.28048600	-1.64376100
H	-3.60298200	1.66981000	-1.46085600
C	-6.70988600	0.30204500	-1.73358700
H	-7.11420000	-1.82920500	-1.80100900
H	-6.00145300	2.32450000	-1.64278900
O	-7.98418000	0.77112100	-1.81306200
C	-0.52658500	-4.57950400	1.27228800
H	-1.15150700	-5.07835700	2.01830300
H	-0.52904200	-5.19639100	0.36980100
C	0.93808400	-4.44365900	1.78126000
H	0.96195400	-4.11638300	2.82509400
H	1.51330800	-5.36270000	1.68724700
N	1.54332600	-3.36757400	0.97656400

C	0.71882600	-2.24544200	1.05123200
C	0.78149500	-1.04884400	0.13769300
H	0.56451700	-1.96342000	2.08795500
H	-0.23952900	-0.66067600	0.10669000
H	1.04460900	-1.34231300	-0.87567900
S	2.18078200	-3.91279600	-0.56020700
O	1.14912400	-3.76865800	-1.60216200
O	2.71989700	-5.24440200	-0.27559500
C	3.47773200	-2.73916800	-0.86881600
C	3.81865700	-2.51586900	-2.20422900
C	4.16782100	-2.12205500	0.17650600
C	4.87459300	-1.65602300	-2.48943000
H	3.25621100	-2.99135100	-3.00017600
C	5.19957400	-1.24340400	-0.13543300
H	3.88416800	-2.30087400	1.20623300
C	5.56648100	-0.99408200	-1.46611200
H	5.14776000	-1.47458600	-3.52473000
H	5.72108100	-0.73180800	0.66824200
C	6.65801100	-0.00151500	-1.77701000
H	7.54309000	-0.17637100	-1.15692700
H	6.96012300	-0.04640900	-2.82640500
H	6.31533600	1.01906300	-1.56910000
C	-9.04779600	-0.16614900	-1.90556300
H	-9.08842700	-0.82060200	-1.02479700
H	-9.96410800	0.42346400	-1.95676600
H	-8.96547300	-0.78406300	-2.80935900
In	1.08288600	3.46006700	0.29387500
Cl	1.25078400	5.25203600	1.80008500
Cl	-1.06723300	2.25531900	0.04136400
Cl	1.16339400	4.46435000	-1.88018600
C	-4.01106000	-0.44899000	-1.55106900
C	-2.55707700	-0.83476500	-1.45053800
H	-2.21808300	-1.38031500	-2.33674900
H	-1.93344800	0.05883900	-1.34082500

B-TS3-n
0 1

C	-2.24671200	0.34572300	1.39884800
C	-2.85648800	1.60219700	1.69573900
O	-3.98314400	1.85790500	2.10247000
C	-2.83857700	-0.84533900	1.92740600
O	-3.86653000	-0.97540600	2.58605200
O	-1.96891100	2.68304000	1.43998400
O	-2.06179700	-1.98376900	1.64297500

C	-2.53468800	3.96816300	1.67069600
H	-2.83559600	4.09097400	2.71584200
H	-1.75365300	4.69170600	1.41983900
H	-3.42055400	4.13939300	1.04878100
C	-2.55940000	-3.18513500	2.21447300
H	-1.84073100	-3.96573100	1.94994600
H	-2.64662800	-3.10623300	3.30331200
H	-3.54657000	-3.44121000	1.81696300
C	2.42957700	1.27615200	0.29185300
C	1.63057900	2.11720800	-0.50328900
C	1.63591200	3.49099000	-0.26620100
C	2.44675100	3.98495100	0.75626500
C	3.23383600	3.12583200	1.53956400
C	3.23845300	1.74809800	1.32141500
C	1.35824100	-0.05393200	-1.16390400
C	0.84134900	1.25972600	-1.41013000
H	1.01825400	4.15942400	-0.85810500
H	2.46686300	5.05179400	0.95464000
H	3.85108900	3.53934900	2.33033500
H	3.85127300	1.08094000	1.91516100
H	1.02554900	-0.97098700	-1.63182200
N	2.21964700	-0.05197000	-0.15101300
C	4.96653400	-1.13225200	-0.70372600
C	4.98389300	-1.57229500	1.66072300
C	6.35872100	-1.20610500	-0.74052400
H	4.42588900	-0.92061500	-1.62256700
C	6.36983400	-1.65528700	1.64162100
H	4.45486600	-1.71344400	2.59991500
C	7.06870700	-1.47147500	0.43917700
H	6.87399900	-1.05730900	-1.68119200
H	6.93683100	-1.85624200	2.54396100
O	8.42241000	-1.56568100	0.52537000
C	0.36792700	1.68144500	-2.79969000
H	0.91857400	2.55996800	-3.14711400
H	0.55133600	0.87338900	-3.51296700
C	-1.16100200	1.94932200	-2.73484100
H	-1.36988300	2.95720000	-2.36274600
H	-1.65852200	1.81738000	-3.69314100
N	-1.68246700	1.00251100	-1.73977800
C	-0.89045000	1.13524900	-0.58699000
C	-0.94045600	0.24307200	0.63652900
H	-0.90748500	2.17936900	-0.28546300
H	-0.07448300	0.55621500	1.24163000
H	-0.78941500	-0.79692600	0.35362400

S	-2.12595100	-0.55958300	-2.38021000
O	-1.04287700	-1.52868600	-2.11974200
O	-2.49006000	-0.28983200	-3.77700800
C	-3.53592800	-0.97973300	-1.39862100
C	-3.79311200	-2.33439700	-1.18981800
C	-4.33945900	0.01649800	-0.83591100
C	-4.88162200	-2.68847800	-0.39766700
H	-3.13749500	-3.08935100	-1.60864800
C	-5.39478300	-0.36038900	-0.01727900
H	-4.10487600	1.06285500	-0.97834400
C	-5.66600900	-1.71238500	0.23146800
H	-5.08786200	-3.73948700	-0.21715700
H	-5.94685700	0.40863400	0.51072600
C	-6.69102900	-2.09220100	1.26303400
H	-7.56701500	-1.43777400	1.22745800
H	-7.02255700	-3.12903600	1.15423400
H	-6.22125800	-1.97211800	2.24631000
C	9.19134600	-1.39779700	-0.65938100
H	9.05331500	-0.39869900	-1.09238000
H	10.23286300	-1.51853300	-0.35974600
H	8.94171600	-2.15550700	-1.41305200
C	4.26198200	-1.31202300	0.48808300
C	2.75155500	-1.24943100	0.51423700
H	2.30039900	-2.10792700	0.00785400
H	2.37795100	-1.24831800	1.54303800

B-TS3-R

0 1

C	-1.85658200	0.48911300	-0.67113500
C	-0.75360700	-0.08049100	-1.33131100
O	0.27900200	0.49055000	-1.75959100
C	-1.77403700	1.85510100	-0.19724600
O	-1.03545600	2.75839500	-0.56707800
O	-0.88933000	-1.45548500	-1.49841300
O	-2.68611200	2.07730200	0.83185900
C	0.13581200	-2.14408600	-2.22028900
H	0.88247000	-2.53110700	-1.52475400
H	-0.35520900	-2.97067200	-2.73985500
H	0.63505500	-1.47851400	-2.92418200
C	-2.57546600	3.33492400	1.48752500
H	-3.46535100	3.42400700	2.11615200
H	-1.67458600	3.36602100	2.10337500
H	-2.53690300	4.16282200	0.77775200
C	-2.63646100	-3.93802900	0.70215800

C	-3.17934500	-3.84780100	-0.59078000
C	-2.56231900	-4.52255800	-1.64268600
C	-1.40853200	-5.26045300	-1.37474500
C	-0.86627200	-5.31530400	-0.08111000
C	-1.47487600	-4.65269800	0.98491800
C	-4.47557700	-2.66234400	0.87690100
C	-4.33525100	-2.93034300	-0.52747100
H	-2.96413500	-4.46895100	-2.65002900
H	-0.91209200	-5.79079200	-2.18108600
H	0.04290600	-5.88018500	0.09586200
H	-1.04801700	-4.69213700	1.98052600
H	-5.21264800	-2.01796300	1.33911800
N	-3.47657800	-3.20144900	1.57105800
C	-3.10490000	-0.70174500	4.11190900
C	-1.30643400	-1.26047100	2.60688300
C	-2.51705800	0.53940900	4.36835300
H	-4.04240100	-0.95107300	4.60365500
C	-0.72969900	-0.01818200	2.81810600
H	-0.82871500	-1.92722300	1.89612000
C	-1.32572200	0.87899400	3.72143800
H	-3.00050900	1.22546700	5.05259600
H	0.15747900	0.28415300	2.27157200
O	-0.66881900	2.05750900	3.90425600
C	-5.53901300	-3.04224400	-1.45486500
H	-5.63210500	-4.05625100	-1.85378600
H	-6.45600100	-2.81429600	-0.90473500
C	-5.36488100	-1.99354200	-2.58440800
H	-4.64413400	-2.33538400	-3.33415600
H	-6.29750400	-1.73544400	-3.08241500
N	-4.78377600	-0.81835400	-1.92107800
C	-3.65869300	-1.23966500	-1.19370900
C	-2.93869600	-0.42745800	-0.13365000
H	-2.94934700	-1.70828000	-1.87003400
H	-2.46736700	-1.17033000	0.50962000
H	-3.63814400	0.12591900	0.48687500
S	-5.95606100	0.25127200	-1.20457300
O	-6.10434200	-0.05809000	0.22944900
O	-7.13661100	0.14480500	-2.06879100
C	-5.14739800	1.81816500	-1.40309900
C	-4.22116100	2.02038300	-2.43038500
C	-5.49598700	2.84171900	-0.52180500
C	-3.60265100	3.25949100	-2.53597700
H	-3.95121500	1.21181400	-3.09667000
C	-4.88850500	4.08531200	-0.67057700

H	-6.20240300	2.65831500	0.28038200
C	-3.91032300	4.30113600	-1.65098700
H	-2.83010800	3.40394400	-3.28430600
H	-5.13875900	4.88493300	0.02044900
C	-3.11961000	5.58120100	-1.70586700
H	-3.03680300	5.96046900	-2.72948000
H	-3.55919300	6.36368500	-1.08126400
H	-2.10277400	5.37833500	-1.34784800
C	-1.18616900	2.99475400	4.83743800
H	-1.23463100	2.57024700	5.84838100
H	-0.49108500	3.83530600	4.82849000
H	-2.18207800	3.34793300	4.54213400
Rh	4.34123100	0.17497500	0.49420300
O	3.52766400	-1.37901600	1.64383800
O	4.95153700	-1.19902500	-0.94592800
O	1.53136400	-1.27921600	0.56660200
O	2.94863900	-1.09356400	-2.00691300
C	2.35479200	-1.78830900	1.39799400
C	1.86093100	-3.01385600	2.15802400
C	2.90414000	-4.13179500	2.32197300
H	3.81698200	-3.69362100	2.73976400
H	2.52546900	-4.84760900	3.06265000
C	5.31455300	-3.81169800	-2.16527300
H	5.75386200	-4.41932900	-2.96583800
H	6.14670600	-3.40815900	-1.57774300
C	4.56924100	-2.63340800	-2.81795800
C	4.12516200	-1.55069700	-1.84349700
Rh	2.19465500	0.28520500	-0.64318100
O	2.98999600	1.79558300	-1.79643800
O	1.60421300	1.61630000	0.83766800
O	4.97518100	1.73517200	-0.69866300
O	3.64909800	1.59311300	1.82126400
C	4.15874500	2.22523500	-1.54516000
C	4.62693400	3.44035600	-2.33254300
C	3.53061500	4.48915900	-2.59786000
H	2.66572900	3.97829300	-3.03450500
H	3.90866300	5.18836400	-3.35420600
C	1.13745400	4.22781300	1.90579800
H	0.66444400	4.82940900	2.69398800
H	0.34286500	3.70070300	1.37030800
C	2.04846600	3.18559100	2.57986100
C	2.46747200	2.04287600	1.67099400
H	5.47969900	3.87337400	-1.80061900
H	5.00548600	3.06047800	-3.29052300

H	5.23222700	-2.14741800	-3.54486500
H	3.68507500	-2.97537200	-3.36434200
H	1.53470000	-2.66120400	3.14473000
H	0.97442200	-3.38456000	1.63552200
H	2.95925500	3.63968700	2.98246100
H	1.51087100	2.72278800	3.41406400
C	3.07772900	5.28642900	-1.35993500
H	3.92976900	5.85223000	-0.95429700
H	2.33966200	6.03347200	-1.68408100
C	2.46673600	4.40853200	-0.26386900
H	1.70651800	3.75157000	-0.69784900
H	3.25380700	3.75405200	0.12444200
C	1.85360400	5.16553900	0.91733200
H	1.12326000	5.89660900	0.54272500
H	2.63037700	5.74310300	1.44097100
C	4.45308400	-4.71898000	-1.26598500
H	3.63946200	-5.16336000	-1.85841800
H	5.07797600	-5.55692500	-0.92716800
C	3.87619700	-3.98955000	-0.04776200
H	4.66255000	-3.38445100	0.41314300
H	3.12052100	-3.27852600	-0.39473700
C	3.24868900	-4.88683000	1.02389300
H	3.94273200	-5.70155000	1.27175000
H	2.34280500	-5.36841900	0.62466300
C	-2.50903300	-1.61715300	3.24646200
C	-3.18210300	-2.94815200	2.99876400
H	-2.57518000	-3.79239700	3.33669600
H	-4.13198400	-3.00079900	3.53651600

B-TS4-I

0 1

C	-0.87338000	-1.23633900	0.41422000
C	-0.69300300	-1.65637500	-0.95563700
O	0.33788300	-2.18119200	-1.42684600
C	0.01826000	-1.85388100	1.36931700
O	1.14187400	-2.33606100	1.12353300
O	-1.71340000	-1.38805100	-1.77512300
O	-0.42584000	-1.83221900	2.63048800
C	-1.50580700	-1.70266200	-3.17029000
H	-1.39882300	-2.78123800	-3.30312200
H	-2.39505900	-1.33276500	-3.67960400
H	-0.60660400	-1.20964700	-3.54455100
C	0.49673600	-2.32475500	3.62962100
H	-0.01329300	-2.18131200	4.58114200

H	0.71328000	-3.38038800	3.45485700
H	1.42518900	-1.75265900	3.59363600
C	1.64408500	0.81215400	1.80281800
C	0.43264500	1.11268000	2.44073400
C	0.39508500	1.25584400	3.82052800
C	1.58369300	1.09016400	4.54458800
C	2.78172500	0.78212400	3.89032700
C	2.83279900	0.63207800	2.49938300
C	0.09200500	0.77982600	0.16762800
C	-0.63167700	1.32223000	1.37886100
H	-0.53433600	1.49025200	4.33175300
H	1.57321900	1.19416000	5.62509000
H	3.68929500	0.64069000	4.46843300
H	3.74633000	0.34026700	1.99640800
H	-0.27211600	0.94552000	-0.83832300
N	1.40704400	0.70901700	0.40404700
C	1.95277300	3.00874000	-1.81990200
C	3.72430600	2.98195600	-0.19268300
C	2.13885900	4.37769900	-2.02231600
H	1.18785400	2.49297700	-2.39591000
C	3.92415800	4.34281200	-0.38137000
H	4.34675200	2.44388600	0.51651400
C	3.13052400	5.05176000	-1.29676800
H	1.52074200	4.89737900	-2.74418100
H	4.69220600	4.88291900	0.16196400
O	3.40474000	6.38088300	-1.40813800
C	-0.94752700	2.85549900	1.22458800
H	-0.22130800	3.46971100	1.75902800
H	-0.93408400	3.14888400	0.17162700
C	-2.37665600	2.96649900	1.77970100
H	-2.37841000	2.94219100	2.87524800
H	-2.91891000	3.85245400	1.45295400
N	-3.05051200	1.72715300	1.31924300
C	-2.03476900	0.66795500	1.61519800
C	-2.19934100	-0.70121200	0.90066600
H	-2.13352300	0.50799600	2.69269700
H	-2.85611200	-0.61782700	0.04461700
H	-2.66945300	-1.40293700	1.59378800
S	-3.59981400	1.90757000	-0.30863700
O	-2.54827300	1.53511500	-1.27593700
O	-4.19183400	3.24839700	-0.37456900
C	-4.90341100	0.69148600	-0.40115700
C	-4.92739300	-0.18679900	-1.48387800
C	-5.91456600	0.68805400	0.56281300

C	-5.98010300	-1.09436600	-1.59067100
H	-4.12102300	-0.16753000	-2.20753600
C	-6.95409200	-0.22760300	0.43937300
H	-5.87453300	1.38164100	1.39524600
C	-7.00359000	-1.13181300	-0.63474700
H	-6.00447600	-1.78578700	-2.42822400
H	-7.74285900	-0.24297800	1.18662300
C	-8.12336500	-2.13812400	-0.73385500
H	-8.22100800	-2.53352600	-1.74834100
H	-7.94111200	-2.98717700	-0.06390700
H	-9.08208800	-1.69692200	-0.44444100
C	2.65563400	7.14841300	-2.34008100
H	1.58554600	7.15367400	-2.09370000
H	3.04390500	8.16570800	-2.27306800
H	2.78577200	6.77888700	-3.36559700
In	2.28752500	-2.85064700	-0.67328700
Cl	3.03558900	-1.96924600	-2.76831800
Cl	1.87246600	-5.15036200	-0.62742400
Cl	4.15345100	-2.12951500	0.66322600
C	2.73357400	2.29358800	-0.90845000
C	2.45109600	0.83558700	-0.64200500
H	2.08617500	0.32244200	-1.53351400
H	3.33298500	0.28927700	-0.30521800

C-int3-I

0 1

C	0.53988200	-1.04119300	-1.28089100
C	1.78348900	-0.44520300	-1.56767400
O	2.93265400	-0.84862700	-1.19850700
C	0.57466400	-2.30531700	-0.62672700
O	1.54440200	-2.81068700	-0.00202600
O	1.73884400	0.67208000	-2.33439200
O	-0.57140300	-3.00229900	-0.70224000
C	2.98313200	1.09924500	-2.91957000
H	3.25650300	0.42698800	-3.73887800
H	2.79909800	2.10431300	-3.29586500
H	3.78147900	1.10932900	-2.18083000
C	-0.60283300	-4.29386500	-0.06656000
H	0.08432900	-4.98163100	-0.56503600
H	-0.31204400	-4.21370900	0.98409300
H	-1.63636300	-4.62326400	-0.16962400
C	-1.88821000	2.93423900	1.11043000
C	-2.54128700	1.73196200	0.82710700
C	-3.91790200	1.64796400	1.00124000

C	-4.59983600	2.77986200	1.46423500
C	-3.92198000	3.96836600	1.75751600
C	-2.53739800	4.06755000	1.58370500
C	-0.26041700	1.55205700	0.38634000
C	-1.50285200	0.71995300	0.39013100
H	-4.45801900	0.73820900	0.77880200
H	-5.67571500	2.72748400	1.59729500
H	-4.47361600	4.82832900	2.12309800
H	-2.01035500	4.98618400	1.81388300
H	0.73963100	1.22242800	0.14199200
N	-0.49725000	2.75866000	0.81773700
C	2.81377000	2.84618200	0.88898200
C	2.05529000	4.38544600	-0.81042100
C	4.02864300	2.68997100	0.22607000
H	2.64626400	2.27834100	1.79857600
C	3.26929400	4.26206800	-1.46721700
H	1.29126900	5.04362600	-1.21802800
C	4.26135400	3.40274900	-0.95958300
H	4.75286700	1.98697500	0.61812200
H	3.47651000	4.80546500	-2.38299800
O	5.38718600	3.31078900	-1.70299900
C	-1.35612900	-0.43780200	1.44611800
H	-0.38279100	-0.90825300	1.33176900
H	-1.42630700	-0.05814700	2.46665700
C	-2.49126600	-1.39497400	1.08181800
H	-2.21603400	-2.43334000	1.27147600
H	-3.40891200	-1.17020200	1.64318300
N	-2.68420300	-1.15021000	-0.34645300
C	-1.88892600	-0.05227400	-0.92100400
C	-0.74127600	-0.49370700	-1.86875600
H	-2.55427600	0.58487600	-1.51475500
H	-0.48933500	0.38030000	-2.47382500
H	-1.20375400	-1.21769100	-2.54596700
S	-3.83435700	-1.98586000	-1.20093100
O	-3.90448000	-3.32843000	-0.61831000
O	-3.57691800	-1.73983100	-2.62153600
C	-5.35089100	-1.13278400	-0.77099000
C	-6.01222300	-1.46090400	0.41545100
C	-5.76554500	-0.04916500	-1.54953900
C	-7.08403600	-0.67279300	0.83401100
H	-5.69221000	-2.32409000	0.98878100
C	-6.84139700	0.72308500	-1.11786600
H	-5.24808400	0.17372900	-2.47628000
C	-7.51029700	0.43144400	0.08097800

H	-7.60011200	-0.92180100	1.75739700
H	-7.16797000	1.56710600	-1.71952900
C	-8.68768300	1.26342100	0.52880400
H	-8.79843900	1.25088100	1.61705700
H	-8.59028700	2.30397400	0.20469100
H	-9.62076200	0.87643500	0.10174700
C	6.43801600	2.44883000	-1.24497900
H	6.10240000	1.41052500	-1.15889900
H	7.22648600	2.52734100	-1.99407700
H	6.82199400	2.77972000	-0.27289400
In	3.35506600	-1.78199100	0.64649600
Cl	5.46938900	-0.61031400	0.61412600
Cl	4.06879900	-3.87659300	1.45232200
Cl	2.03817600	-0.34950400	2.12350500
C	1.81189800	3.67957500	0.37945500
C	0.50483400	3.83818800	1.09222500
H	0.62378800	3.84525300	2.18083800
H	0.01264200	4.76846300	0.79949200

C-int3-R

0 1

C	-1.05806300	0.42742900	-1.63407200
C	-0.13510800	-0.55809300	-2.04130800
O	1.04321400	-0.42051000	-2.43835700
C	-0.80236100	1.80577900	-1.99112800
O	0.12295500	2.26674500	-2.64287600
O	-0.64785100	-1.85600900	-2.00110800
O	-1.81231100	2.64483600	-1.52571600
C	0.28500800	-2.88101700	-2.39065000
H	1.14393200	-2.90107700	-1.71778500
H	-0.27271700	-3.82007400	-2.33291100
H	0.63625900	-2.71790000	-3.41240000
C	-1.68491700	4.00164700	-1.94634800
H	-2.50254500	4.53855000	-1.46339300
H	-0.72370500	4.42533900	-1.64745600
H	-1.76575400	4.08537200	-3.03574500
C	-4.93764100	-3.24585100	-0.18779000
C	-5.85750800	-2.85134700	-1.22981000
C	-7.24035700	-3.18021800	-1.11876100
C	-7.65279900	-3.91718400	-0.03950300
C	-6.71991900	-4.33887500	0.96015400
C	-5.38008800	-4.02112500	0.90850300
C	-3.73937000	-2.02065400	-1.70415500
C	-5.12961300	-2.17537200	-2.19084900

H	-7.94023900	-2.85225200	-1.88004900
H	-8.69772800	-4.18624800	0.07280900
H	-7.08978800	-4.92108900	1.79903300
H	-4.69837600	-4.32108100	1.69553000
H	-2.94382100	-2.38896600	-2.35760300
N	-3.70860200	-2.77179700	-0.45394500
C	-0.61897700	-2.14958500	1.45758600
C	-2.82350800	-1.94963500	2.40607000
C	-0.07503100	-1.44026100	2.52762500
H	0.04130700	-2.43623200	0.64745200
C	-2.29503800	-1.23343800	3.47491400
H	-3.89610000	-2.09668800	2.34820000
C	-0.91328400	-0.98839200	3.55172700
H	0.98236100	-1.21126100	2.53106800
H	-2.93161300	-0.85991800	4.27076100
O	-0.48309300	-0.28161500	4.63480400
C	-5.62457200	-1.31470200	-3.28942600
H	-5.00181000	-1.44005800	-4.18130900
H	-6.65358600	-1.56246500	-3.55971100
C	-5.54043600	0.20450700	-2.82287900
H	-5.11504500	0.79694000	-3.63815200
H	-6.53544400	0.58139700	-2.59660800
N	-4.68603400	0.38540500	-1.64707600
C	-3.45673300	-0.39983500	-1.72576100
C	-2.27244000	0.05740100	-0.83441600
H	-3.13319300	-0.26789700	-2.76264600
H	-2.04095800	-0.71062900	-0.10601000
H	-2.59296600	0.92991800	-0.26963400
S	-5.47696300	0.54787600	-0.12830600
O	-5.10087300	-0.57110100	0.74484700
O	-6.89059100	0.79948700	-0.43886100
C	-4.76690600	2.02623600	0.56773000
C	-4.13133000	1.94562800	1.80653400
C	-4.92820300	3.24453600	-0.09314500
C	-3.65681900	3.11731400	2.39136600
H	-3.99996000	0.98336900	2.28839500
C	-4.43907900	4.40046100	0.50508600
H	-5.42442300	3.28529000	-1.05658800
C	-3.79319000	4.35584100	1.75087600
H	-3.16122700	3.06331500	3.35623900
H	-4.55755200	5.35474600	-0.00088600
C	-3.21472800	5.60883900	2.35798700
H	-3.81738900	6.48911900	2.11541300
H	-3.13950400	5.53173400	3.44623500

H	-2.20349200	5.78461200	1.97080400
C	0.92358200	-0.24520800	4.89192900
H	1.47638200	0.30412300	4.12293800
H	1.03547800	0.26573500	5.84926600
H	1.33342600	-1.26031300	4.96802600
Rh	2.55492900	0.03953000	-0.84156300
O	1.17289400	0.99704900	0.40736900
O	3.12170000	1.84887200	-1.63845500
O	2.70838300	1.27953800	2.05118100
O	4.58421200	2.24520000	0.05245200
C	1.55571300	1.44868900	1.52928300
C	0.55550800	2.24483000	2.35391700
C	-0.27301000	3.28062600	1.57793400
H	-0.79299900	2.78061800	0.75510600
H	-1.04702000	3.64424300	2.26063400
C	3.00161900	4.72153400	-2.01469900
H	3.32043000	5.57675600	-2.62327100
H	2.38208400	4.07844900	-2.64925800
C	4.26157400	3.93417900	-1.60519900
C	3.96686500	2.56403300	-1.01771700
Rh	4.20117900	0.41108000	0.90471900
O	5.60201300	-0.44063400	-0.37088900
O	3.71825400	-1.47509600	1.67387400
O	4.03232900	-0.88261200	-1.95046300
O	2.18559600	-1.82590600	0.04373000
C	5.21406900	-0.92723800	-1.48023400
C	6.25443600	-1.63848800	-2.33285200
C	7.19006900	-2.57541700	-1.54792200
H	7.63366600	-2.00678800	-0.72284700
H	8.01198000	-2.86777900	-2.21276100
C	3.94684700	-4.36142200	1.96109700
H	3.64900200	-5.26692800	2.50376900
H	4.48980900	-3.72744500	2.67099300
C	2.66779600	-3.62102000	1.53364000
C	2.88594200	-2.19488400	1.04373200
H	5.72657000	-2.17467000	-3.12684200
H	6.85091900	-0.85196000	-2.81240900
H	4.88289600	3.76290600	-2.49324000
H	4.86630800	4.48918500	-0.88178000
H	-0.13106600	1.51707200	2.80170900
H	1.10836100	2.71638500	3.17206000
H	2.13863200	-4.16717500	0.74638100
H	1.98157300	-3.54987300	2.38698400
C	6.52205600	-3.84547700	-0.98867600

H	6.12129100	-4.44886700	-1.81601200
H	7.29732500	-4.45882700	-0.50869300
C	5.41147200	-3.54448800	0.02387100
H	5.76820100	-2.79008500	0.73199500
H	4.57349100	-3.08238500	-0.50728200
C	4.88505800	-4.75291700	0.80406100
H	5.73067000	-5.31647200	1.22173300
H	4.36392200	-5.44183500	0.12316700
C	2.15110100	5.22892300	-0.83566500
H	2.74298800	5.91163200	-0.20839000
H	1.32437300	5.82770900	-1.24445100
C	1.57963100	4.08607600	0.00992800
H	1.16883500	3.33107000	-0.66503200
H	2.40085700	3.60354200	0.54856100
C	0.50883300	4.48573500	1.02960000
H	-0.21244700	5.16762100	0.55604000
H	0.96248000	5.04863900	1.85844000
C	-1.98604500	-2.42797200	1.38654400
C	-2.48846300	-3.25131500	0.21666100
H	-1.70821200	-3.28422500	-0.54177400
H	-2.69065000	-4.28345800	0.52660900

C-int4-R
0 1

C	-2.09935000	-0.16074200	-1.19345700
C	-2.06904000	-0.14649900	0.43073600
Rh	2.43550200	-0.85534400	-0.54200900
O	2.23363400	1.13353800	0.05068000
O	3.43797200	-0.27466400	-2.27752900
O	4.26725500	1.10550800	1.05160300
O	5.43648500	-0.28360700	-1.20518500
C	3.19771800	1.68321700	0.67831000
C	3.05684300	3.15978400	1.00782200
C	2.60648700	4.04697500	-0.16945400
H	1.65342300	3.67093300	-0.55770900
H	2.40155600	5.04306700	0.23725800
C	4.63886600	1.61154500	-4.16016700
H	5.11697800	1.78014500	-5.13233000
H	3.60988100	1.29527700	-4.36671200
C	5.37754700	0.44956000	-3.46976600
C	4.69550000	-0.07895500	-2.21895000
Rh	4.57137000	-0.88244200	0.56513700
O	4.75647800	-2.86520000	-0.01769300
O	3.58589600	-1.43862100	2.31148800

O	2.73532900	-2.82302600	-1.04847400
O	1.59395100	-1.47510300	1.23669500
C	3.79847200	-3.40707600	-0.65680900
C	3.91725300	-4.88506900	-0.98557300
C	4.36528800	-5.76701300	0.19583700
H	5.30889900	-5.36900900	0.58646900
H	4.58130500	-6.76650500	-0.20013700
C	2.33198400	-3.34610300	4.18737900
H	1.84258000	-3.51326500	5.15426000
H	3.37223400	-3.07400700	4.39997200
C	1.64085100	-2.14269200	3.51815400
C	2.33433400	-1.64479900	2.26243600
H	2.95480700	-5.21614300	-1.38551600
H	4.65596900	-4.96629800	-1.79296800
H	5.44878000	-0.39549300	-4.16626500
H	6.39752700	0.73493500	-3.19748800
H	2.31000200	3.23284300	1.80937500
H	4.01108900	3.50233800	1.41757900
H	0.60272300	-2.37329700	3.26262400
H	1.62840800	-1.30081900	4.22190300
C	3.35006800	-5.89077900	1.34732200
H	2.40825300	-6.31329400	0.96971500
H	3.74407100	-6.61590200	2.07253900
C	3.08154300	-4.55851300	2.05619800
H	4.03836400	-4.05959900	2.24378500
H	2.52792600	-3.90738100	1.37431200
C	2.30430900	-4.65412500	3.37476600
H	2.73648000	-5.45005000	3.99639000
H	1.26276100	-4.94721400	3.17917700
C	4.61856000	2.93696400	-3.37539700
H	5.64893200	3.27947200	-3.20439500
H	4.14119400	3.70015000	-4.00485400
C	3.87145900	2.84026800	-2.04095900
H	2.91169400	2.34055400	-2.20534900
H	4.43908500	2.19086400	-1.36903400
C	3.60986900	4.17193300	-1.32962400
H	3.20536500	4.89601600	-2.04985000
H	4.55605600	4.59499900	-0.96370700
C	-0.68643400	-0.20455700	-1.78482500
O	0.22919000	-0.81843100	-1.26388800
C	-2.70106400	-1.48880800	-1.72325700
O	-2.03433400	-2.47073900	-1.96697800
O	-0.59211100	0.42756600	-2.95127100
O	-4.03281100	-1.45068900	-1.88008100

C	0.70077500	0.38058300	-3.59856400
H	1.04581500	-0.65128300	-3.68374900
H	0.54844300	0.83323400	-4.57731400
H	1.42852700	0.94455700	-3.01621200
C	-4.64047100	-2.72419400	-2.17679600
H	-5.71064000	-2.53022400	-2.22327500
H	-4.26351900	-3.11683100	-3.12405500
H	-4.41667300	-3.43062900	-1.37395500
C	-3.83977400	-1.49898900	1.16921800
C	-2.43613500	-1.48004100	1.01926400
C	-1.66500100	-2.59859200	1.29357700
C	-2.30616800	-3.75358100	1.76501500
C	-3.69483200	-3.77042600	1.92294400
C	-4.48493900	-2.65050300	1.62408500
C	-3.30910900	0.69673400	0.79783700
H	-0.59282100	-2.56578400	1.12834000
H	-1.72350800	-4.64089000	1.99400000
H	-4.18156500	-4.67222600	2.28480700
H	-5.56262500	-2.68147400	1.73833500
H	-3.19869000	1.13165100	1.80349100
N	-4.40829500	-0.26437600	0.79069600
C	-6.97135800	-0.55385100	-0.69421600
C	-7.93326500	-1.02694900	1.45463100
C	-8.07976000	-1.11956900	-1.32806700
H	-6.14767400	-0.17418100	-1.28981200
C	-9.04490500	-1.59485700	0.84151600
H	-7.88067100	-1.00046900	2.54061100
C	-9.12595600	-1.64501000	-0.55680000
H	-8.11751700	-1.14247100	-2.41096400
H	-9.86112900	-2.01196900	1.42202600
O	-10.25241300	-2.22527900	-1.06452100
C	-0.87060900	0.63841200	0.98662600
H	-1.02804700	0.74799600	2.06433300
H	0.07316600	0.12447800	0.85209400
C	-0.78719700	2.02230900	0.32589300
H	-0.13048700	2.66852200	0.91256500
H	-0.33375800	1.95174000	-0.66873900
N	-2.12423300	2.67412000	0.24513300
C	-3.24616200	1.81453000	-0.24225900
C	-2.92861400	1.10687500	-1.57184300
H	-4.13671700	2.44110300	-0.29687100
H	-2.38737400	1.76447400	-2.25230300
H	-3.85746200	0.81144500	-2.05804900
S	-2.06264300	4.21917200	-0.44592200

O	-3.45339800	4.66397900	-0.60200200
O	-1.15297300	4.25327800	-1.60543700
C	-1.26896900	5.13480200	0.87440400
C	-1.71079800	4.99179500	2.19166600
C	-0.24356600	6.02222400	0.55102900
C	-1.09878600	5.73850000	3.19442900
H	-2.50570500	4.29068600	2.42085400
C	0.35152400	6.76899100	1.56709400
H	0.08468200	6.10844700	-0.47857200
C	-0.05966400	6.63530000	2.90008600
H	-1.43153800	5.62584000	4.22282500
H	1.15124200	7.46239400	1.32056200
C	0.62116200	7.41329500	3.99975400
H	-0.06246300	7.61779100	4.82886200
H	1.46931800	6.84889900	4.40671200
H	1.01012700	8.36775200	3.63346800
C	-10.38481000	-2.31317300	-2.47441800
H	-9.58018200	-2.91260700	-2.92163500
H	-11.34138400	-2.80501700	-2.65829600
H	-10.39384000	-1.32087700	-2.94501100
C	-6.87950800	-0.49800700	0.69801800
C	-5.68262500	0.15228300	1.36404100
H	-5.74636900	1.23978200	1.23666500
H	-5.70901100	-0.04028800	2.44984200

C-TS3-I

0 1

C	0.74524000	-1.21312300	-1.08638200
C	1.54580500	-2.30552000	-0.66755600
O	2.78148100	-2.30430000	-0.46678000
C	1.44294100	-0.03151200	-1.45947700
O	2.65744400	0.24217100	-1.24483300
O	0.88505100	-3.48535100	-0.46049100
O	0.71156700	0.86996500	-2.14014000
C	1.70968100	-4.58176400	-0.00835800
H	2.20853700	-4.32553800	0.92871300
H	1.02200900	-5.41763100	0.12836400
H	2.46703400	-4.82534200	-0.75587600
C	1.39755500	2.06132300	-2.57465200
H	0.61092700	2.76216600	-2.85292400
H	2.00966800	2.47013800	-1.77228000
H	2.03184900	1.82974000	-3.43471400
C	-2.45886700	-0.20336200	2.26096800
C	-3.36673200	-1.31292300	2.19365500

C	-4.69019000	-1.17475300	2.69014700
C	-5.04623400	0.01745200	3.27830100
C	-4.11099100	1.08998200	3.38525800
C	-2.82666000	1.00594600	2.88557100
C	-1.38059800	-1.88361600	1.21798900
C	-2.66229800	-2.38584800	1.63139000
H	-5.39216700	-2.00006700	2.62134600
H	-6.04578600	0.14984900	3.67927400
H	-4.42666200	2.00698000	3.87404900
H	-2.13030100	1.83348900	2.94718000
H	-0.45990700	-2.45626400	1.26291100
N	-1.27935300	-0.55085900	1.68028500
C	1.48746200	2.04280100	1.49426900
C	-0.72206700	2.13437400	0.54464700
C	1.79927400	3.27983600	0.93639900
H	2.25811000	1.51517500	2.04789600
C	-0.42678500	3.37942400	-0.00445300
H	-1.69264300	1.69163700	0.35091800
C	0.83418400	3.95900800	0.18337800
H	2.80484600	3.65913000	1.06047400
H	-1.14670200	3.90854000	-0.61594700
O	1.03576900	5.15703200	-0.43746500
C	-3.21787800	-3.64895500	1.09497000
H	-2.45865000	-4.43522100	1.10388600
H	-4.06673700	-3.99979100	1.68686000
C	-3.70996200	-3.41143800	-0.38665600
H	-3.56592200	-4.33626500	-0.95454500
H	-4.76900100	-3.16348000	-0.40687700
N	-2.96896100	-2.31198800	-1.01567700
C	-1.61690700	-2.22030700	-0.68421600
C	-0.72423200	-1.27603300	-1.44704200
H	-1.18848200	-3.21278100	-0.57730500
H	-1.12843800	-0.26551000	-1.37861700
H	-0.85464700	-1.57224500	-2.49927800
S	-3.82014100	-1.29946700	-2.14791700
O	-5.19864700	-1.78378200	-2.06425900
O	-3.06982500	-1.34266800	-3.40055500
C	-3.77755900	0.37167800	-1.52717000
C	-4.53852400	0.70221600	-0.40331400
C	-3.10773900	1.34655900	-2.26850200
C	-4.59835400	2.03235000	-0.00430700
H	-5.07810000	-0.06176900	0.14346700
C	-3.20359200	2.67621900	-1.86449300
H	-2.53435200	1.06411300	-3.14371700

C	-3.93591800	3.03899900	-0.72574000
H	-5.17326100	2.29458700	0.87813400
H	-2.69548500	3.44218700	-2.44235100
C	-3.97653100	4.46953500	-0.25334800
H	-3.80027400	5.17018900	-1.07388300
H	-4.93730000	4.71339900	0.20911400
H	-3.19390800	4.63922000	0.49618900
C	2.33162800	5.74166600	-0.34298600
H	3.10424500	5.08057100	-0.75496800
H	2.28907700	6.66143000	-0.92841600
H	2.58938800	5.98487300	0.69560800
In	4.07062100	-0.59845800	0.08903900
Cl	5.90613300	-1.74205500	-0.82135700
Cl	3.26487900	-1.10944900	2.31672900
Cl	4.87885600	1.63572500	0.48485900
C	0.23057600	1.45552800	1.31192100
C	0.00582000	0.10706700	1.96218300
H	0.79296200	-0.58104500	1.65435300
H	0.09033500	0.19820200	3.05230700

C-TS3-n

0 1

C	1.19124100	2.66246700	-0.55104100
C	1.80126200	3.58760300	0.35968800
O	1.35218700	4.60396000	0.86625400
C	-0.07948500	3.02583700	-1.13099400
O	-0.85243700	3.90745000	-0.78833800
O	3.11419600	3.17581600	0.71071200
O	-0.41214300	2.20100000	-2.22021700
C	3.79263400	4.08052100	1.58238500
H	3.25305200	4.20773500	2.52638900
H	4.77695500	3.64220600	1.76439600
H	3.90053600	5.06729500	1.12291100
C	-1.62549000	2.56773600	-2.87510000
H	-1.77872300	1.82161700	-3.65927700
H	-2.46967400	2.56910400	-2.18065100
H	-1.54687700	3.56486000	-3.31935100
C	0.02151600	-1.09667300	1.99964000
C	1.28324200	-1.74352600	2.22322100
C	1.33096800	-3.11201200	2.59393200
C	0.14433600	-3.78045700	2.79375600
C	-1.09964400	-3.11164300	2.62465100
C	-1.18586400	-1.79067200	2.22644200
C	1.65104700	0.40771300	1.53538200

C	2.28543300	-0.78284500	2.03085800
H	2.28651800	-3.60831800	2.73244400
H	0.14872400	-4.82432300	3.09042800
H	-2.01714700	-3.66421000	2.80455800
H	-2.14336800	-1.31117400	2.08203500
H	1.94448600	1.41677800	1.81463700
N	0.24529900	0.18922100	1.58656500
C	-3.21465000	1.20757800	2.06045900
C	-2.25991400	0.67446500	-0.07923100
C	-4.50197700	0.98433500	1.56203500
H	-3.09931100	1.50128600	3.10061700
C	-3.52947400	0.44802300	-0.58847100
H	-1.39813900	0.56457200	-0.72539500
C	-4.66134700	0.59511400	0.22793100
H	-5.35564300	1.11272900	2.21614600
H	-3.67539500	0.16166600	-1.62369600
O	-5.85790600	0.32659100	-0.37125800
C	3.74239500	-1.02195000	1.88555500
H	4.30707300	-0.13883000	2.19622800
H	4.07427500	-1.85936400	2.50488800
C	4.07541200	-1.35233400	0.38194300
H	5.09182200	-1.00737100	0.16312400
H	4.04003500	-2.42560900	0.20501300
N	3.11701700	-0.70275600	-0.52019900
C	2.62380400	0.54220700	-0.09371700
C	1.88490100	1.43691600	-1.06516300
H	3.42467100	1.08973400	0.39770400
H	1.16743800	0.85948700	-1.63634900
H	2.69185700	1.68979800	-1.77641400
S	2.60057300	-1.55528700	-1.93021600
O	3.32206900	-2.82886200	-1.85096800
O	2.77349300	-0.64993400	-3.06349900
C	0.85601900	-1.90470900	-1.76017500
C	0.45418800	-2.93067300	-0.90302600
C	-0.06174000	-1.23416500	-2.57070100
C	-0.89359900	-3.26464500	-0.84046300
H	1.18241100	-3.46282800	-0.30274800
C	-1.40547300	-1.59387100	-2.49920500
H	0.27216000	-0.45158900	-3.24160500
C	-1.84457800	-2.60186700	-1.63044700
H	-1.21175900	-4.05238600	-0.16548100
H	-2.12328500	-1.07898200	-3.13096800
C	-3.30781800	-2.94214900	-1.51670500
H	-3.83150900	-2.78980900	-2.46472900

H	-3.45668000	-3.97947800	-1.20396900
H	-3.78770700	-2.29295300	-0.77479900
C	-7.04374400	0.50815300	0.38912200
H	-7.14794900	1.54484200	0.73430100
H	-7.87099200	0.26720100	-0.27994600
H	-7.07562000	-0.16326000	1.25767400
C	-2.08430300	1.05664300	1.25723500
C	-0.70567100	1.30492500	1.81649900
H	-0.26612600	2.18966000	1.35277600
H	-0.76892600	1.47481200	2.89875600

C-TS3-R

0 1

C	-0.56783700	-1.23915900	1.77199500
C	0.07690400	-0.08283000	2.26468800
O	1.27494100	0.07500300	2.58194800
C	0.09508300	-2.51761600	1.92076000
O	1.02622300	-2.79402000	2.65735500
O	-0.76279500	1.02760700	2.33349700
O	-0.46864700	-3.49945500	1.11097500
C	-0.11643800	2.25281000	2.71895700
H	0.71120900	2.47685300	2.04694000
H	-0.88550100	3.02571300	2.64391700
H	0.25568600	2.18926300	3.74483000
C	0.16432500	-4.77155300	1.23946000
H	-0.35575800	-5.43065300	0.54528000
H	1.22674100	-4.71002200	0.98536500
H	0.08288500	-5.15538300	2.26161700
C	-4.84120300	1.49814200	-0.37438400
C	-5.77205100	0.92874900	0.55592400
C	-7.11559900	0.71572900	0.15948100
C	-7.49826100	1.10478200	-1.10494700
C	-6.56674600	1.69998600	-2.00071300
C	-5.24219300	1.90176400	-1.65978200
C	-3.68305800	0.93653300	1.47907700
C	-5.06512000	0.65690800	1.73989900
H	-7.82049800	0.25045100	0.84082300
H	-8.52158900	0.95433400	-1.43321000
H	-6.90941500	1.99863300	-2.98703900
H	-4.54021900	2.35377200	-2.34943300
H	-2.95943200	1.29981000	2.19880600
N	-3.60691300	1.52106500	0.19960500
C	-2.34890500	4.18590300	-1.85153500
C	-2.71594800	4.44868200	0.50740600

C	-2.38003100	5.57359400	-2.02602100
H	-2.18856600	3.55097900	-2.71996500
C	-2.75594000	5.82589100	0.35535500
H	-2.85342700	4.01630400	1.49498700
C	-2.58075900	6.40078900	-0.91532200
H	-2.24812100	5.98773900	-3.01803200
H	-2.91294100	6.48618700	1.20163900
O	-2.62825800	7.76078300	-0.95612100
C	-5.49394300	-0.20712200	2.86602400
H	-5.01284800	0.10761700	3.79674400
H	-6.57515000	-0.15550100	3.01545700
C	-5.09191700	-1.72099100	2.57888200
H	-4.69352600	-2.16497400	3.49536900
H	-5.96276100	-2.29100700	2.26263600
N	-4.06004600	-1.83134700	1.54407400
C	-3.01882300	-0.90662100	1.66367600
C	-1.73137000	-1.06958200	0.84510500
H	-2.79813900	-0.75885800	2.72034300
H	-1.59853900	-0.23467700	0.16022100
H	-1.81731300	-1.95815800	0.22690900
S	-4.58160400	-2.28801100	-0.06859300
O	-4.10270800	-1.27271700	-1.00850600
O	-6.00773800	-2.60085400	0.06344900
C	-3.69036400	-3.80154200	-0.37575800
C	-2.77863500	-3.84329500	-1.42860400
C	-3.97125300	-4.93374000	0.39230300
C	-2.13439600	-5.04592600	-1.71168300
H	-2.57898600	-2.94993300	-2.00831800
C	-3.31568800	-6.12317800	0.09540800
H	-4.69472100	-4.88600200	1.19920700
C	-2.39027500	-6.19856700	-0.95924100
H	-1.41638100	-5.08433000	-2.52490700
H	-3.52371800	-7.00970000	0.68789700
C	-1.65497700	-7.48466200	-1.24336200
H	-1.27575100	-7.51241000	-2.26827100
H	-0.79362500	-7.59101900	-0.57221800
H	-2.29739800	-8.35668200	-1.08872800
C	-2.40427500	8.40443300	-2.20275500
H	-3.17095400	8.13548300	-2.94137900
H	-2.45941600	9.47546400	-2.00311900
H	-1.41338400	8.16234200	-2.60891200
Rh	3.88768800	0.17810700	-1.34378500
O	4.88104000	-1.50814300	-0.65243100
O	2.47548400	-1.03330700	-2.29225900

O	3.62030700	-1.56153700	1.23552800
O	1.25673700	-1.14565500	-0.38615100
C	4.50642700	-2.02395700	0.45098600
C	5.16904300	-3.32041000	0.89147300
C	5.34253700	-4.36824400	-0.22308500
H	5.85818800	-3.89482500	-1.06647600
H	6.00718300	-5.15420100	0.15598900
C	1.19117000	-3.56340600	-3.03766500
H	0.41641400	-4.06116400	-3.63571700
H	1.89265800	-3.11228500	-3.74846500
C	0.52591000	-2.43727700	-2.22689500
C	1.50416400	-1.45759700	-1.59391100
Rh	2.53810400	0.08188100	0.67138300
O	1.47317500	1.73591100	-0.07353200
O	3.93439800	1.31729600	1.58067600
O	2.82167800	1.86643200	-1.89461200
O	5.18210000	1.40974900	-0.31404000
C	1.88823800	2.28999600	-1.14379200
C	1.20561400	3.58040200	-1.57217800
C	0.96016300	4.59217200	-0.43947100
H	0.41233800	4.08788600	0.36279800
H	0.29015100	5.36881600	-0.82510200
C	5.15371500	3.82518400	2.37539200
H	5.91869400	4.34615200	2.96403900
H	4.47949100	3.33749500	3.08827600
C	5.85612700	2.73126600	1.54976900
C	4.91335400	1.73724000	0.88750100
H	1.80226900	4.02079800	-2.37617200
H	0.23850000	3.29565600	-2.00383700
H	-0.12780700	-1.84981800	-2.88529500
H	-0.08649600	-2.84430000	-1.41787300
H	6.15744300	-3.04682100	1.28227400
H	4.58563200	-3.71789800	1.72665700
H	6.48746000	3.16458100	0.76846900
H	6.50911200	2.14684300	2.21038600
C	2.22630700	5.25149000	0.13768800
H	2.73665000	5.82297900	-0.65105700
H	1.91531000	5.98503000	0.89457200
C	3.20097100	4.24956300	0.76657600
H	2.64872000	3.57373600	1.42681300
H	3.61259400	3.62096700	-0.02832600
C	4.36438800	4.86227200	1.55397100
H	3.97622500	5.62322100	2.24552000
H	5.04465800	5.38856600	0.86899000

C	1.92865200	-4.61345700	-2.18617800
H	1.21370900	-5.11545600	-1.51875100
H	2.32208800	-5.38869400	-2.85827400
C	3.07909200	-4.01150100	-1.37041000
H	3.65350600	-3.34230000	-2.01860100
H	2.65768500	-3.38052800	-0.58157000
C	4.03954300	-5.01802300	-0.72656100
H	4.30488800	-5.79155300	-1.46072600
H	3.54034900	-5.53925000	0.10330800
C	-2.50419400	3.60483900	-0.59440000
C	-2.40591600	2.10648000	-0.40577000
H	-2.22953100	1.61048500	-1.36653100
H	-1.56393900	1.85614000	0.24281900

4-TS4-I
0 1

C	0.52005400	-0.99360500	-1.22947200
C	1.78237800	-0.35361400	-1.61455900
O	2.91518900	-0.66590000	-1.19238100
C	0.71771000	-2.41479500	-0.87256100
O	1.73463500	-2.90477600	-0.35969000
O	1.64695400	0.68443700	-2.42952000
O	-0.31453800	-3.19135100	-1.18623100
C	2.84937800	1.43342300	-2.74842500
H	3.46783800	0.84948800	-3.43289200
H	2.49249100	2.34729300	-3.21978400
H	3.40470700	1.65606300	-1.83920100
C	-0.22276400	-4.58034100	-0.79742000
H	0.62055900	-5.05899200	-1.29886600
H	-0.08544300	-4.65639100	0.28414500
H	-1.16936800	-5.01973900	-1.10792300
C	-0.86453900	1.36921900	2.10470400
C	-1.98508700	0.90679300	1.37944100
C	-3.27041700	1.18934400	1.80346300
C	-3.43479200	1.98410900	2.95174300
C	-2.32455900	2.42912400	3.66807700
C	-1.01513000	2.12197800	3.26030400
C	-0.02803000	0.48620800	0.20229900
C	-1.41783000	-0.07582700	0.37029000
H	-4.13145400	0.82404000	1.25527000
H	-4.43478900	2.24753900	3.28200600
H	-2.46598300	3.03116600	4.56047900
H	-0.16345400	2.47891800	3.82791700
H	0.20151200	1.14002300	-0.63592000

N	0.33929300	1.01671200	1.41583800
C	3.18220100	3.17541700	0.31232300
C	0.83016100	3.65496600	0.13682400
C	3.47387900	4.24404600	-0.54037000
H	3.99399100	2.56450200	0.69670900
C	1.10401200	4.70739800	-0.72802900
H	-0.20213200	3.44707600	0.40511800
C	2.43042800	5.01128500	-1.07301300
H	4.50767100	4.45381300	-0.78740400
H	0.30962100	5.31737800	-1.14532000
O	2.59163800	6.05915300	-1.92986400
C	-1.47602800	-1.45825000	1.12760300
H	-0.59531500	-2.06155200	0.93287600
H	-1.52084500	-1.31985000	2.20684400
C	-2.75318600	-2.08891900	0.57705800
H	-2.73655300	-3.17970900	0.59429900
H	-3.62477400	-1.74355000	1.15124600
N	-2.80279200	-1.60341700	-0.81269100
C	-1.98197700	-0.39610300	-1.01524700
C	-0.77451200	-0.64352700	-1.98430300
H	-2.59006200	0.42352500	-1.40602200
H	-0.60501800	0.26288600	-2.56592600
H	-1.05354700	-1.42803000	-2.68795600
S	-4.21836800	-1.76770500	-1.68209100
O	-4.71770900	-3.11296900	-1.39314000
O	-3.91499500	-1.29999100	-3.03720200
C	-5.33891400	-0.58715000	-0.93101400
C	-6.07701600	-0.96142700	0.19434900
C	-5.36876600	0.73020100	-1.40180000
C	-6.82767900	0.00344500	0.86721300
H	-6.07021500	-1.99502700	0.52254500
C	-6.12306200	1.67883500	-0.71717300
H	-4.82140300	0.99125900	-2.30101800
C	-6.85360400	1.33545200	0.43194600
H	-7.40113400	-0.28395100	1.74411700
H	-6.14724500	2.70367400	-1.07813800
C	-7.65735900	2.38286900	1.16289800
H	-8.04744600	2.00382500	2.11105900
H	-7.04887500	3.26873700	1.37415900
H	-8.51013500	2.71405500	0.55901500
C	3.91311000	6.42451000	-2.30315100
H	4.42616500	5.60833600	-2.82928500
H	3.81130700	7.27677700	-2.97654400
H	4.51284400	6.72073100	-1.43281700

In	3.49242100	-1.84710300	0.54777500
Cl	4.95438400	-0.00738800	1.03299000
Cl	4.82310200	-3.69264700	0.04772400
Cl	2.03711300	-1.71798000	2.43585400
C	1.86521600	2.86486800	0.66165900
C	1.60783400	1.72100500	1.62665100
H	2.41517100	0.98965600	1.57616600
H	1.60680400	2.07610500	2.66024500

InCl3
0 1

In	0.00000000	0.00000000	0.00000000
Cl	0.00000000	2.28666400	0.00000000
Cl	1.98030900	-1.14333200	0.00000000
Cl	-1.98030900	-1.14333200	0.00000000

int1-A
0 1

Rh	-1.99855700	-0.42467000	-0.32823500
O	-0.53266900	-0.56441200	1.22776000
O	-2.53405900	-2.40163800	-0.00333000
O	-2.05761900	-0.04904700	2.82444300
O	-4.01403800	-1.82889400	1.62400300
C	-0.90764000	-0.42496300	2.43936900
C	0.12077300	-0.67947600	3.53462400
C	0.84776100	-2.03794400	3.48583300
H	1.35371700	-2.14630400	2.52430600
H	1.63278800	-2.01138500	4.25188800
C	-2.55884300	-5.06578000	1.25085800
H	-2.92822100	-6.09846900	1.23496900
H	-1.89938800	-4.94916500	0.38484900
C	-3.76973500	-4.13120600	1.07404800
C	-3.41220100	-2.66645400	0.88091100
Rh	-3.59405800	0.18350600	1.43633300
O	-5.03709500	0.29094500	-0.04711700
O	-3.05752200	2.18831100	1.09554300
O	-3.52993700	-0.24595000	-1.66034300
O	-1.63829300	1.61678600	-0.58096300
C	-4.69124000	0.08067600	-1.25255300
C	-5.74611200	0.23207000	-2.33604800
C	-6.65864900	1.46073600	-2.17060300
H	-7.09189800	1.43813700	-1.16408700
H	-7.49042500	1.36031700	-2.87837500
C	-3.33394700	4.79369600	-0.22031100

H	-3.02560400	5.84620800	-0.24054700
H	-3.86566600	4.63489100	0.72480100
C	-2.06235100	3.92823700	-0.21052900
C	-2.28647400	2.46013100	0.12582200
H	-5.22845600	0.24016800	-3.29918700
H	-6.35650700	-0.67939700	-2.30202100
H	-4.33008300	-4.43487400	0.18091400
H	-4.44710100	-4.19892000	1.92997400
H	0.86501700	0.12383400	3.45060100
H	-0.38744500	-0.55339000	4.49462400
H	-1.54150200	3.97881100	-1.17090500
H	-1.36529800	4.30876700	0.54634400
C	-5.96971600	2.81940200	-2.39739200
H	-5.58031800	2.87044400	-3.42412000
H	-6.72955000	3.60905300	-2.31522500
C	-4.84045500	3.09368900	-1.39762500
H	-5.18932800	2.84662000	-0.38965300
H	-4.01746900	2.40404400	-1.60914900
C	-4.29058000	4.52324600	-1.39606800
H	-5.12327700	5.23725800	-1.33486700
H	-3.77710200	4.73102700	-2.34634600
C	-1.74044400	-4.83938600	2.53395200
H	-2.38837100	-4.95140800	3.41500000
H	-0.98366200	-5.63300300	2.60409800
C	-1.04453100	-3.47457400	2.56231000
H	-0.54011500	-3.31582300	1.60283900
H	-1.80552100	-2.69586400	2.64337400
C	-0.03391200	-3.27952900	3.69614900
H	0.62629600	-4.15576000	3.75241300
H	-0.55270800	-3.21864700	4.66325300
C	-0.53937200	-0.98825500	-2.00208900
C	-1.02647500	-2.30612500	-2.47640300
O	-1.95537800	-2.54327800	-3.21539500
C	-0.71907000	0.12355600	-2.95290300
O	-1.56171600	0.25582500	-3.81119200
O	-0.25138400	-3.31341900	-1.93709800
O	0.23868100	1.12495900	-2.74979800
C	-0.72010200	-4.63671500	-2.20723700
H	-0.62939900	-4.87649900	-3.27173300
H	-0.09220600	-5.30057700	-1.61071700
H	-1.76897300	-4.73498800	-1.91818900
C	-0.08321000	2.35309000	-3.41311700
H	0.66641100	3.07761500	-3.08706000
H	-0.05307200	2.23767700	-4.50010200

H	-1.08072600	2.68601400	-3.11908000
C	5.29402100	1.76996700	-1.15641700
C	5.62795500	0.56434400	-1.83838300
C	6.89748300	-0.00467200	-1.64142600
C	7.79731300	0.63130500	-0.79452500
C	7.45021100	1.82858100	-0.13405900
C	6.19778200	2.41032000	-0.30152800
C	3.55175100	1.20214500	-2.44810100
C	4.49819400	0.22323900	-2.66195300
H	7.17681700	-0.92182500	-2.15376900
H	8.78457700	0.20602100	-0.64026000
H	8.17550600	2.30665200	0.51792700
H	5.93150000	3.32480300	0.21707700
H	2.56043200	1.30649900	-2.86740400
N	4.01761000	2.13431700	-1.54378300
C	3.06495200	3.97978100	1.29059300
C	1.36571400	2.60614400	0.28689400
C	2.36018500	3.99515200	2.49016900
H	3.99749800	4.53510900	1.21971800
C	0.65650500	2.59259000	1.48969400
H	0.93185600	2.09315000	-0.56737000
C	1.13467100	3.31506700	2.59336400
H	2.71788500	4.55124900	3.35051900
H	-0.26929600	2.03962600	1.54421500
O	0.48586700	3.42819100	3.77890700
C	4.35951900	-0.97754400	-3.54631400
H	3.58865400	-0.80703600	-4.30503900
H	5.28752500	-1.15869600	-4.10191900
C	4.07208000	-2.28798200	-2.77507100
H	3.83869200	-3.11426900	-3.45230500
H	4.94643200	-2.57736500	-2.19465400
N	2.94861100	-2.15252100	-1.80761800
C	1.90860300	-1.42472700	-2.08471700
C	0.77477600	-1.03478200	-1.22599300
H	1.87900600	-1.03305200	-3.09950500
H	1.01203800	-0.02468700	-0.87344900
H	0.68466400	-1.67615900	-0.35924900
S	3.24265500	-2.91200000	-0.13218200
O	4.31085400	-3.86493600	-0.41471700
O	1.93944800	-3.33733000	0.35102200
C	3.87828500	-1.53626600	0.77981400
C	5.25787500	-1.49643200	1.01360200
C	3.01472900	-0.54919300	1.26854000
C	5.77234100	-0.45225700	1.77065000

H	5.90359200	-2.27025200	0.61657800
C	3.56283900	0.49393000	2.00677500
H	1.94402300	-0.58837800	1.10700800
C	4.93809600	0.55631000	2.27394000
H	6.84112600	-0.40428200	1.94850200
H	2.90808900	1.27436500	2.37520800
C	5.51018500	1.67811300	3.09977000
H	6.48751300	1.98311400	2.71554800
H	4.84615100	2.54535400	3.09994200
H	5.64625100	1.35904400	4.14022500
C	-0.88006600	2.99877500	3.85260500
H	-1.48549900	3.43622900	3.05086500
H	-0.97660000	1.91207500	3.80043400
H	-1.24416400	3.34745800	4.81976300
C	2.57672200	3.29647900	0.16748100
C	3.31275100	3.35830600	-1.15500400
H	2.61154900	3.58179800	-1.96395400
H	4.04619800	4.17108200	-1.13943700

int1-B

0 1

Rh	-2.00367000	-0.05926300	0.43548800
O	-2.55079900	1.00825200	-1.26944200
O	-3.42133100	0.94325000	1.55958000
O	-4.20197900	-0.46214200	-1.78876400
O	-5.09458000	-0.46533900	0.95019100
C	-3.57166100	0.63126500	-1.93192700
C	-4.08170200	1.57420600	-3.01677600
C	-3.88927600	3.07062100	-2.71265300
H	-2.87017400	3.21962500	-2.33670600
H	-3.97464700	3.63129300	-3.65154300
C	-5.35375400	2.95155400	2.16497500
H	-5.95151200	3.47096100	2.92391500
H	-4.29918500	3.11860600	2.40979100
C	-5.63189800	1.44187600	2.28084100
C	-4.63477200	0.56611700	1.53440800
Rh	-3.78979800	-1.64615700	-0.12810400
O	-3.29708400	-2.65314700	1.63024400
O	-2.36244200	-2.73215800	-1.20337200
O	-1.59882600	-1.21855500	2.09758900
O	-0.71390000	-1.26281400	-0.68190600
C	-2.31670400	-2.24815200	2.32517100
C	-1.93626100	-3.06472200	3.55142400
C	-1.88739900	-4.58363900	3.30599100

H	-2.85059000	-4.89447700	2.88596600
H	-1.78691700	-5.08095400	4.27830800
C	-0.27393400	-4.74379300	-1.52479800
H	0.37277900	-5.30877800	-2.20900900
H	-1.30375900	-5.04256000	-1.74956300
C	-0.12430000	-3.24722600	-1.85327800
C	-1.15590100	-2.34513700	-1.18965700
H	-0.97579100	-2.69078000	3.91855200
H	-2.69045500	-2.84865200	4.31886800
H	-5.57711900	1.14230100	3.33546600
H	-6.63473300	1.18526200	1.92637900
H	-3.55128900	1.30562100	-3.93877400
H	-5.13445200	1.33240400	-3.19122200
H	0.86885000	-2.87550600	-1.58933000
H	-0.25088800	-3.11144200	-2.93461200
C	-0.75293400	-5.05726900	2.37870200
H	0.22003900	-4.78577300	2.81374800
H	-0.77725200	-6.15514100	2.34107200
C	-0.86464100	-4.49692800	0.95568200
H	-1.89956200	-4.59582300	0.61346800
H	-0.65807400	-3.42318600	0.98546800
C	0.06527000	-5.14099900	-0.07647400
H	-0.00136700	-6.23482300	0.00172100
H	1.10813400	-4.87777800	0.14870600
C	-5.65968800	3.56178500	0.78321200
H	-6.73743000	3.49587900	0.57660300
H	-5.42190100	4.63435900	0.82179500
C	-4.86608900	2.90502800	-0.35177900
H	-3.82977600	2.78537100	-0.03351700
H	-5.24849200	1.88877700	-0.50208200
C	-4.89553100	3.64123700	-1.69448300
H	-4.66323800	4.70393400	-1.53670600
H	-5.90870500	3.61024300	-2.12000000
C	-0.30024500	1.40754500	0.94968300
C	-0.74406300	2.74483200	0.49056000
O	-0.29114100	3.29155500	-0.51433300
C	-0.09773300	1.12785800	2.39127800
O	0.87646200	0.50524100	2.82079500
O	-1.68073100	3.34557100	1.25286100
O	-1.06630100	1.57541400	3.20461700
C	-2.05580900	4.66205600	0.82760600
H	-2.44154800	4.65047800	-0.19444800
H	-2.83380500	4.97965600	1.52195100
H	-1.20053400	5.34340400	0.87051900

C	-0.99861200	1.10509100	4.55539000
H	-1.83674100	1.57667600	5.06915500
H	-1.10205900	0.01684900	4.57313500
H	-0.05183400	1.38347000	5.02695900
C	6.39933600	0.16676100	1.97769600
C	6.20310000	1.56767500	1.83695900
C	7.30489800	2.38821200	1.54319100
C	8.55505300	1.80332100	1.38305700
C	8.72602900	0.40914100	1.50949800
C	7.65462800	-0.42659100	1.80602600
C	4.21570200	0.59896400	2.29788200
C	4.79818000	1.81839700	2.04230800
H	7.18123500	3.46305400	1.43864700
H	9.41476700	2.42509200	1.15193700
H	9.71320900	-0.02117200	1.37015800
H	7.79017400	-1.50006500	1.89200400
H	3.18362900	0.34841400	2.51258900
N	5.16901600	-0.40521500	2.27509800
C	4.90751800	-1.26634000	-0.45598800
C	3.77225100	-3.25875100	0.25857000
C	4.63029700	-1.58034200	-1.77992500
H	5.45800200	-0.35694800	-0.24626800
C	3.47539400	-3.58268700	-1.06936200
H	3.42500300	-3.92303400	1.04695600
C	3.89682000	-2.73065600	-2.09668800
H	4.97709300	-0.93652600	-2.58129500
H	2.90763300	-4.47949100	-1.28372500
O	3.62331000	-2.91069300	-3.42538100
C	4.11997500	3.14726400	1.88017200
H	3.22840300	3.22991200	2.51015600
H	4.78547400	3.95359800	2.21076500
C	3.79128900	3.48130700	0.40897500
H	3.36974500	4.48435000	0.30688600
H	4.71114300	3.42949400	-0.17744300
N	2.82406500	2.54448700	-0.24584600
C	2.00940800	1.81706300	0.44819800
C	0.89042200	0.91444600	0.10455900
H	2.12092500	1.93186600	1.51894200
H	1.16177100	-0.09155600	0.43603400
H	0.63409600	0.87572300	-0.94262000
S	3.09108100	2.58498800	-2.08166100
O	4.36835300	1.88569600	-2.20593100
O	2.95906200	4.00774600	-2.36980700
C	1.80791500	1.65614500	-2.83841900

C	2.04680600	0.30792700	-3.12130800
C	0.58237100	2.27288900	-3.11097400
C	1.01450800	-0.44539200	-3.66216500
H	3.00534100	-0.13975900	-2.90577500
C	-0.43239900	1.49084900	-3.64668700
H	0.42012600	3.31307700	-2.86498200
C	-0.24528900	0.12301600	-3.90516500
H	1.19705700	-1.49121100	-3.88198500
H	-1.39438800	1.94808600	-3.84175700
C	-1.38722800	-0.72553800	-4.39974000
H	-2.01547500	-0.17991400	-5.10981800
H	-1.03003400	-1.63511000	-4.89027400
H	-2.02716900	-1.02856300	-3.56238100
C	3.03068800	-4.13806900	-3.82999800
H	3.63583100	-4.99897600	-3.51807400
H	2.01599000	-4.25063100	-3.43026600
H	2.98472300	-4.10552700	-4.91974000
C	4.48836600	-2.10521800	0.58740900
C	4.82410500	-1.80032300	2.04400800
H	3.97361600	-2.05011400	2.68679000
H	5.66368000	-2.41746200	2.38339100

int1-C
0 1

Rh	1.96033900	0.13958200	-0.23863900
O	1.15187400	0.91677700	1.54417900
O	2.37784800	2.07835400	-0.89516300
O	3.17468100	0.92571800	2.56947900
O	4.35889100	2.12245100	0.21482100
C	1.93272000	1.17408600	2.51977300
C	1.31723900	1.83044800	3.74749500
C	0.28460100	2.92752900	3.43929600
H	-0.47453400	2.50583700	2.77237800
H	-0.23098900	3.18182200	4.37303500
C	2.43770200	5.01254500	-0.68954700
H	2.64922200	5.97251100	-1.17641100
H	1.56588000	4.58070900	-1.19487600
C	3.65258600	4.09280100	-0.92114200
C	3.44704100	2.64492800	-0.49910400
Rh	4.12678000	0.17659700	0.88682300
O	4.96707700	-0.52111300	-0.87868100
O	3.72959600	-1.78150000	1.49362100
O	2.93143200	-0.61655400	-1.87722700
O	1.70459900	-1.78290100	0.47047000

C	4.19193900	-0.79066800	-1.84934300
C	4.80329200	-1.38562100	-3.10715300
C	5.79053800	-2.53881100	-2.84857000
H	6.55440500	-2.18894500	-2.14462900
H	6.30372300	-2.76029100	-3.79224400
C	3.69235000	-4.70034600	1.25553000
H	3.48856300	-5.66756000	1.73131700
H	4.53282400	-4.25132400	1.79693600
C	2.45369300	-3.81000900	1.45161300
C	2.65639200	-2.34188100	1.11461700
H	3.98111800	-1.69917600	-3.75631600
H	5.33079100	-0.56739600	-3.61376200
H	3.88661700	4.07432200	-1.99318100
H	4.53568600	4.47157600	-0.39928000
H	0.83050200	1.03159300	4.32137300
H	2.13618800	2.21314100	4.36323900
H	1.60729900	-4.16694100	0.85766900
H	2.13284300	-3.84169000	2.49804000
C	5.15381600	-3.83213100	-2.30665600
H	4.41997700	-4.21540100	-3.03018000
H	5.94005300	-4.59604800	-2.23132400
C	4.49160900	-3.64631300	-0.93644400
H	5.16263700	-3.06887600	-0.29237500
H	3.59569500	-3.03121000	-1.06370500
C	4.09860400	-4.93692300	-0.21054100
H	4.94471700	-5.63761600	-0.22635400
H	3.27725200	-5.43580700	-0.74625200
C	2.08032600	5.26197700	0.78731100
H	2.94211200	5.69409300	1.31442700
H	1.29405800	6.02961100	0.82785300
C	1.60766700	3.99019500	1.50444700
H	0.97238100	3.41187900	0.82627500
H	2.48376400	3.36280500	1.68805500
C	0.87345400	4.21494100	2.83094700
H	0.05170700	4.92837100	2.67749500
H	1.55418200	4.68310100	3.55586100
C	-0.12176500	-0.04073700	-1.32817500
C	-0.17306900	1.27763300	-1.98726700
O	-0.77180000	2.24379900	-1.51545000
C	0.04664800	-1.27252700	-2.14255300
O	0.54278800	-1.39403200	-3.24186900
O	0.52142800	1.35100700	-3.13448100
O	-0.46591100	-2.35663400	-1.46064200
C	0.66766900	2.66339100	-3.68424600

H	1.28992400	3.27085700	-3.02295300
H	1.16095800	2.52232100	-4.64644000
H	-0.30256100	3.15111800	-3.81682000
C	-0.08690700	-3.63562500	-1.97357300
H	-0.56809100	-4.36399200	-1.31875000
H	-0.41402700	-3.76285000	-3.00989500
H	1.00068600	-3.74809800	-1.93047600
C	-6.17475500	-2.74890300	-0.31171400
C	-6.20274200	-1.52830500	-1.04281800
C	-7.26812500	-0.63438400	-0.84331200
C	-8.25626000	-0.95800400	0.07673900
C	-8.20266300	-2.16511600	0.80308000
C	-7.16786700	-3.07532700	0.61903800
C	-4.35845200	-2.68576000	-1.62728200
C	-5.02311500	-1.50983500	-1.87166600
H	-7.32047500	0.29746200	-1.39888000
H	-9.08076100	-0.27206100	0.24539300
H	-8.98504800	-2.38946700	1.52187100
H	-7.13630500	-4.00224100	1.18255300
H	-3.40905100	-3.03220200	-2.01401600
N	-5.04597700	-3.45446600	-0.70712500
C	-2.20495000	-4.96345500	1.17689200
C	-3.22591200	-2.80290400	1.44020800
C	-1.13950600	-4.56403600	1.97449300
H	-2.20907700	-5.96860500	0.76034400
C	-2.15991800	-2.38226900	2.23836500
H	-4.03255700	-2.10430500	1.23708600
C	-1.10491900	-3.26488800	2.50375500
H	-0.31324300	-5.23291800	2.18908800
H	-2.15866100	-1.36845600	2.62060900
O	-0.01379600	-2.95945900	3.25699500
C	-4.53235800	-0.37755200	-2.71918200
H	-3.64940700	-0.68036400	-3.29248200
H	-5.28241800	-0.07315000	-3.46044800
C	-4.23329300	0.89844300	-1.90225900
H	-3.78827400	1.66926900	-2.53903100
H	-5.16220700	1.27715800	-1.48039600
N	-3.29251800	0.70884900	-0.76639200
C	-2.32998300	-0.18482500	-0.78715600
C	-1.03035900	-0.17237500	-0.09234000
H	-2.43898700	-0.95670700	-1.53657000
H	-0.82928100	-1.11928500	0.40200400
H	-0.90430900	0.64842200	0.59908300
S	-3.67742100	1.67670800	0.72076500

O	-2.79288300	1.16973200	1.76425700
O	-5.12909300	1.54382100	0.81382000
C	-3.26949700	3.34209900	0.28764500
C	-1.95862300	3.78884900	0.45206900
C	-4.28085700	4.17451300	-0.20275800
C	-1.65976700	5.10071200	0.10597100
H	-1.18979100	3.12380600	0.81585700
C	-3.95564800	5.48481300	-0.53992600
H	-5.29689100	3.80665300	-0.29070500
C	-2.64383100	5.96342100	-0.39708100
H	-0.64103300	5.45277600	0.21893900
H	-4.73132900	6.14742900	-0.91303400
C	-2.28653800	7.37170600	-0.80135200
H	-3.15273800	8.03771900	-0.75647000
H	-1.50135400	7.78296100	-0.16036400
H	-1.90867600	7.38960800	-1.83094100
C	0.20567600	-1.59170400	3.59437900
H	0.19124700	-0.95627500	2.70487600
H	-0.53595000	-1.23491300	4.32105300
H	1.19962100	-1.55125900	4.04384700
C	-3.26424900	-4.08968000	0.89619500
C	-4.42049000	-4.54933000	0.02249600
H	-4.07050300	-5.29686600	-0.69883700
H	-5.19896500	-5.03713300	0.61772000

int2

0 1

C	-1.18667500	-0.28424800	0.85406400
C	-1.78303400	-1.46718700	0.35632400
O	-2.99779700	-1.76936100	0.38350200
C	-2.03119700	0.68483700	1.45314100
O	-3.28757300	0.62341600	1.52412500
O	-0.91082800	-2.36124300	-0.19471600
O	-1.41344000	1.77256300	1.94945000
C	-1.53562700	-3.47833900	-0.86770800
H	-2.25839700	-3.11831200	-1.60335900
H	-0.71830000	-4.01789700	-1.34952500
H	-2.04866900	-4.12211400	-0.15011300
C	-2.28346000	2.80995400	2.45390600
H	-1.62031500	3.63725500	2.70681600
H	-3.01417700	3.10001700	1.69553000
H	-2.81532700	2.46105000	3.34180100
C	4.42260500	-1.72903800	-1.29906800
C	4.55139000	-2.10434600	0.07089700

C	5.75302800	-1.83449300	0.74973300
C	6.77810600	-1.18413100	0.07197600
C	6.62678300	-0.80680300	-1.27979100
C	5.45566500	-1.07547100	-1.98183200
C	2.50350900	-2.69186400	-0.66363000
C	3.30789700	-2.71567100	0.45590200
H	5.88053500	-2.12911500	1.78780500
H	7.71027100	-0.96692000	0.58400100
H	7.44582200	-0.30475400	-1.78609100
H	5.35349500	-0.78515600	-3.02247100
H	1.47971300	-3.01843500	-0.78072900
N	3.16784800	-2.12302100	-1.72772500
C	0.18025900	-0.85767400	-2.86140600
C	2.03085600	0.67459900	-2.74708500
C	-0.72730100	0.18572800	-2.65793200
H	-0.20951700	-1.86522600	-2.97830000
C	1.14542200	1.72526400	-2.55936200
H	3.10032700	0.87036200	-2.75573800
C	-0.24051500	1.49115200	-2.49469900
H	-1.78829300	-0.03170700	-2.61537100
H	1.49241800	2.74441800	-2.44147300
O	-1.00687000	2.58154300	-2.26251800
C	2.90961400	-3.14729900	1.83326100
H	1.96888000	-3.70648200	1.79951600
H	3.64893600	-3.82914600	2.27060000
C	2.80020000	-1.96401600	2.83172500
H	2.31631900	-2.27062000	3.76316500
H	3.79072100	-1.58754500	3.08166500
N	2.05020000	-0.80339700	2.27617800
C	1.07302300	-0.98008700	1.44205100
C	0.28723300	0.00247300	0.66117200
H	0.77548900	-2.01049300	1.27844000
H	0.58351100	-0.15811400	-0.38520100
H	0.50475300	1.03178600	0.91592600
S	2.59546400	0.88496700	2.85010400
O	3.78818000	0.55771600	3.62253400
O	1.39353900	1.45374400	3.43890100
C	3.04090500	1.76099600	1.37591200
C	4.19116100	1.38736300	0.67696400
C	2.27037300	2.86867400	1.00769400
C	4.55725400	2.13815400	-0.43499800
H	4.78962400	0.53898700	0.98582900
C	2.66932200	3.60932700	-0.09950400
H	1.38333900	3.13343000	1.57290200

C	3.80738900	3.25314500	-0.84002100
H	5.43970700	1.84341200	-0.99324100
H	2.07962800	4.47010600	-0.39979100
C	4.19187900	4.02185700	-2.07765500
H	5.27634300	4.04122900	-2.21700000
H	3.75596100	3.54929700	-2.96701700
H	3.82760100	5.05179400	-2.04341100
C	-2.39832900	2.38129500	-1.97515900
H	-2.52288000	1.75080600	-1.09186800
H	-2.81321000	3.36661600	-1.76913700
H	-2.93407100	1.92150100	-2.81046300
In	-4.63285500	-0.30893500	0.11176500
Cl	-4.23275500	-0.84820600	-2.22121500
Cl	-5.40693800	1.93832400	-0.25815600
Cl	-6.21371800	-1.56899400	1.27828600
C	1.56066400	-0.64023400	-2.89759400
C	2.52830800	-1.79619400	-3.01102000
H	2.01348900	-2.69443300	-3.36561100
H	3.32846300	-1.58481400	-3.72472000

Rh-cry
0 1

Rh	1.72120000	5.38290000	10.43060000
O	3.17610000	4.54640000	9.27030000
O	0.71800000	6.06800000	8.78150000
O	2.02620000	2.62190000	9.03870000
O	-0.40130000	4.12290000	8.56900000
O	2.91220000	7.30510000	10.87270000
C	3.02570000	3.36470000	8.82830000
C	4.18270000	2.81040000	7.99790000
C	5.44370000	2.84910000	8.87160000
H	5.61930000	3.75390000	9.14090000
H	6.19090000	2.51480000	8.36930000
H	5.31120000	2.30300000	9.64940000
C	3.90210000	1.37620000	7.54710000
H	3.78490000	0.81570000	8.31770000
H	4.64100000	1.05480000	7.02610000
H	3.10290000	1.35900000	7.01480000
C	4.40380000	3.73870000	6.76510000
H	4.72320000	4.59790000	7.08080000
H	5.10140000	3.35320000	6.21120000
C	3.18240000	3.96870000	5.90580000
C	2.90740000	3.17330000	4.79580000
H	3.50820000	2.51200000	4.53690000

C	1.74420000	3.36760000	4.08200000
H	1.55130000	2.81660000	3.35780000
C	0.85210000	4.38240000	4.43270000
H	0.07180000	4.50360000	3.94170000
C	1.12250000	5.20110000	5.49940000
C	2.29840000	4.98860000	6.22150000
H	2.49420000	5.54840000	6.93740000
C	0.17990000	6.31130000	5.93260000
H	-0.29090000	6.64350000	5.15170000
H	0.70340000	7.04180000	6.29470000
C	-0.86300000	5.86870000	6.99310000
C	-1.80960000	4.83340000	6.41540000
H	-1.30880000	4.06570000	6.13070000
H	-2.27490000	5.20810000	5.66340000
H	-2.44430000	4.57080000	7.08600000
C	-1.65740000	7.10280000	7.44910000
H	-2.27720000	6.84850000	8.13710000
H	-2.13960000	7.46690000	6.70320000
H	-1.05260000	7.76350000	7.79360000
C	-0.12380000	5.30000000	8.21140000
C	3.80130000	7.88330000	10.30240000
C	4.14200000	7.57490000	8.88500000
H	4.46430000	6.67330000	8.82210000
H	4.82300000	8.18050000	8.58070000
H	3.35890000	7.67350000	8.33830000
C	4.59770000	8.93990000	10.98430000
H	4.28660000	9.04280000	11.88700000
H	4.49600000	9.77000000	10.51390000
H	5.52370000	8.68680000	10.99150000
Rh	0.51590000	3.34170000	10.20110000
O	-0.93900000	4.17820000	11.36140000
O	1.51910000	2.65660000	11.85020000
O	0.21080000	6.10270000	11.59300000
O	2.63830000	4.60170000	12.06270000
O	-0.67510000	1.41950000	9.75900000
C	-0.78870000	5.35990000	11.80340000
C	-1.94560000	5.91420000	12.63380000
C	-3.20660000	5.87550000	11.76010000
H	-3.38220000	4.97070000	11.49080000
H	-3.95380000	6.20980000	12.26250000
H	-3.07420000	6.42160000	10.98230000
C	-1.66500000	7.34840000	13.08460000
H	-1.54780000	7.90890000	12.31400000
H	-2.40390000	7.66980000	13.60560000

H	-0.86590000	7.36560000	13.61690000
C	-2.16670000	4.98590000	13.86660000
H	-2.48610000	4.12670000	13.55090000
H	-2.86440000	5.37140000	14.42050000
C	-0.94540000	4.75590000	14.72590000
C	-0.67030000	5.55130000	15.83590000
H	-1.27120000	6.21260000	16.09480000
C	0.49290000	5.35700000	16.54970000
H	0.68580000	5.90800000	17.27390000
C	1.38500000	4.34220000	16.19900000
H	2.16520000	4.22100000	16.69000000
C	1.11460000	3.52350000	15.13230000
C	-0.06130000	3.73600000	14.41020000
H	-0.25710000	3.17620000	13.69430000
C	2.05720000	2.41330000	14.69910000
H	2.52790000	2.08110000	15.48000000
H	1.53370000	1.68280000	14.33700000
C	3.10000000	2.85590000	13.63860000
C	4.04670000	3.89120000	14.21630000
H	3.54580000	4.65890000	14.50100000
H	4.51190000	3.51650000	14.96830000
H	4.68130000	4.15380000	13.54570000
C	3.89440000	1.62180000	13.18260000
H	4.51420000	1.87610000	12.49460000
H	4.37660000	1.25770000	13.92850000
H	3.28960000	0.96110000	12.83810000
C	2.36080000	3.42460000	12.42030000
C	-1.56430000	0.84130000	10.32930000
C	-1.90500000	1.14970000	11.74670000
H	-2.22730000	2.05130000	11.80960000
H	-2.58600000	0.54410000	12.05100000
H	-1.12190000	1.05110000	12.29340000
C	-2.36060000	-0.21530000	9.64740000
H	-2.04960000	-0.31820000	8.74470000
H	-2.25900000	-1.04540000	10.11780000
H	-3.28670000	0.03780000	9.64020000

Rh-rea

0 1

Rh	-0.03174000	0.00000000	1.19416400
O	1.40804300	-1.47999600	1.15997400
O	1.40804300	1.47999600	1.15997400
O	1.47665300	-1.45915700	-1.10283500
O	1.47665300	1.45915700	-1.10283500

C	1.84998700	-1.88781700	0.03421000
C	2.90664800	-2.99787700	0.07400700
C	2.24959800	-4.23308700	0.72877900
H	1.87933400	-3.99487800	1.72891600
H	2.97945600	-5.04567200	0.80668300
H	1.40855400	-4.59124700	0.12498300
C	3.38451900	-3.34376900	-1.34285800
H	2.55121400	-3.68861400	-1.95979600
H	4.13360200	-4.14141500	-1.29238100
H	3.82963800	-2.47749800	-1.83548100
C	4.09339300	-2.51999700	0.97174200
H	3.71854000	-2.40972400	1.99356200
H	4.83544300	-3.32709600	0.98077800
C	4.73568400	-1.22401800	0.52516300
C	5.81551000	-1.21014000	-0.36740400
H	6.23582600	-2.14984900	-0.71610900
C	6.35493700	0.00000000	-0.80536700
H	7.19844700	0.00000000	-1.49016400
C	5.81551000	1.21014000	-0.36740400
H	6.23582600	2.14984900	-0.71610900
C	4.73568400	1.22401800	0.52516300
C	4.22404500	0.00000000	0.97391600
H	3.39342100	0.00000000	1.67128800
C	4.09339300	2.51999700	0.97174200
H	4.83544300	3.32709600	0.98077800
H	3.71854000	2.40972400	1.99356200
C	2.90664800	2.99787700	0.07400700
C	3.38451900	3.34376900	-1.34285800
H	3.82963800	2.47749800	-1.83548100
H	4.13360200	4.14141500	-1.29238100
H	2.55121400	3.68861500	-1.95979600
C	2.24959800	4.23308700	0.72877900
H	1.40855400	4.59124700	0.12498300
H	2.97945600	5.04567200	0.80668300
H	1.87933400	3.99487800	1.72891600
C	1.84998700	1.88781700	0.03421000
Rh	0.03174500	0.00000000	-1.19415400
O	-1.40803900	1.47999500	-1.15996500
O	-1.40803900	-1.47999500	-1.15996500
O	-1.47665000	1.45915600	1.10284300
O	-1.47665000	-1.45915600	1.10284300
C	-1.84998600	1.88781500	-0.03420100
C	-2.90664900	2.99787100	-0.07399900
C	-2.24959400	4.23308800	-0.72875300

H	-1.87932000	3.99489000	-1.72888900
H	-2.97945100	5.04567400	-0.80665500
H	-1.40855600	4.59124300	-0.12494500
C	-3.38453500	3.34375000	1.34286300
H	-2.55123900	3.68859300	1.95981400
H	-4.13362100	4.14139400	1.29238600
H	-3.82965800	2.47747300	1.83547500
C	-4.09338300	2.51999700	-0.97175100
H	-3.71851700	2.40972800	-1.99356700
H	-4.83543200	3.32709800	-0.98079400
C	-4.73568200	1.22401800	-0.52518600
C	-5.81552400	1.21014000	0.36736200
H	-6.23584600	2.14984900	0.71606000
C	-6.35495900	0.00000000	0.80531600
H	-7.19848200	0.00000000	1.49009800
C	-5.81552400	-1.21014000	0.36736200
H	-6.23584600	-2.14984900	0.71606000
C	-4.73568200	-1.22401800	-0.52518600
C	-4.22403600	0.00000000	-0.97393000
H	-3.39340000	0.00000000	-1.67128800
C	-4.09338300	-2.51999700	-0.97175200
H	-4.83543200	-3.32709800	-0.98079400
H	-3.71851600	-2.40972900	-1.99356700
C	-2.90664900	-2.99787100	-0.07399900
C	-3.38453500	-3.34375000	1.34286300
H	-3.82965800	-2.47747300	1.83547500
H	-4.13362100	-4.14139400	1.29238600
H	-2.55123900	-3.68859300	1.95981400
C	-2.24959400	-4.23308800	-0.72875300
H	-1.40855500	-4.59124300	-0.12494500
H	-2.97945100	-5.04567400	-0.80665500
H	-1.87932000	-3.99489000	-1.72888900
C	-1.84998600	-1.88781500	-0.03420100

Rh-sim

0 1

Rh	0.17397700	0.00000700	1.18333500
O	1.59803100	-1.47772800	0.89801800
O	1.59804600	1.47772200	0.89801700
O	1.28605100	-1.43736200	-1.34690800
O	1.28606300	1.43733900	-1.34690800
C	1.87505600	-1.84131000	-0.29028000
C	3.00266200	-2.84067800	-0.47575800
C	4.25961000	-2.55138700	0.36701500

H	3.95911500	-2.46210300	1.41708100
H	4.91485000	-3.42771800	0.29751600
C	4.25964400	2.55137100	0.36698100
H	4.91487900	3.42770400	0.29744500
H	3.95917800	2.46210800	1.41705700
C	3.00267900	2.84065200	-0.47577200
C	1.87507300	1.84129100	-0.29028200
Rh	-0.17395700	-0.00000700	-1.18347700
O	-1.59801600	1.47771400	-0.89815400
O	-1.59801000	-1.47772700	-0.89816400
O	-1.28603000	1.43735300	1.34677100
O	-1.28606300	-1.43730800	1.34676700
C	-1.87503100	1.84131000	0.29014600
C	-3.00255700	2.84077700	0.47559400
C	-4.25969800	2.55130600	-0.36682700
H	-3.95948700	2.46179500	-1.41695500
H	-4.91489700	3.42767400	-0.29735200
C	-4.25974100	-2.55128900	-0.36680500
H	-4.91492300	-3.42766700	-0.29729600
H	-3.95956100	-2.46178900	-1.41694300
C	-3.00258200	-2.84073900	0.47560200
C	-1.87505000	-1.84128700	0.29013800
H	-3.23816700	2.88254500	1.54277900
H	-2.59726800	3.82030600	0.19161100
H	2.59738700	3.82030200	-0.19221800
H	3.23850200	2.88206300	-1.54292800
H	2.59735600	-3.82032400	-0.19221200
H	3.23851100	-2.88209200	-1.54290800
H	-3.23817400	-2.88248600	1.54279200
H	-2.59730900	-3.82027900	0.19163000
C	-5.05257600	1.29718300	0.04657600
H	-5.39466500	1.40037900	1.08612300
H	-5.95907700	1.24750400	-0.57176700
C	-4.25297800	-0.00000100	-0.11524300
H	-3.76911400	-0.00001800	-1.09763000
H	-3.44318400	-0.00000800	0.62042300
C	-5.05262700	-1.29717600	0.04658500
H	-5.95912100	-1.24748400	-0.57176600
H	-5.39472100	-1.40038600	1.08613000
C	5.05258900	1.29710400	-0.04590200
H	5.39532000	1.40019500	-1.08524700
H	5.95869300	1.24726800	0.57300200
C	4.25277300	-0.00000600	0.11545800
H	3.76828400	0.00000300	1.09752400

H	3.44339600	-0.00000100	-0.62074600
C	5.05255300	-1.29712200	-0.04589200
H	5.95866900	-1.24729000	0.57299400
H	5.39526700	-1.40021900	-1.08524300

Rh-rea-cb

0 1

Rh	0.05566100	0.02556400	0.58421000
O	-1.29901300	1.56082800	0.22697300
O	-1.46594600	-1.36536700	0.71082400
O	-1.36750500	1.20052900	-2.00750100
O	-1.49736100	-1.68260500	-1.53351500
C	-1.71726700	1.80754600	-0.95660100
C	-2.71941800	2.96477100	-1.06839400
C	-1.98270600	4.24885700	-0.62678400
H	-1.60172900	4.14830700	0.39264000
H	-2.66578600	5.10385300	-0.66725800
H	-1.13808800	4.45931100	-1.29207600
C	-3.21533600	3.10882700	-2.51331900
H	-2.38127000	3.30220500	-3.19197200
H	-3.91933100	3.94565200	-2.57784600
H	-3.71864200	2.20184800	-2.85327200
C	-3.90336000	2.69726300	-0.08444800
H	-3.50296000	2.71566600	0.93359300
H	-4.59952400	3.53910400	-0.18012800
C	-4.63089800	1.39023400	-0.31656200
C	-5.75075500	1.31096400	-1.15453100
H	-6.13708300	2.21303700	-1.62192100
C	-6.37267700	0.08351200	-1.38771600
H	-7.24507300	0.03340000	-2.03341500
C	-5.87893100	-1.08005400	-0.79589600
H	-6.36439100	-2.03383900	-0.98581300
C	-4.76091800	-1.02770500	0.04674800
C	-4.16187400	0.21593500	0.28612800
H	-3.29769400	0.27140700	0.93663400
C	-4.17618500	-2.28015000	0.66398900
H	-4.95794600	-3.03774900	0.79375500
H	-3.78096000	-2.04883900	1.65685900
C	-3.02639800	-2.94211800	-0.16013500
C	-3.53816100	-3.45331800	-1.51350700
H	-3.94531400	-2.63964700	-2.11688400
H	-4.32592500	-4.19712500	-1.35138400
H	-2.73085400	-3.91983700	-2.08280300
C	-2.42989400	-4.10553500	0.66285500

H	-1.61411600	-4.58978000	0.11508400
H	-3.20018600	-4.85958200	0.85635700
H	-2.03745100	-3.74716600	1.61799800
C	-1.90830500	-1.91093000	-0.36164300
Rh	0.02301200	-0.31081800	-1.84538200
O	1.40019900	-1.83011000	-1.56816100
O	1.53151300	1.11412200	-1.94276900
O	1.41233800	-1.51136700	0.67254400
O	1.59006900	1.37682200	0.30943200
C	1.78703000	-2.10486800	-0.39210200
C	2.78627400	-3.25666400	-0.23017800
C	2.09191600	-4.53705600	-0.74359900
H	1.77102700	-4.41848400	-1.78147300
H	2.78189100	-5.38511000	-0.68086800
H	1.21220600	-4.77216500	-0.13435900
C	3.19317100	-3.42765200	1.24015600
H	2.32116100	-3.64015200	1.86317400
H	3.89786900	-4.26157200	1.33001200
H	3.66583100	-2.52498500	1.63120100
C	4.02601500	-2.95349700	-1.13135000
H	3.69523600	-2.96516900	-2.17415300
H	4.72934100	-3.78451200	-1.00183100
C	4.71138300	-1.63746200	-0.83254900
C	5.76425700	-1.55188000	0.08782100
H	6.12708000	-2.45381900	0.57365600
C	6.35090400	-0.31974900	0.37868500
H	7.17302000	-0.26605300	1.08698100
C	5.88691700	0.84313400	-0.23773900
H	6.34699600	1.80069700	-0.00735100
C	4.83460400	0.78518700	-1.16090900
C	4.27186100	-0.46290100	-1.45602100
H	3.46023900	-0.51853600	-2.17351500
C	4.27634900	2.03526400	-1.80650300
H	5.05947800	2.79730800	-1.89829900
H	3.92889700	1.80105800	-2.81702900
C	3.08699700	2.68788700	-1.03185500
C	3.53675000	3.21066300	0.33926100
H	3.93649700	2.40359700	0.95670100
H	4.31507300	3.97033600	0.20880000
H	2.69580900	3.65971300	0.87295900
C	2.50934000	3.84451300	-1.87580700
H	1.66972200	4.32190100	-1.35832700
H	3.27904000	4.60518800	-2.04439100
H	2.15475700	3.48068200	-2.84304800

C	1.97898500	1.63687600	-0.87789200
C	0.00371300	0.36760500	2.50962700
C	1.11076600	1.05384000	3.20100400
O	1.98491200	0.39697500	3.72735000
C	-1.10069000	-0.16937400	3.33080500
O	-0.93214800	-1.13554700	4.04474900
O	1.03555300	2.38413600	3.14898000
O	-2.24713300	0.49047900	3.13716700
C	2.14807400	3.08301600	3.75380900
H	3.08096300	2.79405100	3.26668500
H	1.94232900	4.14065300	3.59663400
H	2.20565800	2.85300700	4.82045200
C	-3.40294200	-0.02608300	3.83481800
H	-4.26296000	0.40247300	3.32222700
H	-3.42230600	-1.11578700	3.78155100
H	-3.37620200	0.28735400	4.88159500

Rh-sim-cb

0 1

Rh	-0.06010000	0.35282300	-0.28336000
O	-1.51430900	-0.69269400	-1.32729900
O	-1.33747200	0.56295100	1.35383100
O	-0.92185900	-2.65722800	-0.35360800
O	-0.77332800	-1.44086700	2.25366900
C	-1.65119100	-1.94557300	-1.10623900
C	-2.80005300	-2.63939100	-1.81958600
C	-4.12345900	-1.85305900	-1.81741500
H	-3.92368100	-0.85280200	-2.21683200
H	-4.80787800	-2.34413800	-2.51914700
C	-3.86133400	0.37411700	2.75930400
H	-4.46163500	0.66488700	3.62962900
H	-3.65582000	1.28864400	2.19151600
C	-2.52283600	-0.18444700	3.27828400
C	-1.45755400	-0.37584700	2.21125400
Rh	0.59335400	-1.77364500	0.74605900
O	2.03776900	-0.69647600	1.79697200
O	1.90840800	-2.03489100	-0.83637700
O	1.46816900	1.25338400	0.78213600
O	1.29338600	-0.06428100	-1.77380400
C	2.19014700	0.53658100	1.56015200
C	3.33273400	1.26245600	2.25244900
C	4.67281600	0.50361100	2.23214100
H	4.50541300	-0.50031600	2.63824800
H	5.35638800	1.01574400	2.91977400

C	4.41390300	-1.78273600	-2.31769200
H	5.00675100	-2.05080500	-3.20021200
H	4.26241100	-2.70406700	-1.74408200
C	3.04172500	-1.28649200	-2.81091700
C	2.00245200	-1.12831600	-1.71471000
H	3.43121600	2.25107300	1.79417800
H	3.01765400	1.41221000	3.29291800
H	-2.10157600	0.51458800	4.01196000
H	-2.65721000	-1.14493200	3.78413600
H	-2.47354800	-2.79136400	-2.85618600
H	-2.92005100	-3.62773300	-1.36693200
H	3.12613300	-0.32987900	-3.33399300
H	2.62886200	-2.01221000	-3.52316000
C	5.33963000	0.39110100	0.84843600
H	5.55181200	1.39559700	0.45521100
H	6.31343100	-0.10023000	0.97791800
C	4.49930900	-0.40082300	-0.15957900
H	4.13547000	-1.31430500	0.32212700
H	3.60992400	0.18701800	-0.40689400
C	5.20821200	-0.77438800	-1.46639200
H	6.18708700	-1.21713500	-1.23751300
H	5.40956000	0.13117100	-2.05598000
C	-4.68149800	-0.59404800	1.88550400
H	-4.94349000	-1.48967000	2.46629600
H	-5.63272300	-0.10366900	1.63487400
C	-3.95852900	-0.99420600	0.59461200
H	-3.53248700	-0.09724400	0.13811400
H	-3.10825100	-1.63157600	0.85785400
C	-4.81261100	-1.72862700	-0.44529500
H	-5.75990900	-1.18966300	-0.58746700
H	-5.08269000	-2.72752400	-0.07496900
C	-0.67811000	2.08856500	-0.94811000
C	-1.94309300	2.23967400	-1.69091000
O	-1.93518900	2.46041300	-2.88418200
C	0.14690300	3.29011100	-0.71474000
O	0.89945100	3.69285300	-1.57670300
O	-3.02297700	2.07264300	-0.92139300
O	-0.00843600	3.79372000	0.51053600
C	-4.29150300	2.13958100	-1.60818200
H	-4.30849300	1.43489700	-2.44184200
H	-5.03578700	1.87068400	-0.85992700
H	-4.46663400	3.15061900	-1.98429900
C	0.94558100	4.81105100	0.88436800
H	0.65890700	5.12059300	1.88828100

H	1.95202400	4.38535100	0.88378900
H	0.90363500	5.65361700	0.19050000

TS1-A

0 1

Rh	2.14255800	0.16517300	-0.34233500
O	1.31795500	1.17921600	1.28387100
O	3.15910600	1.85167700	-0.98521300
O	3.12232900	0.74795600	2.58589200
O	4.89392700	1.44433200	0.42113500
C	2.00692200	1.30874600	2.34696000
C	1.43750000	2.21382200	3.42956400
C	0.78829400	3.50465100	2.89628100
H	0.08670700	3.22960600	2.10262300
H	0.20170100	3.95068400	3.70914900
C	3.95022500	4.64005700	-0.94708300
H	4.47555500	5.47175000	-1.43139200
H	3.13049800	4.35073800	-1.61065900
C	4.92524400	3.45310100	-0.85451400
C	4.27290800	2.14445900	-0.44429300
Rh	4.05492000	-0.31188400	1.06772800
O	4.91155900	-1.28244300	-0.55099500
O	3.10967900	-2.07353000	1.65618300
O	3.06846100	-0.91410800	-1.82256500
O	1.30872300	-1.60608600	0.36255900
C	4.22979000	-1.39791500	-1.61898200
C	4.83346700	-2.18694100	-2.76724300
C	5.45252800	-3.53645300	-2.35913700
H	6.19129300	-3.35541500	-1.57005100
H	5.99989000	-3.92821400	-3.22454400
C	2.36792000	-4.89312600	1.46696200
H	1.86325300	-5.75282000	1.92347900
H	3.21240700	-4.63709300	2.11651800
C	1.37687400	-3.71598600	1.46656000
C	1.98892900	-2.36103600	1.13799800
H	4.05564000	-2.31442800	-3.52539200
H	5.61294100	-1.55007800	-3.20387300
H	5.37642600	3.28031500	-1.83985100
H	5.74002000	3.65338000	-0.15276500
H	0.68068200	1.62635300	3.96501100
H	2.23896800	2.43064900	4.14141800
H	0.55455500	-3.88827700	0.76696000
H	0.92572600	-3.62636200	2.46285500
C	4.44503600	-4.59738700	-1.87856600

H	3.71860000	-4.80794900	-2.67699900
H	4.98955600	-5.53532000	-1.70430300
C	3.71094500	-4.19067300	-0.59605900
H	4.43728700	-3.79653900	0.12228700
H	3.04061500	-3.35805600	-0.82732800
C	2.89476700	-5.29708800	0.07817600
H	3.51886600	-6.19347100	0.19353200
H	2.05308200	-5.59028200	-0.56639600
C	3.37392500	5.12510000	0.39678800
H	4.18783900	5.48986200	1.03955200
H	2.73110900	5.99392300	0.19664500
C	2.56409700	4.05310700	1.13557600
H	1.87898200	3.57060100	0.43305200
H	3.25279200	3.27174100	1.47307900
C	1.76953300	4.55504600	2.34587600
H	1.18950700	5.44284700	2.05893800
H	2.45439600	4.87857300	3.14251800
C	-0.01505800	0.68426900	-1.77216000
C	0.16187100	2.01825700	-2.41550000
O	0.07086100	2.25837600	-3.60318100
C	0.21946600	-0.50793800	-2.60872100
O	0.80774000	-0.55491100	-3.66593400
O	0.37112200	2.97701200	-1.48532200
O	-0.29906500	-1.64112800	-2.01182500
C	0.36666800	4.32220000	-1.98548000
H	-0.61672100	4.57104500	-2.38863000
H	0.58689900	4.95361900	-1.12550300
H	1.12353700	4.44336400	-2.76352700
C	0.20903900	-2.87154400	-2.54055900
H	-0.19567500	-3.65794600	-1.90074000
H	-0.10701400	-3.01746800	-3.57755300
H	1.30050600	-2.86965800	-2.49668200
C	-5.87435200	-1.58367800	-1.12682600
C	-5.69712600	-0.44141300	-1.96182700
C	-6.57758200	0.64660700	-1.82055400
C	-7.56958300	0.58925400	-0.84901600
C	-7.71012700	-0.53911300	-0.01537200
C	-6.86864600	-1.63982700	-0.14321700
C	-4.08713300	-1.94627800	-2.42450700
C	-4.53022900	-0.69333400	-2.77259900
H	-6.46846100	1.53387800	-2.43389600
H	-8.23999200	1.43297300	-0.71941100
H	-8.48701200	-0.54805900	0.74321600
H	-6.97225500	-2.50306700	0.50591400

H	-3.22490800	-2.48641000	-2.79281200
N	-4.89837700	-2.50904300	-1.45423000
C	-4.19799700	-3.40377700	1.89600600
C	-2.44887300	-2.64622000	0.43416800
C	-3.46980600	-2.97211900	3.00332200
H	-5.16771100	-3.87176600	2.04921800
C	-1.71131100	-2.20431900	1.52928400
H	-2.02409000	-2.50561300	-0.55472900
C	-2.22810300	-2.34999400	2.82005300
H	-3.85318400	-3.09171400	4.01106100
H	-0.73506600	-1.77309000	1.36349000
O	-1.58494700	-1.91311500	3.94339700
C	-3.82376000	0.23819000	-3.71368200
H	-2.91630900	-0.24731700	-4.08948100
H	-4.43361000	0.46043800	-4.59899600
C	-3.46227200	1.61756600	-3.11359100
H	-2.75251200	2.12408600	-3.77414600
H	-4.35426000	2.23311500	-3.02051200
N	-2.84490700	1.56017600	-1.76234700
C	-1.98439000	0.57490300	-1.43531500
C	-0.86591000	0.62818300	-0.49643000
H	-2.17305200	-0.37948000	-1.90141100
H	-0.72578700	-0.27300900	0.08183200
H	-0.75598600	1.52480100	0.10008800
S	-3.28204200	2.82421500	-0.61073400
O	-4.59405200	3.27680500	-1.07670500
O	-2.17907300	3.77545200	-0.47841800
C	-3.43886500	1.93028200	0.92724700
C	-4.33331500	0.86508100	1.03713100
C	-2.73125200	2.40751400	2.03228400
C	-4.51555900	0.27184000	2.28226200
H	-4.87833700	0.50648000	0.17527900
C	-2.93537400	1.80283800	3.27005300
H	-2.04983600	3.24158600	1.92022400
C	-3.83531300	0.73606900	3.41819400
H	-5.20088000	-0.56441800	2.36592200
H	-2.39824400	2.17734200	4.13746700
C	-4.05267000	0.09965900	4.76751800
H	-4.01590500	0.84578600	5.56724400
H	-5.01975300	-0.40787500	4.81673300
H	-3.27347900	-0.64414100	4.96389400
C	-0.47688800	-1.02837100	3.77343700
H	0.40033600	-1.54012500	3.36291100
H	-0.73455300	-0.18595500	3.12068100

H	-0.23365200	-0.66132200	4.77178800
C	-3.70605800	-3.23759800	0.59494100
C	-4.51304800	-3.64909300	-0.62287800
H	-3.93900000	-4.32878600	-1.26122100
H	-5.42043000	-4.18287600	-0.32493100

TS1-B

0 1

Rh	2.09571400	0.15591300	-0.15762700
O	1.26776800	1.05342300	1.53666100
O	2.69929800	1.96988900	-0.93573900
O	3.26072100	1.04008500	2.61720200
O	4.61331300	1.97560700	0.27322500
C	2.03335000	1.35369100	2.51116000
C	1.43249600	2.15367200	3.65527300
C	0.44042700	3.24238700	3.21260000
H	-0.29437300	2.77313600	2.55344000
H	-0.10048200	3.59015700	4.10159200
C	2.81947300	4.85436200	-1.01334300
H	3.09207600	5.75443500	-1.57749200
H	2.01075200	4.35912000	-1.56007100
C	4.05266000	3.92773000	-0.99043000
C	3.76833000	2.51446700	-0.51241800
Rh	4.19402900	0.10540200	1.03570000
O	5.04255100	-0.77541300	-0.64030800
O	3.62636400	-1.75741500	1.77627500
O	3.04645300	-0.79498500	-1.71571200
O	1.63829500	-1.67392400	0.68846200
C	4.28946500	-1.05988100	-1.62355900
C	4.91013700	-1.79401900	-2.79910000
C	5.72788700	-3.03972700	-2.40770800
H	6.49417200	-2.73591900	-1.68552200
H	6.25523900	-3.38653900	-3.30433800
C	3.29967900	-4.67718600	1.75255200
H	2.99383900	-5.58728000	2.28212400
H	4.18662000	-4.29209700	2.26839100
C	2.16380600	-3.64801600	1.90629000
C	2.50936200	-2.24872800	1.42271900
H	4.10686100	-2.04956700	-3.49571800
H	5.56897300	-1.07633600	-3.30334700
H	4.45320300	3.83226000	-2.00853000
H	4.85377000	4.33717500	-0.36807000
H	0.91513700	1.43413200	4.30216000
H	2.25925600	2.57036600	4.23759100

H	1.25952800	-3.96897200	1.38175700
H	1.91247800	-3.56134400	2.97115800
C	4.91006200	-4.20508200	-1.82018600
H	4.15224300	-4.53200600	-2.54709800
H	5.58614100	-5.05900600	-1.67825800
C	4.24386700	-3.85572800	-0.48403400
H	4.96960900	-3.32957200	0.14514000
H	3.43600400	-3.14340900	-0.67339500
C	3.67266300	-5.04039400	0.30340400
H	4.41639700	-5.84809300	0.33699400
H	2.79469800	-5.45130000	-0.21500000
C	2.29062300	5.27079700	0.37086100
H	3.07524200	5.80252700	0.92896300
H	1.47397300	5.98908300	0.22066400
C	1.77602100	4.08385100	1.19051300
H	1.10314800	3.48851900	0.56492200
H	2.62800500	3.44044100	1.43191900
C	1.06796600	4.45253700	2.49754100
H	0.27048300	5.17774600	2.28670000
H	1.77128900	4.95576100	3.17656300
C	-0.14211100	0.38339800	-1.61185800
C	0.26398400	1.62418700	-2.31507100
O	-0.02157800	2.73388200	-1.89831700
C	0.02700500	-1.00730700	-2.08202800
O	-0.43368700	-1.96715000	-1.46993500
O	0.96896000	1.42020400	-3.44404300
O	0.67911400	-1.14950000	-3.24660000
C	1.49527600	2.60591700	-4.05353500
H	2.26572400	3.03946900	-3.41182600
H	1.92796900	2.27944600	-4.99981000
H	0.70964100	3.34709500	-4.22215100
C	1.02038300	-2.49859100	-3.58691400
H	1.53728100	-2.43316800	-4.54471700
H	1.68253600	-2.91886300	-2.82546000
H	0.12768000	-3.12400000	-3.67509900
C	-5.92550600	-1.76749600	-1.14459800
C	-5.67757900	-0.67890200	-2.03283600
C	-6.56755600	0.41032200	-2.03904300
C	-7.63885000	0.41122500	-1.15365700
C	-7.84824100	-0.66054800	-0.26190100
C	-6.99889700	-1.76264500	-0.24617500
C	-4.04401600	-2.21089500	-2.27187500
C	-4.45013400	-0.98005600	-2.72848000
H	-6.40579200	1.25731500	-2.69586500

H	-8.31780700	1.25797600	-1.13706800
H	-8.68602300	-0.62424000	0.42805000
H	-7.15972100	-2.58329000	0.44530200
H	-3.15012800	-2.76427100	-2.52668000
N	-4.93304300	-2.71258300	-1.33872900
C	-4.34566600	-3.29322200	2.05004100
C	-2.47609100	-2.98908100	0.57283100
C	-3.59193600	-2.79249200	3.10825300
H	-5.37137300	-3.60760500	2.22942700
C	-1.70869400	-2.47098800	1.61502100
H	-2.01207800	-3.05676100	-0.40269500
C	-2.28016600	-2.34852500	2.88779300
H	-4.00791200	-2.71393300	4.10659600
H	-0.68498200	-2.17870600	1.41019400
O	-1.63748600	-1.80959800	3.96677000
C	-3.66625500	-0.11718300	-3.67490600
H	-2.72073400	-0.61881300	-3.91213700
H	-4.18781800	0.00974800	-4.63317000
C	-3.38159500	1.31906100	-3.17781000
H	-2.66097400	1.79311700	-3.85416100
H	-4.29518700	1.90942500	-3.19707800
N	-2.83977000	1.41143300	-1.80383000
C	-2.02824100	0.44297700	-1.31454100
C	-0.96469200	0.58866300	-0.33025000
H	-2.28241600	-0.57257200	-1.58469900
H	-0.88321300	-0.22202500	0.38273200
H	-0.81004200	1.57181500	0.09661700
S	-3.39231700	2.75916000	-0.79957000
O	-4.69193700	3.09992800	-1.38682300
O	-2.35845000	3.77815400	-0.65784600
C	-3.61483000	1.96696500	0.78600800
C	-4.52944100	0.92426300	0.93539900
C	-2.89796100	2.47146500	1.87166400
C	-4.71058400	0.37224900	2.20055400
H	-5.08964100	0.55245700	0.08833400
C	-3.09461000	1.90479900	3.12745700
H	-2.20768500	3.29290900	1.72283300
C	-4.00310200	0.85187900	3.31311200
H	-5.41041800	-0.44802700	2.31721900
H	-2.53750800	2.29130100	3.97658900
C	-4.19115200	0.24788600	4.68144000
H	-4.23863100	1.02397800	5.45218000
H	-5.10791300	-0.34490200	4.73542000
H	-3.34858800	-0.41065800	4.91770900

C	-0.40038600	-1.14195400	3.73339100
H	0.38091700	-1.83599000	3.40491800
H	-0.49819500	-0.34852400	2.98321300
H	-0.11146300	-0.71102600	4.69346100
C	-3.80331200	-3.38225000	0.76218400
C	-4.62850700	-3.82224900	-0.43129300
H	-4.09423400	-4.57646900	-1.01815900
H	-5.57365800	-4.27244300	-0.11473300

TS1-C
0 1

Rh	2.10025400	0.17157200	-0.17739100
O	1.29012700	0.98985400	1.56922800
O	2.64709700	2.02791000	-0.88550700
O	3.30981400	0.97469100	2.60139300
O	4.59635300	2.01573800	0.26535200
C	2.07341400	1.26190200	2.53781200
C	1.48276600	1.99068500	3.73403700
C	0.46894300	3.08751500	3.36745200
H	-0.27519900	2.64022100	2.70409900
H	-0.05453400	3.38691700	4.28419700
C	2.71175300	4.91267500	-0.84617700
H	2.95245400	5.84028500	-1.37933400
H	1.89933700	4.42443200	-1.39440400
C	3.96285700	4.01111300	-0.89228600
C	3.71907300	2.57296400	-0.46959000
Rh	4.22842700	0.11156600	0.96833100
O	5.04170400	-0.70593000	-0.76027400
O	3.72185900	-1.78634100	1.66025600
O	3.01578400	-0.71577000	-1.77884900
O	1.70565000	-1.70829200	0.62443500
C	4.26316300	-0.97080500	-1.73035300
C	4.84695700	-1.66311200	-2.94887000
C	5.72072700	-2.88975000	-2.62622100
H	6.50800000	-2.58113800	-1.92879900
H	6.21934800	-3.19647600	-3.55329700
C	3.45787300	-4.70897300	1.55884200
H	3.18380700	-5.64009900	2.06893900
H	4.34670400	-4.31764900	2.06638300
C	2.30409800	-3.70925700	1.76487200
C	2.60813000	-2.28789300	1.31654300
H	4.01516000	-1.92535900	-3.60859100
H	5.45756900	-0.91458800	-3.46914100
H	4.34018300	3.96746200	-1.92265400

H	4.77100500	4.40998300	-0.27204300
H	0.98551500	1.22929800	4.34920300
H	2.31368100	2.38717800	4.32431500
H	1.39707000	-4.03700500	1.24847300
H	2.06851500	-3.65764100	2.83569100
C	4.96529200	-4.09671800	-2.03920600
H	4.19169200	-4.43011200	-2.74594100
H	5.67349700	-4.93085500	-1.94270600
C	4.33726600	-3.80308300	-0.67207400
H	5.07198000	-3.28454200	-0.04721000
H	3.51380500	-3.09796100	-0.81256800
C	3.80725500	-5.02144500	0.09180800
H	4.56624000	-5.81531200	0.08473200
H	2.92651600	-5.43536600	-0.42060400
C	2.21204400	5.26337300	0.56735000
H	3.00168700	5.78777800	1.12528100
H	1.37844400	5.97086900	0.46788900
C	1.74214300	4.03563200	1.35279400
H	1.06174100	3.45383400	0.72275700
H	2.61084000	3.39815900	1.54389800
C	1.06410500	4.33683300	2.69298400
H	0.25313000	5.06070000	2.53495700
H	1.77906200	4.81831200	3.37553100
C	-0.06870500	0.43145300	-1.63873300
C	0.28020700	1.69043700	-2.33570300
O	-0.10646200	2.78468600	-1.95707600
C	0.11864500	-0.88698400	-2.28559500
O	0.51080900	-1.11753900	-3.40921700
O	1.07829800	1.51634800	-3.40304000
O	-0.23499000	-1.89918000	-1.42677000
C	1.54482700	2.72248400	-4.01708000
H	2.21616700	3.25173100	-3.33667600
H	2.08158000	2.40445700	-4.91126700
H	0.71251000	3.38132000	-4.27981300
C	0.20936700	-3.20132500	-1.82813600
H	-0.09111700	-3.87451100	-1.02478900
H	-0.24692500	-3.50092500	-2.77600700
H	1.29621300	-3.20267600	-1.93919800
C	-6.02667700	-1.56796600	-1.21700200
C	-5.74005800	-0.42630600	-2.02222000
C	-6.55620300	0.71296900	-1.90114500
C	-7.58991600	0.70607800	-0.97276700
C	-7.83599600	-0.42161400	-0.16344100
C	-7.06199800	-1.57229700	-0.27492900

C	-4.21901500	-2.02973600	-2.45308000
C	-4.55697900	-0.74134500	-2.78777300
H	-6.36265800	1.60165100	-2.48990700
H	-8.20932600	1.58979300	-0.85706400
H	-8.64239900	-0.39095900	0.56310600
H	-7.25138700	-2.43698300	0.35281900
H	-3.39080600	-2.62569200	-2.81308800
N	-5.10529800	-2.55473600	-1.52656300
C	-4.38186500	-3.48035700	1.81947300
C	-2.63382800	-2.91951000	0.27680800
C	-3.56763700	-3.08987600	2.88093700
H	-5.38267600	-3.85262700	2.02609600
C	-1.80158000	-2.52377100	1.32111400
H	-2.25357100	-2.83873400	-0.73267700
C	-2.28515300	-2.57865000	2.63489400
H	-3.91706800	-3.14715500	3.90607100
H	-0.79563300	-2.19013600	1.09395000
O	-1.58059100	-2.15364000	3.72346400
C	-3.74786900	0.15032600	-3.68547300
H	-2.83469200	-0.37742100	-3.98409800
H	-4.28185900	0.37927500	-4.61737700
C	-3.38006400	1.52662300	-3.08363900
H	-2.63758200	2.01329000	-3.72522400
H	-4.25953100	2.16537200	-3.04345800
N	-2.81986900	1.48542300	-1.71443800
C	-2.01834000	0.47917500	-1.31167800
C	-0.91050100	0.57737800	-0.36392000
H	-2.27313000	-0.50863300	-1.67146700
H	-0.82148100	-0.24655600	0.33097900
H	-0.76370000	1.54398300	0.10477500
S	-3.30868000	2.77855700	-0.59206700
O	-4.60577000	3.19987400	-1.12910800
O	-2.24489300	3.75418800	-0.38830800
C	-3.52142300	1.86826200	0.93200900
C	-4.39364500	0.78197800	1.00466500
C	-2.84552700	2.33610700	2.05929400
C	-4.56981100	0.14739500	2.23046400
H	-4.92109800	0.43376200	0.12788200
C	-3.03623200	1.68658300	3.27592700
H	-2.18873900	3.19294600	1.97448300
C	-3.89869200	0.58553800	3.38218900
H	-5.23497700	-0.70715100	2.28449500
H	-2.50844200	2.04496500	4.15549100
C	-4.07520400	-0.11976400	4.70294000

H	-4.06818900	0.59008300	5.53588600
H	-5.01448100	-0.67830300	4.73561600
H	-3.25696100	-0.83115400	4.85916300
C	-0.41956300	-1.35516400	3.48787700
H	0.36866400	-1.91696800	2.97832100
H	-0.65379600	-0.46766100	2.88887200
H	-0.06492600	-1.05126800	4.47405900
C	-3.93346700	-3.38217700	0.49698400
C	-4.81543300	-3.72176200	-0.69116500
H	-4.33178400	-4.46362000	-1.33565500
H	-5.76450800	-4.15557200	-0.36427000

TS2-I

0 1

C	-1.37346500	-0.74527100	-0.16420700
C	-1.89435500	0.28623600	-1.01269900
O	-3.04244400	0.28571000	-1.51864200
C	-2.19241700	-1.93716700	0.00869900
O	-3.40717100	-2.01024500	-0.23539300
O	-1.06357500	1.29897100	-1.29045900
O	-1.50830600	-2.97258400	0.47713800
C	-1.61278100	2.33001900	-2.15586100
H	-2.54291300	2.71803400	-1.73558600
H	-0.83954300	3.09286900	-2.19815700
H	-1.81405300	1.91371600	-3.14505300
C	-2.26018000	-4.14564700	0.85374800
H	-1.52544700	-4.80845900	1.30763000
H	-3.04376500	-3.87418100	1.56423800
H	-2.71537100	-4.60156200	-0.02829700
C	4.67596200	0.86032800	-1.83941000
C	4.20546800	-0.41612300	-2.27026500
C	5.09308100	-1.50652600	-2.28101100
C	6.39836600	-1.31967400	-1.84055600
C	6.84083300	-0.05355100	-1.40246800
C	5.99338100	1.05036900	-1.40467400
C	2.51321300	1.06302700	-2.38614100
C	2.81230000	-0.26113700	-2.60054600
H	4.76437500	-2.48378000	-2.62171600
H	7.09052400	-2.15588100	-1.83749700
H	7.86756400	0.06561900	-1.06964200
H	6.35108200	2.02319900	-1.08239900
H	1.56522600	1.57360600	-2.48512000
N	3.62251100	1.75321700	-1.93698000
C	1.30350700	3.99499000	-0.64542900

C	2.52902500	2.51729300	0.80045800
C	0.20474200	3.95370200	0.22107900
H	1.23823500	4.59582600	-1.55016000
C	1.45448100	2.47097500	1.67297700
H	3.41696100	1.93183900	1.01712300
C	0.25750500	3.14404100	1.36172100
H	-0.69266100	4.51087400	-0.01883300
H	1.49080600	1.87314800	2.57696800
O	-0.78006700	2.90306500	2.19891100
C	1.83374400	-1.33116700	-2.98607300
H	0.86299300	-0.87861600	-3.21709500
H	2.14781000	-1.84562800	-3.90341500
C	1.66139400	-2.45514100	-1.93766300
H	0.86451100	-3.13494100	-2.25852700
H	2.57963000	-3.03283100	-1.85708200
N	1.32465400	-1.98701000	-0.56870800
C	0.60490700	-0.86885700	-0.38534700
C	-0.20436100	-0.47955900	0.77169500
H	0.68837800	-0.12178900	-1.15847700
H	-0.12297100	0.56111800	1.06958300
H	-0.20211000	-1.16110200	1.61521700
S	2.02459500	-2.88124000	0.81057900
O	2.96022300	-3.80294200	0.17032200
O	0.95164600	-3.35749600	1.67666900
C	2.92497000	-1.60620700	1.67453400
C	4.14351200	-1.16673500	1.15402900
C	2.42147500	-1.11339500	2.88041100
C	4.84968000	-0.18747300	1.84707800
H	4.53846000	-1.58009300	0.23426300
C	3.14451100	-0.13277800	3.55520000
H	1.49438900	-1.50239400	3.28549000
C	4.35733600	0.35468500	3.04461900
H	5.79143800	0.16296500	1.43683400
H	2.76122200	0.25798200	4.49359600
C	5.09153300	1.46990500	3.74445600
H	6.16516600	1.43582900	3.54045400
H	4.71863700	2.44146700	3.39640100
H	4.94353400	1.43294900	4.82728500
C	-2.09779800	3.30249700	1.80023800
H	-2.34543700	2.92352500	0.80325500
H	-2.77954900	2.84556900	2.51559100
H	-2.20460600	4.39368100	1.81751500
In	-4.70863200	-0.08674100	-0.19030100
Cl	-5.21323900	2.23617800	-0.55102400

Cl	-3.90805300	0.05292400	2.05908200
Cl	-6.51877300	-1.36241900	-0.90079800
C	2.45994200	3.25816000	-0.39045700
C	3.56526200	3.12560900	-1.41569800
H	3.41374100	3.82564600	-2.24495300
H	4.54305800	3.34645500	-0.98054400
